# Supplementary material for: Seizure First Aid Training For people with Epilepsy (SAFE) frequently attending emergency departments and their significant others: results of a UK multi-centre randomised controlled pilot trial
Source: BMJ Open. 2020 Apr 16;10(4):e035516. doi: 10.1136/bmjopen-2019-035516 (PMC7201300; doi:10.1136/bmjopen-2019-035516)
Supplement: Supplementary data [file bmjopen-2019-035516supp001.pdf]

SUPPLEMENTARY FILES

Supplementary File 1 Search criteria employed at the different recruitment sites to examine their electronic unique attendance record systems to identify potentially suitable persons attending ED for epilepsy

Recruitment site		
**Site**<br>**Aintree University Hospital**	**Site**<br>**Royal Liverpool University Hospital**	**Site**<br>**Arrowe Park Hospital**
Identify those persons who within the free text box 'presenting complaint' are recorded as having visited the ED for a:<br>• "fit"<br>• "epilepsy"<br>• "convulsion"<br>• "seizure"<br><br>And those patients admitted as an emergency from ED to a ward with any of the following primary or secondary ICD-10 diagnoses:<br>• "G40 Epilepsy"<br>• "G41 Status epilepticus"<br>• "G83.8 Other specified paralytic syndromes"<br>• "R56.8 Other and unspecified convulsions"	Identify those persons who attended the ED and were given any of the following presenting complaint-discharge diagnosis profiles:<br>• Presenting complaint "AE20 AEPRC Fit" AND ED discharge diagnosis "02 AEDIG Contusion/ abrasion";<br>• "AE20 AEPRC Fit" AND "04 AEDIG Head injury";<br>• "AE20 AEPRC Fit" AND "05 AEDIG Dislocation";<br>• "AE20 AEPRC Fit" AND "06 AEDIG Sprain/ ligament injury";<br>• "AE20 AEPRC Fit" AND "07 AEDIG Muscle/ tendon injury";<br>• "AE20 AEPRC Fit" AND "10 AEDIG Burns and scalds";<br>• "AE20 AEPRC Fit" AND "15 AEDIG Near Drowning";<br>• "AE20 AEPRC Fit" AND "33 AEDIG Facio-maxillary conditions";<br>• "AE20 AEPRC Fit" AND "38 AEDIG Diagnosis not classifiable".<br>• "241 AEDIG Epilepsy".<br><br>And those patients admitted as an emergency from ED to a ward with any of the following primary or secondary ICD-10 diagnoses:<br>• "G40 Epilepsy"<br>• "G41 Status epilepticus"<br>• "G83.8 Other specified paralytic syndromes"<br>• "R56.8 Other and unspecified convulsions"	Identify those persons who within the free text box 'reason for visit' or 'presenting complaint' are recorded as having visited the ED for:<br>• "fit"<br>• "epilep"<br>• "convuls"<br>• "seiz"<br><br>And those patients admitted as an emergency from ED to a ward with any of the following primary or secondary ICD-10 diagnoses:<br>• "G40 Epilepsy"<br>• "G41 Status epilepticus"<br>• "G83.8 Other specified paralytic syndromes"<br>• "R56.8 Other and unspecified convulsions"

Notes:

As the architectures of the systems and the coding processes at each sites varied the research team worked with local ED teams to identify the search criteria.

Supplementary File 2 CONSORT diagram of eligibility screening for the trial

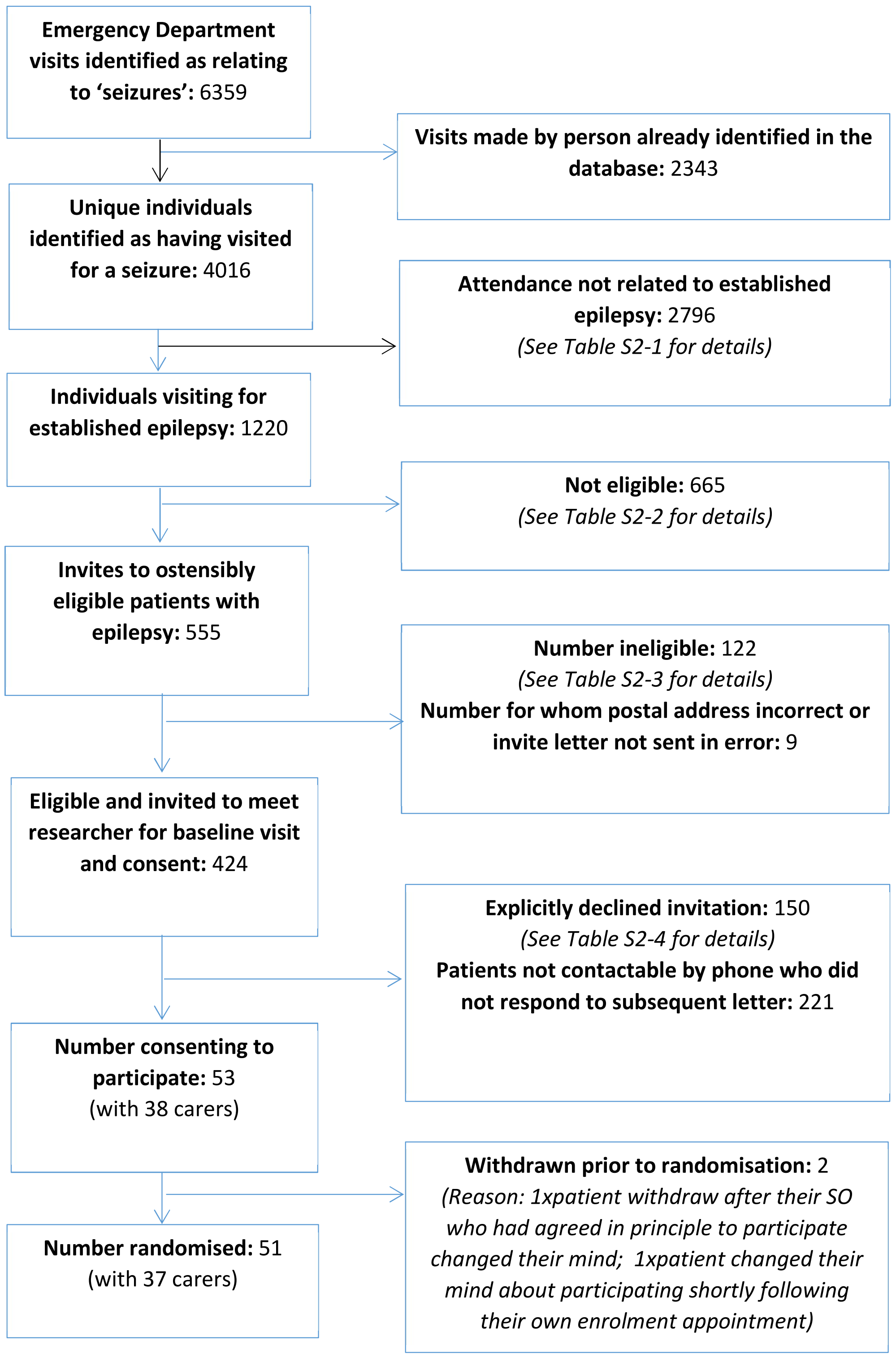

Table S2-1 Attendance at emergency department not related to epilepsy

**Reason**	**Number of participants**			
	**Site 1**	**Site 2**	**Site 3**	**Total**
No confirmed diagnosis of epilepsy/not attending because of epilepsy	737	483	715	1935
Acute symptomatic seizures related to neurological illness or substance abuse	238	240	191	669
Psychogenic non-epileptic seizures	19	55	29	103
Medical record missing	54	5	27	86
Ineligible, but no reason given	2	1	0	3
**Total**	**1050**	**784**	**962**	**2796**

Table S2-2 Other reasons for non-eligibility (individuals visiting for established epilepsy)

**Reason**	**Number of participants**			
	**Site 1**	**Site 2**	**Site 3**	**Total**
Postcode outside catchment area	148	94	60	302
Learning disability likely to impede individuals' capacity to provided signed, informed consent	45	51	52	148
Inability to converse in English and provide signed informed consent	10	24	46	80
Life threatening medical illness	35	26	10	71
Severe psychiatric disorder	11	3	18	32
No fixed abode	10	6	13	29
Participating in another trial	3	0	0	3
**Total**	**262**	**204**	**199**	**665**

Table S2-3 Reasons for non-eligibility (ostensibly eligible individuals)

**Reason**	**Number of participants**			
	**Site 1**	**Site 2**	**Site 3**	**Total**
Has not visited an ED for epilepsy on ≥2 occasions within previous 12 months (as reported by patient)	9	15	24	48
Not able to provide informed consent, participate in SAFE course if randomised or to independently complete questionnaires in English	9	7	7	23
Ineligible, but no reason given[a]	4	7	4	15
Not established diagnosis of epilepsy (<1 year)	3	2	5	10
Moved out of area; postcode no longer within 25 mile catchment area	4	1	3	8
Actual or suspected psychogenic non-epileptic seizures alone or in combination with epilepsy	2	1	4	7
Severe current psychiatric disorders or life-threatening medical illness	1	5	1	7

Not currently being prescribed AED/s	1	0	1	2
Acute symptomatic seizures related to acute neurological illness or substance misuse	0	1	0	1
Participating in another trial	1	0	0	1
**Total**	**34**	**39**	**49**	**122**

Notes:

a. Lack of reason due to patients returning participation slips and no stating actual reason for ineligibility

Table S2-4 Reasons for declining participation (eligible individuals)

**Reason**	**Number of participants**			
	**Site 1**	**Site 2**	**Site 3**	**Total**
Not interested	30	11	23	64
Too busy	11	12	11	34
No reason given	9	10	8	27
Too ill	5	5	9	19
Too well	3	1	2	6
**Total**	**58**	**39**	**53**	**150**

Supplementary File 3 Demographic characteristics of SO participants

**Demographic characteristic**	**Seizure First Aid Training + TAU**	**Treatment as usual (TAU)**	**Total**
Relationship with patient participant	(n=18)	(n=19)	(n=37)
Parent	4 (22.2%)	4 (21.1%)	8 (21.6%)
Son / daughter	2 (11.1%)	4 (21.1%)	6 (16.2%)
Grandparent	0 (0.0%)	0 (0.0%)	0 (0.0%)
Spouse / partner	9 (50.0%)	7 (36.8%)	16 (43.2%)
Sibling	1 (5.6%)	0 (0.0%)	1 (2.7%)
Cousin, aunt / uncle	0 (0.0%)	0 (0.0%)	0 (0.0%)
Niece / nephew	0 (0.0%)	1 (5.3%)	1 (2.7%)
Friend	2 (11.1%)	3 (15.8%)	5 (13.5%)
Other	0 (0.0%)	0 (0.0%)	0 (0.0%)
Missing	0 (0.0%)	0 (0.0%)	0 (0.0%)
Co-habitation with patient participant	(n=18)	(n=19)	(n=37)
Yes	14 (77.8%)	14 (73.4%)	28 (75.7%)
No	4 (22.2%)	5 (26.3%)	9 (24.3%)
Missing	0 (0.0%)	0 (0.0%)	0 (0.0%)
Contact with patient participant (number of days per week)	(n=18)	(n=19)	(n=37)
None	0 (0.0%)	0 (0.0%)	0 (0.0%)
One	1 (5.6%)	0 (0.0%)	1 (2.7%)
Two	0 (0.0%)	1 (5.3%)	1 (2.7%)
Three	0 (0.0%)	2 (10.5%)	2 (5.4%)
Four	0 (0.0%)	0 (0.0%)	0 (0.0%)
Five	0 (0.0%)	0 (0.0%)	0 (0.0%)
Six	0 (0.0%)	0 (0.0%)	0 (0.0%)
Seven (every day)	17 (94.4%)	16 (84.2%)	33 (89.2%)
Missing	0 (0.0%)	0 (0.0%)	0 (0.0%)
Sex: n (%)	(n=18)	(n=19)	(n=37)
Male	6 (33.3%)	9 (47.4%)	15 (40.5%)
Female	12 (67.7%)	10 (52.6%)	22 (59.5%)
Missing	0 (0.0%)	0 (0.0%)	0 (0.0%)
Age at consent into the trial (years)			
N	17 (94.4%)	19 (100.0%)	36 (94.6%)
Mean	41.3	44.9	43.2
Standard deviation	18.66	15.69	17.01
Minimum	17.8	18.1	17.8
Median	43.5	49.7	48.0
Maximum	79.7	71.5	79.7
Missing	1 (5.6%)	0 (0.0%)	1 (2.7%)
Ethnicity: n (%)	(n=18)	(n=19)	(n=37)
White	18 (100.0%)	18 (94.5%)	36 (97.3%)
Asian / Asian British	0 (0.0%)	0 (0.0%)	0 (0.0%)
Black / African / Carribean / Black British	0 (0.0%)	0 (0.0%)	0 (0.0%)
Mixed / multiple ethnic groups	0 (0.0%)	0 (0.0%)	0 (0.0%)
Other ethnic group	0 (0.0%)	1 (5.3%)	1 (2.7%)
Missing	0 (0.0%)	0 (0.0%)	0 (0.0%)
Significant medical history: n (%)	(n=18)	(n=19)	(n=37)
No, none	13 (72.2%)	12 (63.2%)	25 (67.6%)
Yes, a medical condition	5 (27.8%)	6 (31.6%)	11 (29.7%)
Yes, a psychiatric condition	0 (0.0%)	0 (0.0%)	0 (0.0%)
Yes, both medical and psychiatric conditions	0 (0.0%)	1 (5.3%)	1 (2.7%)
Missing	0 (0.0%)	0 (0.0%)	0 (0.0%)

Education: n (%)	(n=18)	(n=19)	(n=37)
O' levels/ GCSEs/ Level 1 or 2 NVQ	9 (50.0%)	14 (73.7%)	23 (62.2%)
A' Levels/ Level 3 NVQ	4 (22.2%)	2 (10.5%)	6 (16.2%)
University degree/ Graduate Certificate or Diploma	5 (27.8%)	3 (15.8%)	8 (21.6%)
Postgraduate university degree (e.g., PGCE, MSc, MA, PhD)	0 (0.0%)	0 (0.0%)	0 (0.0%)
Missing	0 (0.0%)	0 (0.0%)	0 (0.0%)

Supplementary File 4 Measure completeness by assessment point, measure and participant type

Table S4.1 Number of patient participants completing assessment tools

**Study assessment tool and timepoint**	**SAFE + TAU**	**TAU**	**Total**
**Patient Participants: Baseline (T0)**	**(n=26)**	**(n=25)**	**(n=51)**
ED visits (self-report)	24 (92.3%)	21 (84.0%)	45 (88.2%)
Seizure control: Thapar scale	26 (100%)	25 (100%)	51 (100%)
Quality of Life: QOLIE-31P	23 (88.5%)	16 (64.0%)	39 (76.5%)
Distress: HADS [a]	23 (88.5%)	24 (96.0%)	47 (92.2%)
Felt stigma: Stigma of Epilepsy Scale	24 (92.3%)	24 (96.0%)	48 (94.1%)
Health economics: Client Service Receipt Inventory [b]	19 (73.1%)	17 (68.0%)	36 (70.6%)
Health economics: EuroQol-5D	21 (80.8%)	25 (100.0%)	46 (90.2%)
Confidence managing seizures/ epilepsy: Epilepsy Mastery Scale	26 (100.0%)	21 (84.0%)	47 (92.2%)
Fear of seizures:, Epilepsy Knowledge and Management Questionnaire Fears subscale	16 (61.5%)	9 (36.0%)	25 (49.2%)
Knowledge of what to do: Thinking about Epilepsy Questionnaire	26 (100%)	25 (100%)	51 (100%)
**Patient Participants: 6 months (T2)**	**(n=26)**	**(n=23)[c]**	**(n=49)[c]**
Quality of Life: QOLIE-31P	16 (61.5%)	12 (52.2%)	28 (57.1%)
Confidence managing seizures/ epilepsy: Epilepsy Mastery Scale	21 (80.8%)	15 (65.2%)	36 (73.5%)
Seizure control: Thapar scale	16 (61.5%)	15 (65.2%)	28 (57.1%)
**Patient Participants: 12 months (T3)**	**(n=25)[d]**	**(n=22)[d]**	**(n=47)[d]**
ED visits (self-report)	17 (68.0%)	17 (77.3%)	34 (72.3%)
Seizure control: Thapar scale	20 (80.0%)	14 (60.9%)	34 (72.3%)
Quality of Life: QOLIE-31P	10 (40.0%)	8 (36.4%)	18 (38.3%)
Distress: HADS [a]	18 (72.0%)	17 (77.3%)	35 (74.4%)
Felt stigma: Stigma of Epilepsy Scale	18 (72.0%)	17 (77.3%)	35 (74.4%)
Health economics: Client Service Receipt Inventory [b]	13 (52.0%)	10 (45.5%)	23 (48.9%)
Health economics: EuroQol-5D	18 (72.0%)	17 (77.3%)	35 (74.4%)
Confidence managing seizures/ epilepsy: Epilepsy Mastery Scale	18 (72.0%)	16 (72.7%)	34 (72.3%)
Fear of seizures: Epilepsy Knowledge and Management Questionnaire Fears subscale	8 (32.0%)	5 (22.7%)	13 (27.7%)
Knowledge of what to do: Thinking about Epilepsy Questionnaire	18 (72.0%)	17 (77.3%)	35 (74.4%)
Feedback on participation	17 (68.0%)	15 (68.2%)	32 (68.1%)

Notes:

[a] Completeness of the whole HADS scale (both anxiety and depression subscales)

[b] Only six mandatory questions counted. Conditional questions (e.g. if yes, then…) not counted towards total completion

[c] Two patient participants from the TAU group had withdrawn by the T2 visit

[d] Four patient participants (three from the TAU group and one from the SAFE + TAU group) had withdrawn by the T3 visit

[e] Note that whilst some participants did not fully complete all items within a questionnaire, this does not automatically mean their data would need to be excluded from analysis of a change in that domain since for some measures test developers permit imputation when the extent of missing items is low and follows a specific pattern.

Table S4.2 Number of SO participants completing study assessment tools

Study assessment tool and time point	SAFE + TAU	TAU	Total
**Significant other Participants: Baseline (T0)**	**(n=18)**	**(n=19)**	**(n=37)**
Burden: Zarit Caregiver Burden	17 (94.4%)	17 (89.5%)	34 (91.9%)
Distress: HADS [a]	17 (94.4%)	18 (94.7%)	35 (94.6%)
Confidence managing seizures/ epilepsy: Parent Response to Child Illness Scale	18 (100.0%)	19 (100.0%)	37 (100.0%)
Fear of seizures: Epilepsy Knowledge and Management Questionnaire Fears subscale	6 (33.3%)	4 (21.1%)	10 (27.0%)
Knowledge of what to do: Thinking about Epilepsy Questionnaire	18 (100.0%)	19 (100.0%)	37 (100.0%)
**Significant other Participants: 6 months (T2)**	**(n=17)[b]**	**(n=16)[b]**	**(n=33)[b]**
Burden: Zarit Caregiver Burden	15 (88.2%)	8 (50.0%)	23 (69.7%)
Confidence managing seizures/ epilepsy: Parent Response to Child Illness Scale	16 (94.1%)	9 (56.3%)	25 (75.8%)
**Significant other Participants: 12 months (T3)**	**(n=17)[c]**	**(n=15)[c]**	**(n=32)[c]**
Burden: Zarit Caregiver Burden	11 (64.7%)	10 (66.7%)	21 (65.6%)
Distress: HADS [a]	11 (64.7%)	10 (66.7%)	21 (65.6%)
Confidence managing seizures/ epilepsy: Parent Response to Child Illness Scale	11 (64.7%)	10 (66.7%)	21 (65.6%)
Fear of seizures: Epilepsy Knowledge and Management Questionnaire Fears subscale	6 (35.3%)	2 (13.3%)	8 (25.0%)
Knowledge of what to do: Thinking about Epilepsy Questionnaire	15 (88.2%)	13(86.7%)	28 (87.5%)
Feedback on participation	11 (64.7%)	9 (60.0%)	20 (62.5%)

Notes:

[a] Completeness of the whole HADS scale (both anxiety and depression subscales),

[b] Four SO participants (one in the SAFE group and three in the TAU group) had withdrawn by the T2 visit

[c] Five SO participants (one in the SAFE group and four in the TAU group) had withdrawn by the T3 visit

[d] Note that whilst some participants did not fully complete all items within a questionnaire, this does not automatically mean their data would need to be excluded from analysis of a change in that domain since for some measures test developers permit imputation when the extent of missing items is low and follows a specific pattern.

Supplementary File 5 Histogram of number of emergency department attendances in 12 prior to randomisation by n=48 patient participants according to routine data

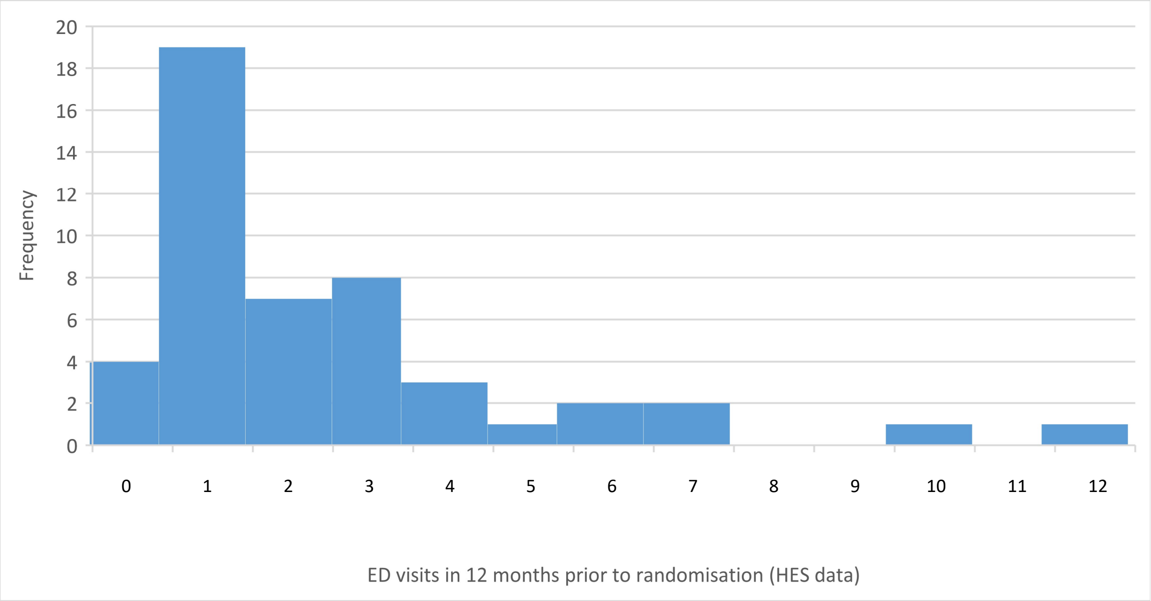

Supplementary File 6 Summary of key milestones in securing ED outcome data from NHS Digital

Period	Project milestone		Milestone in obtaining data from NHS Digital	
	Date	*Note*	*Date*	*Note*
			**28 Sep 2015**	Received feedback on draft Participant Information Sheets and Consent form for trial to ensure compliance.
Trial period	**19 May 2016**	First patient participant randomised		
			**11 Oct 2017**	Participated in NHS Digital training.
			**18 Oct 2017**	Registered as new user of application system.
			**07 Nov 2017**	Delays experienced. Access to application form granted after chasing.
	**Dec 2017**	Final 12-month follow-up completed		
Post -trial			**16 Feb 2018**	Application submitted to NHS Digital
			**28 Feb 2018**	Teleconference with NHS Digital. Provisional opinion given that only minor changes required and likely timeframe to receipt of data 1.5 months.
			**07 Mar 2018**	Received written feedback from NHS Digital after chasing on minor changes required.
			**22 Mar 2018**	Revised application submitted.
			**03 Apr 2018**	NHS Digital submit new query (1) to applicants requesting additional information. (Applicants respond 03 Apr 2018)
			**26 Apr 2018**	NHS Digital submit new query (2) to applicants requesting additional information. (Applicants respond 26 Apr 2018)
			**30 Apr 2018**	NHS Digital submit new query (3) to applicants requesting additional information. (Applicants respond 30 Apr 2018)
			**05 May 2018**	NHS Digital submit new query (4) to applicants requesting additional information. (Applicants respond 05 May 2018).
			**10 May 2018**	NHS Digital submit new query (5) to applicants requesting additional information (in light of new General Data Protection Regulation introduced on May 2018). (Applicants respond 11 May 2018)
			**30 May 2018**	NHS Digital submit new query (6) to applicants requesting additional information (in light of new General Data Protection Regulation introduced on May 2018). (Applicants respond 30 May 2018)
			**30 May 2018**	NHS Digital submit new query (7) to applicants requesting additional information. (Applicants respond 30 May 2018)
			**15 Jun 2018**	NHS Digital confirm Data Approvals Owner will review revised application.
			**27 Jun 2018**	NHS Digital submit new query (8) to applicants requesting additional information and request revisions to trial website in light of new General Data Protection Regulation introduced on May 2018). (Applicants respond 28 Jun 2018)

			**02 Jul 2018**	NHS Digital submit new query (9) to applicants requesting additional information.<br>(Applicants 02 Jul 2018)
			**06 Jul 2018**	NHS Digital notify applicants that Data Approval Owner has rejected application, primarily because: “concerns [over] whether this pilot would yield findings that were statistically valuable to achieve the stated aims given the small numbers”.
			**10 Jul 2018**	Secured confirmation of right to appeal and process.
			**20 Jul 2018**	Applicants submit letter of appeal.
			**25 Jul 2018**	Appeal accepted by NHS Digital.
			**15 Aug 2018**	NHS Digital submit new query (10) to applicants following Independent Group Advising on the Release of Data (IGARD) committee’s review of application.<br>(Applicants respond 16 Aug 2018)
			**16 Aug 2018**	Teleconference with NHS Digital data production team.
			**04 Sep 2018**	NHS Digital send Data Sharing Agreement to applicants.
			**10 Sep 2018**	NHS Digital confirm receipt of completed Data Sharing Agreement.
			**25 Sep 2018**	Applicants securely transfer patient participants’ details to NHS Digital.
			**31 Sep 2018**	NHS Digital release data to applicants.

Supplementary File 7 Adverse events occurring during pilot trial in descending order according to frequency overall [a]

**Adverse event**		**SAFE + TAU**		**TAU**		**Total**	
Category of events (e.g. body system)	Event	Events (n)	Patients [n(%)]	Events (n)	Patients [n(%)]	Events (n)	Patients [n(%)]
Eyes, ear, nose, throat	Problem with eyes and sinuses	0	0 (0.0%)	1	1 (9.1%)	1	1 (5.6%)
Genito-urinary	Overnight hospital admission required due to urinary tract infection (pre existing)	0	0 (0.0%)	1	1 (9.1%)	1	1 (5.6%)
Haematological	Dislocated shoulder	0	0 (0.0%)	1	1 (9.1%)	1	1 (5.6%)
Neoplasia	Change in seizure pattern [b]	1	1 (14.3%)	0	0 (0.0%)	1	1 (5.6%)
	Increase in seizure frequency [b]	1	1 (14.3%)	0	0 (0.0%)	1	1 (5.6%)
Neurological	Increased seizures, medications changed	1	1 (14.3%)	3	3 (27.3%)	4	4 (22.2%)
	Increased seizures (shift patterns at work)	1	1 (14.3%)	0	0 (0.0%)	1	1 (5.6%)
	New seizure type; frequent absence seizures as well as usual seizure types	1	1 (14.3%)	0	0 (0.0%)	1	1 (5.6%)
	Seizure frequency sodium levels requiring in patient monitoring	0	0 (0.0%)	1	1 (9.1%)	1	1 (5.6%)
	Seizures more severe (tonic clonic)	0	0 (0.0%)	1	1 (9.1%)	1	1 (5.6%)
	Vagus Nerve Stimulation not working properly, going to hospital for observation	0	0 (0.0%)	1	1 (9.1%)	1	1 (5.6%)
Respiratory	Increases number of seizures due to chest infection and antibiotics	0	0 (0.0%)	1	1 (9.1%)	1	1 (5.6%)
Other:	Gynae-pregnancy. Increase in the number of fits due to being pregnant / just given birth	0	0 (0.0%)	2	1 (9.1%)	2	1 (5.6%)
	Diagnosis of status epilepticus	1	1 (14.3%)	0	0 (0.0%)	1	1 (5.6%)
	Vertigo	0	0 (0.0%)	1	1 (9.1%)	1	1 (5.6%)
**Total**		6	6 (100.0%)	13	11 (100.0%)	19	18 (100.0%)

Notes:

[a] Eighteen (35.3%) patient participants reported adverse events (AE) over the course of follow-up. In the SAFE+TAU group, 6 AEs were reported by 6 participants, while 13 AEs were reported by 12 participants from the TAU group. Only one SAE occurred during the study – namely, a diagnosis of previous status epilepticus. This was reported by one SAFE+TAU group participant. A medical review asserted it unlikely to be related to participation as it arose from investigations the participant had been having before participation.

[b] Two participants were under investigation to determine whether a brain tumour could be the cause of their seizure changes; hence 'Body System' is classified as 'Neoplasia' rather than 'Neurological.'

Supplementary File 8 Total observed costs and cost per delegate to deliver the seizure first aid intervention (SAFE) within a research trial with two academic researchers, one Epilepsy Specialist Nurse delivering the training, and administrative support.

**Description**	**Design & set-up**	**Practice Session 1**	**Practice Session 2**	**SAFE+TAU Group 1**	**SAFE+TAU Group 2**	**SAFE+TAU Group 3**	**SAFE+TAU Group 4**	**SAFE+TAU Group 5**	**SAFE+TAU Group 6**	**SAFE+TAU Group 7**	**TAU Group 1**	**TAU Group 2**
Fixed costs (£)												
Equipment	928.85	0.00	0.00	0.00	0.00	0.00	0.00	0.00	0.00	0.00	0.00	0.00
Website	77.12	0.00	0.00	0.00	0.00	0.00	0.00	0.00	0.00	0.00	0.00	0.00
Freepost licence	116.40	0.00	0.00	0.00	0.00	0.00	0.00	0.00	0.00	0.00	0.00	0.00
Venue	0.00	80.00	80.00	80.00	80.00	80.00	80.00	80.00	80.00	80.00	80.00	80.00
Facilitator (staff cost)		192.79	192.79	177.96	177.96	177.96	177.96	177.96	177.96	177.96	177.96	177.96
Facilitation resources		4.96	4.96	4.96	4.96	4.96	4.96	4.96	4.96	4.96	4.96	4.96
Total (fixed)	1122.37	277.75	277.75	262.92	262.92	262.92	262.92	262.92	262.92	262.92	262.92	262.92
Variable costs (£)												
Staff costs	4243.41	612.88	1158.56	591.19	521.84	429.37	429.37	318.41	318.41	318.41	299.96	416.03
Staff travel expenses	44.10	279.87	279.87	274.67	274.67	265.43	265.43	265.43	265.43	265.43	233.63	265.41
Participant expenses	0.00	180.00	200.00	40.00	40.00	40.00	40.00	40.00	40.00	40.00	92.80	0.00
Office costs (inc. telephone)	589.33	33.00	105.50	81.63	85.23	92.60	88.70	89.00	86.60	89.00	57.17	70.13
Stationery, print, post	0.00	156.35	196.96	57.44	93.68	68.93	68.93	34.46	22.98	45.95	182.73	298.44
Refreshments	0.00	72.25	72.25	41.80	41.80	32.81	32.00	41.00	26.60	18.50	21.00	34.50
Advertising	45.00	0.00	0.00	0.00	0.00	0.00	0.00	0.00	0.00	0.00	0.00	0.00
Total (variable)	4921.84	1334.34	2013.13	1086.73	1057.22	929.13	924.42	788.30	760.01	777.29	887.28	1084.51
TOTAL COST (£)	6044.21	1612.09	2290.88	1349.65	1320.14	1192.05	1187.34	1051.22	1022.93	1040.21	1150.20	1347.43
Delegate (n) [patient:SO]		12	15	~~2:~~3:2	4:4	3:3	4:2	3:0	1:1	2:2	4:4	8:5

Mean cost/delegate (£)		134.34	152.73	269.93	165.02	198.68	197.89	350.41	511.47	260.05	143.78	103.65
Mean cost per group (sd)<br>95% central range *				£1,166 (134.22)<br>£1,023-£1,350							£1,249 (139.46)<br>£1,150-£1,347	
Mean cost per delegate<br>95% central range*				£240<br>£211-£278							£119<br>£110-£128	
Mean cost per patient (with or without SO)<br>95% central range*				£408<br>£358-£472							£208<br>£192-£225	

Notes:

*Central ranges generated from 10,000 bootstrap replications; The quantity of use of the resources, was measured. To capture the resources used the 'time and motion method' was used with AN, DS, JB and GM completing electronic data collection forms on which they self-reported the time they dedicated to different activities. The research team (AN, DS) also recorded details of the number of participants invited and attending each SAFE sessions and non-staff resources.

Supplemental File 9 Serious Adverse Event Protocol

Adapted from the Medicines for Human Use (Clinical Trials) Regulations 2004 (SI 2004/1031), http://www.legislation.gov.uk/uksi/2004/1031/pdfs/uksi_20041031_en.pdf [accessed 29th August, 2019] a serious adverse event (SAE) was defined as an adverse event which resulted in any of the following:

- Death
- Was life-threatening (subject at immediate risk of death, e.g., status epilepticus)
- Seizure resulting in hospital admission for ≥24 hours
- Emergency attendance or hospital admission for reason other than seizure
- Results in persistent or significant disability or incapacity, or
- Was otherwise considered medically significant.

'Life-threatening' in the definition of 'serious' referred to an event in which the patient was at risk of death at the time of the event; it did not refer to an event which hypothetically might have caused death if it were more severe. Hospitalisations for a pre-existing condition, including elective procedures that had not worsened, did not constitute an SAE. Prolongation of hospital stay due to social factors, for example, geographical location of the participant's home which prevented discharge was also not considered a SAE.

Given the characteristics of the subject population being studied, the following events were expected in this study population and were not recorded as part of the SAE monitoring process:

- Epileptic seizures with or without injury;
- Emergency or urgent medical attention. This includes visiting a hospital emergency department with the duration of the stay lasting <24 hours, attending an NHS out of hours primary care service (NHS Urgent Care 24), telephoning for an ambulance, telephoning NHS 111, seeking/ having an urgent/ fast-tracked appointment with a usual care provider (GP or specialist) or other registered health professional (e.g., a pharmacist);
- Side-effects of AED medication;
- Diagnosis of a comorbid psychiatric condition.

A delegated medically qualified person within the team (AM; LR) assessed each unexpected SAE. This person considered information on the temporal and physical relationship between the event and possible causes and assessed whether the event was related or unrelated to the patient's participation in the study. Further details on the process followed detailed below.

Monitoring

As part of this trial patient participants did not receive additional medical reviews. There is also no 'live' system which can be used to track SAEs such as emergency admissions and usual care providers are not systematically informed of them. Therefore, to monitor SAEs the research team liaised with patient participants themselves. A standardised form was to be completed at 3- (T1, by telephone), 6- (T2, by telephone) and 12-months (T3, during a face-to-face appointment) post-randomisation to collect information on patient participants' experience of unexpected, SAEs.

In each instance, a maximum of 3 attempts was made to contact the patient participant by telephone (including trying to contact them via their informal carer if they are taking part with one). If the patient was

not be contactable, a letter was be sent the patient's GP asking them to inform the research team if the patient was no longer alive and the circumstances of their death.

Causality

One of the delegated medically qualified persons within the team assessed each unexpected SAE. They considered information on the temporal and physical relationship between the event and possible causes and assessed whether the event was related or unrelated to the patient's participation in the study. In doing this, they used the definitions in the Table below.

Table Definitions of causality for serious adverse event

	**Description**
**Unrelated**	There is no evidence of any causal relationship. There is an alternative cause for the SAE.
**Unlikely**	There is little evidence to suggest there is a causal relationship (e.g. the event did not occur within a reasonable time after receipt of the intervention). There is another reasonable explanation for the event (e.g. the participant's clinical condition, other concomitant treatment).
**Possibly**	There is some evidence to suggest a causal relationship (e.g. because the event occurs within a reasonable time after receipt of the intervention). However, the influence of other factors may have contributed to the event (e.g. the participants clinical condition, other concomitant treatment).
**Probably**	There is evidence to suggest a causal relationship and the influence of other factors is unlikely.
**Almost certainly**	There is clear evidence to suggest a causal relationship and other possible contributing factors can be ruled out.

Reporting

As per National Research Ethics Service (2015) guidelines for non-CTIMPs, the main ethics committee approving the study and its Sponsor were informed within 15 days of the team becoming aware of any SAE that in the opinion of the medical reviewer was both unexpected (that is, the type of event is not listed in the protocol as an expected occurrence) and judged to be "possibly", "probably" or "almost certainly" related to participation in the study (that is, it resulted from administration of any of the research procedures, including the intervention).
