# Supplementary material for: Seizure First Aid Training For people with Epilepsy (SAFE) frequently attending emergency departments and their significant others: results of a UK multi-centre randomised controlled pilot trial
Source: BMJ Open. 2020 Apr 16;10(4):e035516. doi: 10.1136/bmjopen-2019-035516 (PMC7201300; doi:10.1136/bmjopen-2019-035516)
Supplement: Author's manuscript [file bmjopen-2019-035516.draft_revisions.pdf]

BMJ Open is committed to open peer review. As part of this commitment we make the peer review history of every article we publish publicly available.

When an article is published we post the peer reviewers' comments and the authors' responses online. We also post the versions of the paper that were used during peer review. These are the versions that the peer review comments apply to.

The versions of the paper that follow are the versions that were submitted during the peer review process. They are not the versions of record or the final published versions. They should not be cited or distributed as the published version of this manuscript.

BMJ Open is an open access journal and the full, final, typeset and author-corrected version of record of the manuscript is available on our site with no access controls, subscription charges or pay-per-view fees (http://bmjopen.bmj.com).

If you have any questions on BMJ Open's open peer review process please email
info.bmjopen@bmj.com

BMJ Open

Seizure First Aid Training For people with Epilepsy (SAFE) frequently attending emergency departments and their significant others: results of a UK multi-centre randomised controlled pilot trial

Journal:	*BMJ Open*
Manuscript ID	bmjopen-2019-035516
Article Type:	Original research
Date Submitted by the Author:	04-Nov-2019

Complete List of Authors:	Noble, Adam; University of Liverpool, Department of Health Services Research<br>Snape, Dee; University of Liverpool, Department of Health Services Research<br>Nevitt, Sarah; University of Liverpool, Department of Biostatistics<br>Holmes, EA; Centre for Health Economics & Medicines Evaluation<br>Morgan, Myfanwy; King's College London, Institute of Pharmaceutical Science<br>Tudur-Smith, Catrin; University of Liverpool, Biostatistics<br>Hughes, Dyfrig; Bangor University, Centre for Health Economics and Medicines Evaluation<br>Buchanan, Mark; Wirral University Teaching Hospital NHS Foundation Trust, Emergency Department, Arrowe Park Hospital<br>McVicar, Jane; Mackinnon Memorial Hospital, NHS Highland,<br>MacCallum, Elizabeth; Aintree University Hospital, Liverpool University Hospitals NHS Foundation Trust, Emergency Department<br>Goodacre, Steve; University of Sheffield, Medical Care Research Unit<br>Ridsdale, Leone; King's College London, Department of Basic and Clinical Neuroscience, Institute of Psychiatry, Psychology & Neuroscience<br>Marson, Anthony; University of Liverpool, Molecular and Clinical Pharmacology

Keywords:	Epilepsy < NEUROLOGY, ACCIDENT & EMERGENCY MEDICINE, Organisation of health services < HEALTH SERVICES ADMINISTRATION & MANAGEMENT, Health economics < HEALTH SERVICES ADMINISTRATION & MANAGEMENT, Clinical trials < THERAPEUTICS

SCHOLARONE™
Manuscripts

I, the Submitting Author has the right to grant and does grant on behalf of all authors of the Work (as defined in the below author licence), an exclusive licence and/or a non-exclusive licence for contributions from authors who are: i) UK Crown employees; ii) where BMJ has agreed a CC-BY licence shall apply, and/or iii) in accordance with the terms applicable for US Federal Government officers or employees acting as part of their official duties; on a worldwide, perpetual, irrevocable, royalty-free basis to BMJ Publishing Group Ltd ("BMJ") its licensees and where the relevant Journal is co-owned by BMJ to the co-owners of the Journal, to publish the Work in this journal and any other BMJ products and to exploit all rights, as set out in our licence.

The Submitting Author accepts and understands that any supply made under these terms is made by BMJ to the Submitting Author unless you are acting as an employee on behalf of your employer or a postgraduate student of an affiliated institution which is paying any applicable article publishing charge ("APC") for Open Access articles. Where the Submitting Author wishes to make the Work available on an Open Access basis (and intends to pay the relevant APC), the terms of reuse of such Open Access shall be governed by a Creative Commons licence – details of these licences and which Creative Commons licence will apply to this Work are set out in our licence referred to above.

Other than as permitted in any relevant BMJ Author's Self Archiving Policies, I confirm this Work has not been accepted for publication elsewhere, is not being considered for publication elsewhere and does not duplicate material already published. I confirm all authors consent to publication of this Work and authorise the granting of this licence.

For peer review only

TITLE: Seizure First Aid Training For people with Epilepsy (SAFE) frequently attending emergency departments and their significant others: results of a UK multi-centre randomised controlled pilot trial

AUTHORS: A.J. Noble,[1*] D Snape[1], S Nevitt,[2] EAF Holmes,[3] M Morgan,[4] C Tudur Smith,[2] D Hughes,[3] M Buchanan,[5] J McVicar,[6] E MacCallum,[7] S Goodacre,[8] L Ridsdale, [9] T Marson.[10]

AFFILIATIONS:

[1] Department of Health Services Research, University of Liverpool, Liverpool, UK

[2] Liverpool Clinical Trials Centre, Department of Biostatistics, University of Liverpool, Liverpool, UK

[3] Centre for Health Economics and Medicine Evaluation, Bangor University, Bangor, UK

[4] Institute of Pharmaceutical Science, King's College London, London, UK

[5] Emergency Department, Arrowe Park Hospital, Wirral University Teaching Hospital NHS Foundation Trust, Wirral, UK.

[6] Mackinnon Memorial Hospital, Broadford, NHS Highland, Isle of Skye, UK.

[7] Emergency Department, Aintree University Hospital, Liverpool University Hospitals NHS Foundation Trust

[8] School of Health and Related Research, Sheffield University, Sheffield, UK

[9] Department of Basic and Clinical Neuroscience, King's College London, London, UK

[10] Department of Molecular and Clinical Pharmacology, University of Liverpool, Liverpool, UK

FOR CORRESPONDENCE: Name: Adam Noble PhD; Address: University of Liverpool, Department of Health Services Research, Waterhouse Building, Liverpool, L69 3GB, United Kingdom.; Telephone: +44 151 794 5993; E-mail: adam.noble@liverpool.ac.uk

For peer review only

For peer review only - http://bmjopen.bmj.com/site/about/guidelines.xhtml

ABSTRACT

Objective: To determine the feasibility and optimal design of a randomised controlled trial (RCT) of Seizure First Aid Training For Epilepsy (SAFE).

Design: Pilot RCT with embedded microcosting.

Setting: Three English hospital emergency departments (EDs).

Participants: Patients aged ≥16 with established epilepsy reporting ≥2 ED visits in the prior 12 months and their significant others (SO).

Interventions: Patients (and their SO) were randomly allocated (1:1) to SAFE plus Treatment-As-Usual (TAU) or TAU alone. SAFE is a 4-hour group course.

Main outcome measures: Two criteria evaluated a definitive RCT's feasibility: i) ≥20% of eligible patients needed to be consented into the pilot; ii) routine data on use of ED over the 12-months post-randomisation needed securing for ≥75%. Other measures included: eligibility; ease of obtaining routine data; availability of self-report ED data and comparability; SAFE's effect; and intervention cost.

Results: Of ED attendees with a suspected seizure, 424 (10.6%) were eligible; 53 (12.5%) patients and 38 SOs consented. Fifty-one patients (and 37 SOs) were randomised. Routine data on ED use at 12-months was secured for 94.1%. Self-report ED data was available for 66.7%. Patients reported more visits compared to routine data. Most (76.9%) randomised to SAFE received it and no related serious adverse events occurred. ED use at 12 months was lower in the SAFE+TAU arm compared to TAU alone, but not significantly (Rate Ratio= 0.62, 95% confidence interval 0.33 to 1.17). A definitive trial would need ~674 patient participants and ~39 recruitment sites. Obtaining routine data was challenging, taking ~8.5 months.

Conclusions: In satisfying only one predetermined 'stop/go' criterion, a definitive RCT is not feasible. The low consent rate in the pilot raises concerns about a definitive trial's findings external validity and means it would be expensive to conduct. Research is required into how to optimise recruitment from the target population.

Trial registration number: ISRCTN13 871 327.

For peer review only - http://bmjopen.bmj.com/site/about/guidelines.xhtml

'Strengths and limitations of this study' (5 bullet points; one sentence each, relating to the methods)

1. Randomisation was done remotely by computer and stratification factors and allocations concealed from those collecting baseline and follow-up data and analysing it.
2. Participants were recruited from sites serving areas where social deprivation was high and epilepsy control poor and so similar to those where a definitive RCT would likely need to focus recruitment.
3. We completed one of the few microcostings of a self-management intervention for epilepsy.
4. Despite recruited patient participants stating sufficient ED use when screened, routine data subsequently suggested ~40% did not meet the inclusion criteria.
5. We estimated the effect of SAFE only on the proposed primary outcome measure for a definitive RCT.

KEY WORDS: Epilepsy; Accident & Emergency Medicine; Organisation of health services; Heath economics; Clinical Trials.

Words: 3,955

.

For peer review only

1. BACKGROUND

International evidence shows a significant minority of people with epilepsy (PWE) frequently use hospital emergency departments (EDs).[1-4] In the United Kingdom (UK), around 20% of PWE visit each year, half are admitted [5-7]. Whilst the exact distribution of use amongst attendees is unclear,[8-10] ~60% may make several visits each year.[11]

Emergency hospital use by PWE has been identified as an area for potential cost savings [12] as whilst expensive – ~£70-90M in England each year [8 13 14] – it is often of little clinical benefit since most attendees have known, rather than new epilepsy and experienced uncomplicated seizures.[9 15-17] Guidance states such seizures could be managed by PWE and their significant others (SOs).[18 19] Indeed, iatrogenic harms may arise in seeking emergency care for them.

PWE visiting EDs have a characteristic profile. They report more seizures, anxiety, poorer quality of life and are more likely to live in socially deprived areas compared to the wider epilepsy population.[11 20-23] They therefore share some of the characteristics of those at increased risk of epilepsy associated death.[24 25] In the UK at least, PWE visiting EDs can though be challenging to identify for research since most (~62%) are not being followed-up by an epilepsy specialist,[1] primary care providers are also not always notified of ED visits by their patients [9] and because EDs do not always code the reason for an attendance.[26]

Whilst the focus on PWE attending EDs is welcomed, it needs to be translated into care improvements. Although a range of promising interventions have been suggested,[27] assessment of their utility is lacking.[28]

One intervention proposed is seizure first aid training. It has potential as PWE frequently visiting EDs and their SOs (to whom care decisions can be delegated [29 30]) express particularly low knowledge and confidence in this domain, are fearful of seizures, and there are indications this leads them to seek emergency medical attention for seizures, rather than self-managing them.[31]

As no such intervention existed, we developed one – called Seizure First Aid Training For Epilepsy (SAFE). SAFE is a manualised 4-hour group-based self-management

For peer review only - http://bmjopen.bmj.com/site/about/guidelines.xhtml

course (Table 1). SAFE's rationale and development has been reported.[32] It's efficacy now needs testing. A randomized controlled trial (RCT) would be an appropriate methodology for this. ED use over the 12 months following randomisation could be the primary outcome. It is unknown though whether such a trial is feasible and what its optimal design should be.

Specifically, will patients and their SOs take part, attend SAFE, and be willing and able to be followed-up? It is also not possible to calculate the required sample size because the distribution of ED use is unclear. These uncertainties exist because SAFE is newly developed and no RCT has attempted to recruit the target population.

It is also unclear how to measure ED visits. Funders are encouraging trialists to use routinely recorded data to assess outcomes. In England, routine data on a person's ED use is recorded within the Hospital Episode Statistics system. This data's cost, how long it takes to obtain and its comparability to self-report is though unknown.
In such circumstances, guidelines highlight the importance of pilots.[33] Their primary focus is not effect, but judging feasibility and providing information to optimise a definitive RCT's design.[34] We thus completed a pilot RCT comparing SAFE, plus treatment-As-Usual (TAU) to TAU alone. It had the following objectives, which will be relevant to those interested in SAFE and those researching this population:

i) Estimate likely eligibility, consent, and retention rates in a definitive RCT;
ii) Estimate annual rate of ED visits in TAU group and the likely dispersion parameter;
iii) Determine feasibility of measuring ED use by routine data;
iv) Estimate completion rates of study assessment tools;
v) Estimate rates of researcher unblinding;
vi) Provide summary statistics to estimate effect of SAFE on ED use and its precision;
vii) Capture patient and SOs views on trial participation;
viii) Estimate the intervention's cost.

2. METHODS

For peer review only - http://bmjopen.bmj.com/site/about/guidelines.xhtml

The trial's protocol has been published.[31] Here we provide a brief overview.

2.1 Design

Parallel arm, multicentre, external pilot RCT. Assessments with participants on the definitive trial's proposed primary and secondary outcome measures were performed at baseline prior to randomization (T0) and 12-months later (T3) (Table 2). Interim assessments occurred at 3 (T1) and 6-months (T2).

SAFE was offered to the TAU alone group after T3 assessments were completed.

2.2 Trial setting

Three hospitals in North-West England – which serve populations featuring high social deprivation [35 36] and emergency admissions for epilepsy [22] – were recruitment sites (Acknowledgements). From May to December 2016 patients were invited who had attended any of these hospital's ED over the prior 12 (and with governance approval, later extended to 18) months for epilepsy (see 2.4).

2.3 Ethical considerations and approvals

The National Research Ethics Service Committee (15/NW/0225) and Health Research Authority (166241) approved the study. An independent Trial Steering Committee monitored it.

2.4 Patient recruitment

ED clinicians searched their hospitals' attendance record systems for potential participants (Supplementary File 1), screened their triage cards and posted invitations to eligible patients. Recipients had 3 weeks to return an opt-out response if not interested in participating. Those not opting-out were telephoned by a researcher to verify eligibility and willingness to participate. Patients taking part (and their SO if they chose to take part with one) provided informed consent at an enrolment appointment with a researcher (DS). For patients, this included consent to access their routine data.

2.5 Eligibility criteria

For peer review only - http://bmjopen.bmj.com/site/about/guidelines.xhtml

Table 3 details the criteria. In brief, patients were eligible if they were aged ≥16, diagnosed with epilepsy, prescribed antiepileptic drugs, could give informed consent and, when telephoned, self-reported ≥2 ED visits for epilepsy in the prior 12 months.

2.6 Randomization and blinding

Patients (and their SOs) were randomised (1:1) by an online system managed by the Liverpool Clinical Trials Centre. It used a minimisation program with a built-in random element and two stratification factors (recruitment site and whether the patient reported epilepsy stigma at baseline).

Usual care provider(s) and DS, who was responsible for recruitment and follow-up, were blinded to allocations and stratification factors. Participants could not be blinded and so were asked not to reveal their allocation to DS.

Staff (GM) organizing attendance at SAFE were not involved in data collection and not blinded. The trial (SN) statistician and senior statistician (CTS) were blinded until the database was ‘locked’.

2.7 Measures to assess patient and SOs participants’ outcomes

Table 2 details these measures.

2.8 Intervention

An epilepsy nurse specialist (JB) delivered SAFE within a local hospital’s educational centre. A fidelity assessment found they delivered SAFE with excellent protocol adherence and competence.[37]

2.9 Outcomes

To achieve the study objectives, rates of eligibility, consent and retention were calculated. Retention being the percentage of patients for which 12-month primary outcome data was secured. Two a priori progression criteria helped judge feasibility: i) ≥20% of eligible patients were recruited; ii) 12-month primary outcome data secured for ≥75% of patients.

For peer review only - http://bmjopen.bmj.com/site/about/guidelines.xhtml

By assessing participants on the proposed outcome measures, we obtained estimates of the distribution of ED use, measure completeness and SAFE's effect. To evaluate blinding, DS completed a 'Treatment Guess' form after each participant's T3 assessment or withdrawal. The proportion of participants willing to participate in such a trial again was ascertained and experience of adverse events calculated. Time taken to obtain routine data and the fee payable was recorded. The agreement between routine data and patient self-report was also explored.

2.10 Statistical analyses

As this was a pilot RCT, a power calculation was inappropriate. Instead, the sample size was chosen to provide adequate precision to estimate the parameters required.[38] With 80 patient participants we could estimate a potential drop-out rate of 25% to within a 95% confidence interval of +/-10% and a consent rate of 20% to within a 95% confidence interval of +/-4%. Assuming ED data at T3 was not available for 25% of patients, data from 60 patients would still allow robust estimation of the ED rate and dispersion parameter. Sample sizes of 24 to 50 have been recommended as 'adequate' for pilot studies.[38 39] Based on existing data,[1 14 17 40 41] it was anticipated 12 months of attendances from each ED would allow identification of ~400 eligible patients and 80 patient participant accruals.

Analyses were documented a priori in an analysis plan and performed using SAS (9.4). Baseline characteristics were calculated for patient and SO participants and patient participants compared, on a subset, to eligible patients declining participation. Parameters are reported with 95% confidence intervals (CIs).

The proportion of correct treatment guesses was determined and Cohen's Kappa calculated. Bland-Altman plots compared ED visits as measured by routine data and self-report.[42]

SAFE's effect on ED use, with and without adjustment for number of ED visits prior to randomisation, was estimated on an intention-to-treat basis with no data imputation, using negative binomial regression (NBR). Between-group differences are presented as Rate Ratios and tested according to 5, 10 and 20% levels of significance.[43]

For peer review only - http://bmjopen.bmj.com/site/about/guidelines.xhtml

As no consensus exists about what constitutes a clinically important reduction in ED visits,[44] average annual rate of ED visits in the SAFE+TAU and TAU alone groups post randomisation and the likely dispersion parameter from the adjusted NBR model were used to estimate the sample size of a definitive trial using Keene et al.'s [45] formula.

2.11 Microcosting

Microcosting adopted the perspective of an academic non-profit making institution and conducted in three steps:[46] (1) resource identification; (2) resource use measurement, applying the time and motion method;[47] and (3) valuation using price year 2017/18 for local and national data. Data were analysed using Microsoft Excel to calculate the fixed and variable costs of delivering SAFE, presented as: cost per training session, mean cost per participant, mean cost per patient; based on 11 groups/year and equipment life of one-year. The 95% central range (CR) for costs and differences were generated using 10,000 bootstrap replications.

2.12 Patient and Public Involvement statement

This research came about as improving education for patients and families on epilepsy is a top research priority.[48] To ensure SAFE was developed and tested in a way that met service users' needs: the 'Epilepsy Society' and patient and SO representatives from the 'Brain and Spine Foundation' helped develop recruitment materials; the pilot was overseen by a Trial Steering Committee including 2 service user representatives; SAFE's was designed by an Intervention Panel including 2 service user representatives and its content informed by feedback from 23 service users;[32] finally, pilot trial participants reported on the burden of participation with a view to optimising the design of a potential definitive RCT.

3. RESULTS

3.1 Participant recruitment, allocation and treatment

Of the 4016 individuals identified, 1220 (30.4%, CI 29.0% to 31.8%) had visited for established epilepsy (Figure 1). Of these, 424 (34.8%, CI 32.1% to 37.4%) were eligible; eligibility rate 10.6% (CI 9.6% to 11.5%) (Supplementary File 3 for reasons).
Of the 424 patients invited, 53 consented; consent rate 12.5% (CI 9.3% to 15.6%). Telephone contact could though only be made with 203 (47.8%, CI 43.1% to 52.6%) patients. The consent rate amongst those who could be contacted was 26.1% (CI

For peer review only - http://bmjopen.bmj.com/site/about/guidelines.xhtml

20.0% to 32.2%). The main reasons for 150 patients declining participation were “lack of interest” (42.7%, CI 34.8% to 50.6%) and being “too busy” (22.6%; CI 16.0% to 29.4%).

Of the 53 consenting patients, 51 were randomised (with 37 SOs) (Figure 1 gives reasons for non-randomisation). Of the 51, 26 (and 18 SOs) were allocated to SAFE+TAU and 25 (and 19 SOs) to TAU alone. Most (20, 76.9%) patients and SOs (13, 72.2%) randomized to SAFE+TAU attended a course.

3.2 Participant demographics and epilepsy characteristics

The patient participants mean age was 39.9 years (SD 15.7, range 16-71); 29 (56.9%) were female and most (74.4%) lived in areas high in deprivation (49% in the 10 percent most socially deprived areas in England) (Table 4).

Recruited patients were comparable in age and deprivation to those declining participation. Females were though over-represented (Supplementary File 4).

Recruited patients had an epilepsy diagnosis for a median of 17.3 years. Most (62.8%) reported ≥10 seizures in the previous year and having seen a neurologist (74.5%) (Table 4). Participants’ mean Quality of Life 31-P score was low at 48.3 (SD 17.3; range 17.1 to 79.5). Twenty-six (50.9%) had ‘probable’ clinical anxiety. Most (n=42, 82.3%) reported feeling stigmatised by epilepsy; 15 (29.4%) highly. The treatment groups were broadly similar in demographics and epilepsy characteristics.

SO participants were typically a partner/spouse (43.2%) and most (89.2%) had daily patient contact (Supplementary File 5). With a mean Zarit Caregiver burden score of 18.9 (SD=12.51), SOs typically reported ‘little or no burden’ [49] but anxiety was high [50]; 15 (40.5%) SOs had ‘probable’ clinical anxiety.

3.3 Participant retention

3.3.1 Proposed primary outcome measure:

Of the randomised patients, 3 (5.8%) formally withdrew over follow-up, removing access to their routine data. Primary outcome data on ED use at 12 months (and for the 12 months prior to randomisation) was available for the remaining 48 (94.1%).

For peer review only - http://bmjopen.bmj.com/site/about/guidelines.xhtml

3.3.2 Proposed secondary outcome measures:

Thirty-seven (72.5%) randomised patients and 21 (56.8%) SOs attended their 12-months questionnaire appointment (T3). The extent to which measures were completed at these and the interim appointments varied (Supplementary File 6). Self-report data on ED use at T3 was obtained from only 34 (66.7%) patients.

3.4 ED use

3.4.1 Baseline, prior to randomisation:

Routine data for the 48 patients for whom consent was maintained showed they made 122 ED visits in the 12 months before randomisation (Supplementary File 7). The mean was 2.5 and median 2 (range 0 to 12). ED use was slightly higher for TAU participants (Table 5).

Despite only consenting patients who when telephoned reported ≥2 ED visits in the prior 12 months, routine data indicated 4 (8.3%) had not made any visits during the prior 12 months. A further 19 (39.6%) made only one.

3.4.2 At 12 months, effect of SAFE:

Compared to the 12-months prior to randomisation, mean ED use over follow-up reduced for the SAFE+TAU group from 2.1 visits to 1.8 (difference -0.3). For the TAU group, it increased from 3 visits to 3.4 (difference 0.4) (Table 5). Unadjusted NBR estimated the visit rate was 46% lower in the SAFE+TAU group compared to the TAU group (Rate Ratio= 0.54; Vuong's test z=-0.17, P=0.87). In the adjusted model, SAFE's effect reduced (Rate Ratio= 0.62). Between group differences were not significant at the 5% or 10% alpha level in either the unadjusted or adjusted model. The effect was significant in both at the 20% level (Table 6). The dispersion parameter under the adjusted NBR model was k=0.69 (CI 0.17 to 1.21).

3.5 Obtaining routine data and its correspondence with self-report

Routine data took 8.5 months to secure, arriving ~9 months after T3 assessments finished. The direct cost was £6960. Supplementary File 8 shows substantial work on behalf of the research team to obtain the data was required. In one instance, an appeal against a decision to reject the application – 5 months in – was necessary.

For peer review only - http://bmjopen.bmj.com/site/about/guidelines.xhtml

Routine data did not match patient self-report in most cases. Forty-two patients had self-report and routine data on ED use at baseline. Only 3 (7.1%) reported the same number of visits as their routine data indicated. Most (76.2%) reported more (by 3.8 visits on average) (Figure 2a). There was greater agreement between routine and self-report data at T3; 11 (32.4%) patients reported the same number of visits as their routine data (Figure 2b).

3.6 Blinding

The researcher correctly guessed the allocation of 35 patient participants; unblinding rate 68.6% (CI 54.1% to 80.9%). The chance-corrected kappa statistic of 0.37 (CI 0.12 to 0.63) equated to "fair" agreement.

3.7 Safety

No serious adverse events related to participation occurred (Supplementary File 9).

3.8 Participant feedback

Thirty-two (68.1%) patients and 20 (62.5%) SOs completed the T3 feedback questionnaire. All but one said they would participate in such a trial again; participants indicated they perceived benefits from SAFE.

3.9 Sample size calculation

Keene et al.'s formula indicated a definitive RCT would require a sample of 674. This was with alpha set at 1%, 90% power, and accounted for the 9% of patients that could withdraw.

3.10 Microcosting

Delivering SAFE was estimated to cost £333 (CR £288 to £380) per patient (with or without a SO). When including the cost SAFE's development (£181 per person), the mean cost per *attendee*, based on all 55 participants in SAFE+TAU or TAU groups who ultimately attended a SAFE session, was £375 (CR £348 to £402) (Supplementary File 10). This reduced to £214 (CR £188 to £241) when excluding sunk costs. Staff time accounted for 50.01% of the cost of SAFE's delivery. SAFE's facilitator was not local to the trial area. Thus, an analysis of facilitator costs without

For peer review only - http://bmjopen.bmj.com/site/about/guidelines.xhtml

travel expenses (time and cost), reduced the mean cost by 21.08% from £194 (CR £167 to £221) to £152 (CR £124 to £179) per delegate (£261 per patient with or without SO). The annual fixed cost of setting-up and running SAFE was £1122 (+£35.07 per patient, based on 32 patients/year attending with or without SO).

4. DISCUSSION

The pilot was successful, providing estimates of key parameters, including recruitment and retention. An informed decision regarding feasibility and optimal design of a definitive trial of SAFE can now be made.

4.1 Positives from pilot for feasibility and design of definitive trial

4.1.1 Identification, treatment and safety:

The pilot indicates it is possible to identify, consent, randomise and safely treat persons from the target population within a definitive trial. This was not a given since no RCT had focused recruitment on them.

4.1.2 Retention and measuring ED use:

By using routine data as the basis of the primary outcome measure, a definitive trial should not be affected by attrition. It permitted 12-month data to be secured for 94% of pilot participants. Despite having shorter-follow-ups (6 months), 151 definitive RCTs funded by the NIHR secured outcome data for 89% of patients.[51] The use of routine data in our pilot meant it satisfied the retention progression criterion. It would not have been met if self-report data were relied on (only 67% of patients provided it). This leads us to recommend ED use be measured using routine data.

Another reason for the recommendation is its cost would be low compared to employing staff to obtain self-report data. Routine data would also prevent a definitive trial from exposure to apparent recall bias, with patients seemingly over reporting ED use (it has been asserted bias is not an issue for ‘‘big ticket’’ service items).[52 53]

Our pilot does though caution that provision of routine data is slow and not straightforward. It took 8.5 months to obtain. In principle, such applications should be processed within 2 months.[54] Data providers should attend to such issues as those funding and designing trials need confidence data can be secured and realistic

For peer review only - http://bmjopen.bmj.com/site/about/guidelines.xhtml

estimates for when. It is also worth noting that some of the ED visits attributed to our participants may not have been epilepsy related. Since a diagnostic code is not recorded for ~35% of ED visits, we asked NHS Digital to inform us of any ED visits recorded for our participants.[26]

4.2 Negatives from pilot for feasibility and design of definitive trial

4.2.1 Consent:

Only a small proportion of identified patients were eligible (10.6%) and willing (12.5%) to take part, meaning the progression criterion that ≥20% of eligible patients agree to participate was not met.

The progression criterion was stated a priori because when uptake is so low there is the real possibility that those who are and are not taking part differ. We compared the age and deprivation of patients who did and did not take part and found no obvious differences. We do not know though whether they differed on other indices since access to non-participants' medical records was not ethically permissible. One indication the pilot might not have recruited a representative sample was the high proportion of patients seeing a neurologist in the prior 12 months (~75%). Evidence suggests this should be closer to 38%.[1]

The consent rate also raises questions over the likelihood of a definitive trial being funded since it would make it expensive. On average, each pilot site generated 17.6 patient accruals from 18 months of attendances. To achieve a sample size of ~674 PWE, a definitive trial could thus require ~39 sites (half of England's EDs). This is 2.5 times more sites than in recent NIHR HTA funded RCTs [51] which already had a mean cost of ~£1.3M.[55] A definitive RCT of SAFE might thus not represent acceptable value for money to funders.[56]

Epilepsy and its consequences (e.g., seizures, memory difficulties, no driving license) makes recruiting PWE challenging. The consent rate in the pilot is nevertheless low. In the largest RCT of self-management (SMILE trial), 37% of the people with uncontrolled epilepsy invited took part.[57] The characteristics of the patients from ED agreeing to participate in our pilot suggests one reason for the low uptake might be stigma; 82% felt some (21.6% felt highly stigmatised). This is higher than in the wider

For peer review only - http://bmjopen.bmj.com/site/about/guidelines.xhtml

epilepsy population; 63% of SMILE's sample felt stigmatised (12.5% highly).[58] Stigma can make PWE feel ashamed and reluctant to talk about epilepsy. This could explain why the target population is so challenging to recruit. Unfortunately, it is unclear how to revise recruitment to mitigate against this.

Evidence-based strategies were employed in the pilot to maximise recruitment,[31] and invitation materials co-produced with patients. It is generally considered preferable though for a usual care provider, with whom a patient has an established relationship, to invite a person into research. Difficulties identifying the target population (see 1.0) meant we had local ED clinicians do the inviting. A future trial might consider asking EDs to identify ostensibly eligible patients, but for the GPs of the identified patients to do the inviting. This may boost recruitment.[59]

4.2.2 Effect of intervention:

Our pilot estimated SAFE's effect to be modest (reducing ED visits from 2.1 over 12-months to 1.8). This has negative consequences for a definitive trial, not least because it requires it to have a large sample to detect the effect. Efficacy was not though our pilot's primary focus and the estimate may be imprecise. Previous evidence does though suggest it is in the region expected.

Specifically, only one RCT – by Sajatovic et al.[60] – has considered change in ED use following epilepsy self-management.[61] Conducted in the U.S. it compared SMART – an 8-session group intervention – to wait-list control. No significant change between groups was found in subsequent ED/ hospitalization use. For SMART, it reduced from a mean of 1.22 by .44 over the 6 months following randomisation. For the controls it reduced from 2.4 by -1.26 visits.

4.3 Judgement regarding progression to definitive trial

Thabane et al.[62] provide a framework for judging whether to progress to a definitive trial. In satisfying only one progression criteria, a definitive trial based on the pilot's design is not feasible. We have also not identified any modifications that will make it feasible. We therefore recommend not proceeding.

For peer review only - http://bmjopen.bmj.com/site/about/guidelines.xhtml

4.4 Strengths and limitations

Strengths include the pilot being reported according to guidelines,[63] allocations being concealed, that patients were recruited from sites similar to those likely for a definitive trial and we included a microcosting of SAFE's delivery.

The pilot is not without potential weaknesses. Most importantly, despite recruited patient participants stating sufficient ED use when screened, routine data subsequently suggested ~40% did not meet the inclusion criteria. This could limit our findings external validity and attenuate SAFE's effect. Another potential limitation is we estimated the effect of SAFE only on the proposed primary measure. We did not estimate its wider effects, including on duration of ED visits and admissions.

5. CONCLUSION

A definitive trial of SAFE is not currently feasible. Research is required to determine how people from the target population can be better recruited.

For peer review only

For peer review only - http://bmjopen.bmj.com/site/about/guidelines.xhtml

ACKNOWLEDGEMENTS: We thank the people who kindly participated in this study and the local ED sites for their support – namely, Arrowe Park Hospital (Wirral University Teaching Hospital NHS Foundation Trust), and Aintree University Hospital and the Royal Liverpool University Hospitals (Liverpool University Hospitals NHS Foundation Trust). Other persons who contributed to this work are: Mrs. Juliet Bransgrove [JB] (epilepsy nurse specialist), Dr Duncan Appelbe (website development/ management) and Ms. Gail Moors [GM] (Administrator). We also acknowledge the support we received from our Steering Committee (Prof. Alasdair Gray [chair], Prof. Pete Bower, Dr. Paul Cooper, Mr. Mike Jackson, Ms. Helen Coyle, Mr. Mike Perry, Mrs. Linda Perry, Mrs. Jayne Burton and Mr. Sam Burton). The study sponsor was the University of Liverpool (reference: UoL001108) and the Clinical Trials Research Centre's registration number is 12.

AUTHORS CONTRIBUTIONS: AJN and LR conceived of the study and designed it together with AGM, CTS, DH, MM and SG. SJN and CTS planned and conducted the statistical analysis, EH and DH the economic analysis. AJN was the chief investigator, with MB, JM and EM leading participant identification and DS recruitment and follow-up. AJN wrote the manuscript, with revisions and approval of the final manuscript coming from all authors.

FUNDING STATEMENT: This project was funded by the National Institute for Health Research's Health Services and Delivery Research Programme (HS&DR Programme) (project number 14/19/09). The views and opinions expressed herein are those of the authors and do not necessarily reflect those of the University of Liverpool, the HS&DR programme, the NIHR, the NHS, or the Department of Health and Social Care.

COMPETING INTERESTS: None declared.

Competing interests: None declared.

PATIENT CONSENT FOR PUBLICATION: Not required.

DATA STATEMENT: Requests for anonymised data should be submitted to the corresponding author.

For peer review only - http://bmjopen.bmj.com/site/about/guidelines.xhtml

Table 1 SAFE course and its content

Overview

SAFE was developed to be delivered to groups of ~10 patient-SO dyads by a single facilitator with epilepsy knowledge who follows a treatment manual. Materials include standardised presentation slides, videos and interactive tasks. Patients are given information packs that include copies of the slides, wallet sized first aid instructions cards, paper epilepsy ID Cards and instructions for setting up IDs on smart-phone, and access to a website with the intervention's content on.

SAFE's behaviour change potential is supported by reference to Self-Affirmation Theory.

SAFE's order of presentation and content is as follows:

Order	Topics	Learning activity	Minutes allotted
1	Welcome	Slide	5
2	Goals of this course	Slide	2
3	What would you like from today?	Interactive	20
4	True or false?	Interactive	12
5	Taking on information (Kindness Questionnaire)	Interactive	10
6	Epilepsy, seizures & how the brain works	Video	10
7	First aid for convulsive seizures	Interactive	10
8	What can you do to help someone during a seizure?	Slide	5
9	What not to do during a seizure	Slide	5
10	What to do after the seizure has stopped	Slide	5
11	Questions or comments?	Interactive	10
12	Post-seizure states	Slide	15
13	Injuries	Slide	2
14	When to call an ambulance?	Slide	10
15	Questions or comments?	Slide	10
16	Refreshment break	Networking	10
17	Recovery position	Slide	2
18	Recovery position	Video	2
19	Let's practice the recovery position	Interactive	15
20	Questions or comments?	Interactive	5
21	Who needs to know how to help?	Interactive	5
22	What they need to know & why	Slide	5

For peer review only - http://bmjopen.bmj.com/site/about/guidelines.xhtml

23	How to get this information to them. Family, friends & work colleagues:	Slide	5
24	How to get this information to them. Members of the public and health workers:	Slide	5
25	Questions or comments?	Interactive	5
26	Refreshment break	Networking	5
27	Personal stories - introduction	Slide	2
28	Ben's story (case vignette)	Slide	6
29	How to change what happened to Ben? (Carrying medical ID; triggers)	Interactive	5
30	Triggers	Slide	5
31	Knowing your triggers	Slide	4
32	Some ways of dealing with triggers	Slide	4
33	Sandra's story (case vignette)	Slide	6
34	How to change what happened to Sandra (Warning signs; home safety)	Interactive	2
35	Main points to remember, If you have epilepsy:	Slide	3
36	Main points to remember; If you know someone with epilepsy:	Slide	2
37	Sources of further information	Slide	2
38	What's on the back table and accessing the study website*	Slide	2
39	Questions or comments?	Slide	2
40	Evaluation		-
41	Certificates of attendance		-
**Time in minutes:** 240			

Notes:

* http://www.seizurefirstaid.org.uk/Intervention/

For peer review only

Table 2 Proposed primary and secondary outcome measures for a definitive trial used within pilot by assessment and participant type [a]

Outcome	Domain	Participants	Measure and derivation of outcome	Baseline (T0)	3-month (T1)	6-month (T2)	12 month (T3)
Primary	ED visits	PWE	*Routine data, Hospital Episode Statistics Accident & Emergency system* NHS numbers of patient participants securely transferred to NHS Digital who hold the Hospital Episode Statistics system. NHS Digital then provided the number of ED visits these patients were recorded as having made in the 12 months prior to and in the 12 months following their randomisation. [b]	✓	-	-	✓
Secondary	ED visits	PWE	*Self-report, "How many times have you used Casualty / Accident & Emergency (A&E) over the past 12 months for epilepsy?"* (For T2 the time frame was 6 months) Provided with seizure diary at T0 to assist.	✓	-	✓	✓
	Seizure control	PWE	Baseline: *Thapar's*[64] *Seizure Frequency Scale* for the prior 12 months. T2 and T3: Asked for number of epileptic seizures (of any type) since last assessment and dates of the first and most recent. Provided with seizure diary at T0 to assist.	✓	-	✓	✓
	Quality of life	PWE	*Quality of Life in Epilepsy Scale-31-P (31 items)* [65]	✓	-	✓	✓
	Distress	PWE; SOs	*Hospital Anxiety and Depression Scale (14 items)* [66] [c]	✓	-	-	✓
	Felt Stigma	PWE; SOs	*Stigma of Epilepsy Scale (9 items)* [67] [d]	✓	-	-	✓
	Burden	SOs	*Zarit Caregiver Burden Inventory (22 items)* [49] [e]	✓	-	✓	✓
	Adverse events	PWE	*Monitored by PWE completing a checklist.* [f]	-	✓	✓	✓
	Fear of seizures	PWE; SOs	*Epilepsy Knowledge and Management Questionnaire – Fears subscale (5 items)* [68]	✓	-	-	✓

For peer review only - http://bmjopen.bmj.com/site/about/guidelines.xhtml

	Knowledge of what to do when faced with a seizure	PWE; SOs	*Thinking About Epilepsy Questionnaire (13 items)*[69]	✓	-	-	✓
	Confidence managing seizures/ epilepsy	PWE; SOs	PWE: *Epilepsy Mastery Scale (6 items)* [70]<br>SOs: *Parents Response to Child Illness Scale- Condition Management subscale* (6 items) [71]	✓	-	✓	✓
	Health economics	PWE	*Client Service Receipt Inventory* [72] and *EQ-5D* (13 items) [73]	✓	-	-	✓
-	Feedback on trial participation	PWE; SOs	Adapted from Magpie Trial (3 items) [74]<br>(1) "If time suddenly went backward, and you had to do it all over again, would you agree to participate in the Seizure First Aid Training trial?";<br>(2) "Please tell us if there was anything about the Seizure First Aid Training Trial that you think could have been done better";<br>(3) "Please tell us if there was anything about the Seizure First Aid Training Trial, or your experience of joining the trial, that you think was particularly good"	-	-	-	✓

Notes:

ED= Emergency Department; PWE= Person with epilepsy; SOs= Significant others.

[a] Unless a patient formally withdrew consent to participate in the trial, routine data on their ED use was requested and up to 3 attempts were made to contact patients or SO participant each time one of their follow-up assessments was due.

[b] To minimise cost, a single application for routine data was submitted, rather than one relating to the baseline period and one for follow-up.

[c] Total anxiety and total depression scores were categorised according to the following: 0 to 7, 'Normal range'; 8 to 10, 'Suggestive of anxiety / depression'; 11 to 21, 'Probable anxiety / depression.'

[d] Total stigma scores were categorised as follows: 0, 'No stigma'; 1 to 6, 'Mildly to moderately stigmatized'; 7 to 9, 'Highly stigmatized;

[e] Total Zarit Burden scores were categorised as follows: 0 to 20, 'Little or no burden', 21 to 40, 'Mild to moderate burden', 41 to 60, 'Moderate to severe burden'; 61 to 88, 'Severe burden.'

[f] Definitions of serious adverse event and how relatedness was assessed is presented in Supplementary File 2.

For peer review only

Table 3 Participant inclusion and exclusion criteria

Participants	Inclusion criteria	Exclusion criteria
Patients	• Established diagnosis of epilepsy (≥1 year);<br>• All epilepsy syndromes and all types of focal and generalised seizures;<br>• Currently being prescribed antiepileptic medication;<br>• Age ≥16 years (no upper age limit);<br>• Visited an ED for epilepsy on ≥2 occasions within the previous 12 months (as reported by patient);<br>• Live within 25 miles of any of the ED recruitment sites;<br>• Able to provide informed consent, participate in intervention and independently complete questionnaires in English.	• Actual or suspected psychogenic non-epileptic seizures alone or in combination with epilepsy;<br>• Acute symptomatic seizures related to acute neurological illness or substance misuse;<br>• Severe current psychiatric disorders (e.g. acute psychosis) or life-threatening medical illness;<br>• Enrolled in other epilepsy related non-pharmacological treatment studies.
Carers	• A significant other to the patient (e.g., family member, friend) who the patient identifies as providing informal support;<br>• Age ≥16 years (no upper age limit);<br>• Lives in the North West area of England	• Severe current psychiatric disorders or life threatening medical illness;<br>• Enrolled in other epilepsy related non-pharmacological treatment studies.

Notes:

Patient participants were permitted to take part without a carer. Carers could not, however, take part without a patient partner having at least consented to take part into the study.

For peer review only

Table 4 Baseline demographic, disease characteristics and service use of patient participants

Variable		SAFE + TAU	TAU	Total
Sex: n (%)		(n=26)	(n=25)	(n=51)
Male		10 (38.5%)	12 (48.0%)	22 (43.1%)
Female		16 (61.5%)	13 (52.0%)	29 (56.9%)
Missing		0 (0.0%)	0 (0.0%)	0 (0.0%)
Age at presentation to the emergency department (years)				
N		26 (100.0%)	25 (100.0%)	51 (100.0%)
Mean		39.2	40.7	39.9
Standard deviation		13.96	17.52	15.66
Minimum		18.9	16.5	16.4
Median		37.1	41.4	38.8
Maximum		69.9	71.3	71.3
Missing		0 (0.0%)	0 (0.0%)	0 (0.0%)
Index of Multiple Deprivation (IMD) [a]		(n=26)	(n=25)	(n=51)
IMD Decile: 1	n (%)	11 (42.3%)	14 (56.0%)	25 (49.0%)
IMD Rank	Minimum	44	48	44
	Median	574.0	1231.5	673.0
	Maximum	3202	2166	3202
IMD Decile: 2 to 3	n (%)	7 (26.9%)	6 (24.0)	13 (25.4%)
IMD Rank	Minimum	4649	3989	3989
	Median	6785	6665	6785
	Maximum	8281	7816	8281
IMD Decile: 4 to 6	n (%)	2 (7.7%)	4 (16.0%)	6 (11.8%)
IMD Rank	Minimum	9881	11480	9881
	Median	12836	11924	11924
	Maximum	15791	16004	16004
IMD Decile: 7 to 10	n (%)	5 (19.2%)	1 (4.0%)	6 (11.8%)
IMD Rank	Minimum	24971	32724	24971
	Median	27642	32724	28876
	Maximum	31002	32724	32724
IMD Decile missing [c]	n (%)	1 (3.8%)	0 (0.0%)	1 (2.0%)
Ethnicity: n (%)		(n=26)	(n=25)	(n=51)
White		25 (96.2%)	23 (92.0%)	48 (94.1%)
Asian / Asian British		0 (0.0%)	0 (0.0%)	0 (0.0%)
Black / African / Caribbean / Black British		1 (3.8%)	1 (4.0%)	2 (3.9%)
Mixed / multiple ethnic groups		0 (0.0%)	0 (0.0%)	0 (0.0%)
Other ethnic group		0 (0.0%)	1 (4.0%)	1 (2.0%)
Missing		0 (0.0%)	0 (0.0%)	0 (0.0%)
Other significant medical history: n (%)		(n=26)	(n=25)	(n=51)
No, none		13 (50.0%)	10 (40.0%)	23 (45.1%)
Yes, another medical condition/s		10 (38.5%)	13 (52.0%)	23 (45.1%)
Yes, a psychiatric condition		1 (3.8%)	0 (0.0%)	1 (2.0%)
Yes, both medical and psychiatric conditions		2 (7.7%)	2 (8.0%)	4 (7.8%)
Missing		0 (0.0%)	0 (0.0%)	0 (0.0%)
Education: n (%)		(n=26)	(n=25)	(n=51)
O' levels/ GCSEs/ Level 1 or 2 NVQ		13 (50.0%)	14 (56.0%)	27 (53.0%)
A' Levels/ Level 3 NVQ		5 (19.2%)	3 (12.0%)	8 (15.7%)
University degree/ Graduate Certificate or Diploma		5 (19.2%)	5 (20.0%)	10 (19.6%)

For peer review only - http://bmjopen.bmj.com/site/about/guidelines.xhtml

Postgraduate university degree (e.g., PGCE, MSc, MA, PhD)	2 (7.7%)	0 (0.0%)	2 (3.9%)
Missing	1 (3.9%)	3 (12.0%)	4 (7.8%)
Time since epilepsy diagnosis (years)			
N	26 (100.0%)	25 (100.0%)	51 (100%)
Mean	19.9	22.6	21.2
Standard deviation	14.85	18.38	16.57
Minimum	1.8	1.7	1.7
Median	16.8	19.3	17.3
Maximum	53.9	64.9	64.9
Missing	0 (0.0%)	0 (0.0%)	0 (0%)
Time since last epileptic seizure (days)			
N	23 (88.5%)	22 (88.0%)	45 (88.2%)
Mean	53.6	40.1	47.0
Standard deviation	101.10	61.21	83.34
Minimum	1	0	0
Median	14.0	10.5	14.0
Maximum	340	235	340
Missing	3 (11.5%)	3 (12.0%)	6 (11.8%)
Number of epileptic seizures in the last 12 months			
N	26 (100%)	25 (100%)	51 (100%)
None	1 (3.8%)	0 (0.0%)	1 (2.0%)
One	1 (3.8%)	0 (0.0%)	1 (2.0%)
Two	0 (0.0%)	2 (8.0%)	2 (3.9%)
Three	2 (7.7%)	1 (4.0%)	3 (5.9%)
Four	0 (0.0%)	0 (0.0%)	0 (0.0%)
Five	0 (0.0%)	2 (8.0%)	2 (3.9%)
Six	1 (3.8%)	3 (12.0%)	4 (7.8%)
Seven	1 (3.8%)	1 (4.0%)	2 (3.9%)
Eight	2 (7.7%)	2 (8.0%)	4 (7.8%)
Nine	0 (0.0%)	0 (0.0%)	0 (0.0%)
Ten or more	18 (69.2%)	14 (56.0%)	32 (62.8%)
Missing	0 (0.0%)	0 (0.0%)	0 (0%)

Notes:

[a] The Index of Multiple Deprivation (IMD) ranks every small area in England from 1 (most deprived area) to 32,844 (least deprived area).IMD rank and decile missing for one recruited participant as they resided in Wales.

For peer review only - http://bmjopen.bmj.com/site/about/guidelines.xhtml

Table 5 Number of ED visits patient participants made according to routine data

**Number of ED visits**		**SAFE+TAU**	**TAU**	**Total**
In the 12 months to baseline	N	25 (96.2%)	23 (92.0%)	48 (94.1%)
	Mean	2.1	3	2.5
	Standard deviation	2.22	2.76	2.51
	Minimum	0	1	0
	Median	1	2	2
	Maximum	10	12	12
	Missing	1 (3.8%)	2 (8.0%)	3 (5.9%)
In the 12 months following randomisation	N	25 (96.2%)	23 (92.0%)	48 (94.1%)
	Mean	1.8	3.4	2.6
	Standard deviation	3.14	4.78	4.05
	Minimum	0	0	0
	Median	1	2	1
	Maximum	12	20	20
	Missing	1 (3.8%)	2 (8.0%)	3 (5.9%)
Change from baseline over 12 months following randomisation	N	25 (96.2%)	23 (92.0%)	48 (94.1%)
	Mean	-0.3	0.4	0.1
	Standard deviation	1.99	3.81	2.99
	Minimum	-4	-5	-5
	Median	0	0	0
	Maximum	5	16	16
	Missing	1 (3.8%)	2 (8.0%)	3 (5.9%)

For peer review only - http://bmjopen.bmj.com/site/about/guidelines.xhtml

Table 6 Between intervention group differences in number of ED visits

Model and parameter	Parameter	Confidence Interval (CI)			P-value
		95%	90%	80%	
12 months following randomisation according to routine data[a]					
Negative binomial: SAFE + TAU (Rate Ratio)	0.54	0.24 to 1.18	0.28 to 1.04	0.32 to 0.90	0.12
Negative binomial: Dispersion parameter	1.53	0.67 to 2.39	0.80 to 2.25	0.96 to 2.09	NA
Vuong's Test[b]	-0.17	NA	NA	NA	0.87
12 months following randomisation according to routine data with adjustment for baseline ED visits					
Negative binomial: SAFE + TAU (Rate Ratio)	0.62	0.33 to 1.17	0.36 to 1.06	0.41 to 0.94	0.14
Negative binomial: Baseline ED visits (Rate Ratio)	1.33	1.18 to 1.52	1.20 to 1.49	1.23 to 1.45	<0.001
Negative binomial: Dispersion parameter	0.69	0.17 to 1.21	0.26 to 1.13	0.35 to 1.03	NA
Vuong's Test[b]	-0.13	NA	NA	NA	0.90

Notes:

ED=Emergency Department; SAFE=Seizure First Aid Training; TAU=Treatment-As-Usual,

[a] Analysis based on 48 patient participants

[b] Vuong's test p-value interpretation, a significantly negative parameter value favours the Negative Binomial model and significantly positive favours the Zero-inflated Negative Binomial model. A non-significant value indicates no significant difference between the models therefore the simpler Negative Binomial model is preferred.[75]

For peer review only

Figure Legend

Figure 1 CONSORT diagram of flow through the pilot trial

Figure 2 a) Bland Altman plot of agreement between self-reported ED use and routine data on ED use in 12 months prior to randomisation; b) Bland Altman plot of agreement between self-reported ED visits and routine data on ED use over 12 months following randomisation.

For peer review only

For peer review only - http://bmjopen.bmj.com/site/about/guidelines.xhtml

References

1. Dixon PA, Kirkham JJ, Marson AG, et al. National Audit of Seizure management in Hospitals (NASH): results of the national audit of adult epilepsy in the UK. *BMJ Open* 2015;5:e007325.
2. Rizos T, Jüttler E, Sykora M, et al. Common disorders in the neurological emergency room – experience at a tertiary care hospital. *European Journal of Neurology* 2011;18:430–35.
3. Royl G, Ploner CJ, Möckel M, et al. Neurological chief complaints in an emergency roo. *Der Nervenarzt* 2010;81(10):1226-30.
4. Marson T, Taylor C, Tudur-Smith C, et al. Seizure management in hospitals in Europe. ILAE 13th European Congress on Epileptology. Vienna, 2018.
5. Hart YM, Shorvon SD. The nature of epilepsy in the general population. II. Medical care. *Epilepsy research* 1995;21(1):51-58.
6. Jacoby A, Buck D, Baker G, et al. Uptake and costs of care for epilepsy: findings from a UK regional study. *Epilepsia* 1998;39(7):776-86.
7. Kitson A, Shorvon S. Services for patients with epilepsy: Report of a CSAG committee: Department of Health, 2000.
8. Dickson JM, Jacques R, Reuber M, et al. Emergency hospital care for adults with suspected seizures in the NHS in England 2007–2013: a cross-sectional study. *BMJ open* 2018;8(10):e023352.
9. Pearson M, Marson A, Dixon P, et al. National Audit of Seizure Management in Hospitals: St. Elsewhere's Hospital Clinical Report 2014 [Available from: http://www.nashstudy.org.uk/Newsletters/St%20Elsewhere's%20Clinical%20Report%20NASH%202.pdf accessed 9th April 2019.
10. Whiston S, Coyle B, Chappel D. Health needs assessment for long term neurological conditions in North East England. Stockton on Tees: North East Public Health Observatory 2009.
11. Noble AJ, Goldstein LH, Seed P, et al. Characteristics of people with epilepsy who attend emergency departments: Prospective study of metropolitan hospital attendees. *Epilepsia* 2012;53(10):1820–28.
12. NHS England. NHS Outcomes Framework indicators - Feb 2017 release. 2017 [Available from: https://digital.nhs.uk/data-and-information/publications/clinical-indicators/nhs-outcomes-framework/archive/nhs-outcomes-framework-indicators---february-2017-release accessed 8th August 2017.
13. House of Commons Committee of Public Accounts. Services to people with neurological conditions: progress review London House of Commons; 2015 [Available from: https://publications.parliament.uk/pa/cm201516/cmselect/cmpubacc/502/502.pdf accessed 4th April 2019.
14. Ridsdale L, McCrone P, Morgan M, et al. Can an epilepsy nurse specialist-led self-management intervention reduce attendance at emergency departments and promote well-being for people with severe epilepsy? A non-randomised trial with a nested qualitative phase. *Health Services and Delivery Research* 2013;1(9)
15. Dickson JM, Taylor LH, Shewan J, et al. Cross-sectional study of the prehospital management of adult patients with a suspected seizure (EPIC1). *BMJ Open* 2016;6:e010573.

For peer review only - http://bmjopen.bmj.com/site/about/guidelines.xhtml

16. Peter A Dixon, Jamie J Kirkham, Anthony G Marson, et al. National Audit of Seizure management in Hospitals (NASH): results of the national audit of adult epilepsy in the UK *BMJ Open* 2015;5(3):e007325.
17. Reuber M, Hattingh L, Goulding PJ. Epileptological emergencies in Accident and Emergency: a survey at St James's University Hospital, Leeds. *Seizure* 2000;9(3):216-20.
18. NHS Choices. What to do if someone has a seizure (fit) 2017 [Available from: https://www.nhs.uk/conditions/what-to-do-if-someone-has-a-seizure-fit/ accessed 4th April 2019.
19. Epilepsy Action. What to do when someone has a seizure 2016 [Available from: https://www.epilepsy.org.uk/info/firstaid/what-to-do accessed 4th April 2019.
20. Ashworth M, Seed P, Armstrong D, et al. The relationship between social deprivation and the quality of primary care: a national survey using indicators from the UK Quality and Outcomes Framework. *Br J Gen Pract* 2007;57(539):441-48.
21. Bautista RED, Glen ET, Wludyka PS, et al. Factors associated with utilization of healthcare resources among epilepsy patients. *Epilepsy research* 2008;79(2-3):120-29.
22. QIPP/Right Care. The NHS atlas of variation in healthcare 2010 [Available from: fingertips.phe.org.uk/documents/Atlas_2010%20Compendium.pdf.
23. Allard J, Shankar R, Henley W, et al. Frequency and factors associated with emergency department attendance for people with epilepsy in a rural UK population. *Epilepsy & Behavior* 2017;68:192-95.
24. Faught E, Duh MS, Weiner JR, et al. Nonadherence to antiepileptic drugs and increased mortality: findings from the RANSOM Study. *Neurology* 2008;71(20):1572-78.
25. Ridsdale L, Charlton J, Ashworth M, et al. Epilepsy mortality and risk factors for death in epilepsy: a population-based study. *Br J Gen Pract* 2011;61(586):e271-e78.
26. Health and Social Care Information Centre. Hospital Accident and Emergency Activity Supporting Information, 2017-18 2018 [cited Available from: https://files.digital.nhs.uk/8B/94BB9E/AE1718_supporting_information.pdf accessed 27th August 2019.
27. International League Against Epilepsy - British Branch. Epilepsy health services for epilepsy - the proceedings of an expert workshop London2016 [Available from: https://ilaebritish.org.uk/epilepsy-emergency-care/ accessed 22nd October 2019.
28. National Clinical Guideline Centre. Pharmacological Update of Clinical Guideline 20. The Epilepsies: The Diagnosis and Management of the Epilepsies in Adults and Children in Primary and Secondary Care London: National Clinical Guideline Centre (UK); 2012 [Available from: ncbi.nlm.nih.gov/pubmedhealth/PMH0068980.
29. Noble AJ, Morgan M, Virdi C, et al. A nurse-led self-management intervention for people who attend emergency departments with epilepsy: the patients' view. *Journal of Neurology* 2013;260(4):1022–30.
30. Ridsdale L, Virdi C, Noble AJ, et al. Explanations given by people with epilepsy for using emergency medical services: a qualitative study. *Epilepsy Behaviour* 2012;25:529–33.
31. Noble AJ, Marson AG, Tudur-Smith C, et al. 'Seizure First Aid Training' for people with epilepsy who attend emergency departments, and their family and

For peer review only - http://bmjopen.bmj.com/site/about/guidelines.xhtml

friends: study protocol for intervention development and a pilot randomised controlled trial. *BMJ Open* 2015;5:e009040.
32. Snape DA, Morgan M, Ridsdale L, et al. Developing and assessing the acceptability of an epilepsy first aid training intervention for patients who visit UK emergency departments: A multi-method study of patients and professionals. *Epilepsy and Behavior* 2017;68:177-85.
33. Craig P, Dieppe P, Macintyre S, et al. Developing and evaluating complex interventions: the new Medical Research Council guidance. *Bmj* 2008;337:a1655.
34. Lancaster GA, Dodd S, Williamson PR. Design and analysis of pilot studies: recommendations for good practice. *Journal of Evaluation in Clinical Practice* 2004 10(2):307-12.
35. Department of Health. Health Profile 2012. Liverpool. 2012 [Available from: http://fingertipsreports.phe.org.uk/health-profiles/2012/e08000012.pdf accessed 11th April 2018.
36. Department of Health. Health Profile 2012. Wirral 2012 [Available from: http://fingertipsreports.phe.org.uk/health-profiles/2012/e08000015.pdf accessed 11th April 2018.
37. Noble AJ, Snape D, Ridsdale L, et al. Assessing treatment fidelity within an epilepsy randomized controlled trial: Seizure first aid training for people with epilepsy who visit emergency departments. *Behavioural Neurology* 2019;19:1-11.
38. Sim J, Lewis M. 2012. *Journal of Clinical Epidemiology* The size of a pilot study for a clinical trial should be calculated in relation to considerations of precision and efficiency;65:301-08.
39. Julious SA. Sample size of 12 per group rule of thumb for a pilot study. *Pharmaceutical Statistics* 2005;4:287–91.
40. Digital N. Accident and Emergency Attendances in England - 2012-13 2014 [Available from: https://digital.nhs.uk/data-and-information/publications/statistical/hospital-accident--emergency-activity/accident-and-emergency-attendances-in-england-2012-13 accessed 22nd October 2019.
41. Bhatt H, Matharu MS, Henderson K, et al. An audit of first seizures presenting to an Accident and Emergency Department. *Seizure* 2005;14:58-61.
42. Bland JM, Altman D. Statistical methods for assessing agreement between two methods of clinical measurement. *The Lancet* 1986;327:307-10.
43. Lee EC, Whitehead AL, Jacques RM, et al. The statistical interpretation of pilot trials: should significance thresholds be reconsidered? *BMC Medical Research Methodology* 2014;14:41.
44. Noble AJ, Marson AG. Which outcomes should we measure in adult epilepsy trials? The views of people with epilepsy and informal carers. *Epilepsy and Behavior* 2016;59:105-10.
45. Keene ON, Jones MR, Lane PW, et al. Analysis of exacerbation rates in asthma and chronic obstructive pulmonary disease: example from the TRISTAN study. *Pharmaceutical Statistics* 2007;6:89-97.
46. Sach TH, Dsborough J, Houghton J, et al. Applying microcosting methods to estimate the costs of pharmacy interventions: an illustration using multiprofessional clinical medication reviews in care homes for older people. *International Journal of Pharmacy Practice* 2015;23:237-47.

For peer review only - http://bmjopen.bmj.com/site/about/guidelines.xhtml

47. Lopetegui M, Yen PY, Lai A, et al. Time motion studies in healthcare: what are we talking about? *Journal of Biomedical Informatics* 2014;49: 292-99.
48. Piccenna L, Walker W, Shears G, et al. Priorities for epilepsy research: A briefing report 2017 [Available from: https://pdfs.semanticscholar.org/5861/867345dc168e35918e386e7beaa89c7c6586.pdf accessed 23rd October 2019.
49. Zarit SH, Reever KE, Bach-Peterson J. Relatives of the impaired elderly: correlates of feelings of burden. *Gerontologist* 1980;20:649-55.
50. Crawford JR, Henry JD, Crombie C, et al. Normative data for the HADS from a large non-clinical sample. *British Journal of Clin Psychology* 2001;40(4):429-34.
51. Walters SJ, Bonacho Dos Anjos Henriques-Cadby I, Bortolami O, et al. Recruitment and retention of participants in randomised controlled trials: a review of trials funded and published by the United Kingdom Health Technology Assessment Programme. *BMJ Open* 2017;7:e015276.
52. Monfared AA, Lelorier J. Accuracy and validity of using medical claims data to identify episodes of hospitalizations in patients with COPD. *Pharmacoepidemiology and drug safety* 2006;15:19-29.
53. Roberts RO, Bergstralh EJ, Schmidt L, et al. Comparison of self-reported and medical record health care utilization measures. *Journal of Clinical Epidemiology* 1996;49:989-95.
54. Medical Research Council. Obtaining data from NHS Digital for health research – a guide for researchers 2016 [Available from: https://mrc.ukri.org/documents/pdf/obtaining-data-from-nhs-digital-v101016/ accessed 10th October 2016.
55. Raftery J, Young A, Stanton L, et al. Clinical trial metadata: defining and extracting metadata on the design, conduct, results and costs of 125 randomised clinical trials funded by the National Institute for Health Research Health Technology Assessment programme. *Health Technology Assessment* 2015;19:1-138.
56. National Institute for Health Research. HTA Supporting Information: Reasonable costs and value for money 2019 [Available from: https://www.nihr.ac.uk/documents/hta-supporting-information/11929#Reasonable_costs_and_value_for_money accessed 22nd October 2019.
57. Ridsdale L, Wojewodka G, Robinson EJ, et al. The effectiveness of a group self-management education course for adults with poorly controlled epilepsy, SMILE (UK): A randomized controlled trial. *Epilepsia* 2018 59(5):1048-61.
58. Ridsdale L, Wojewodka G, Robinson E, et al. Characteristics associated with quality of life among people with drug-resistant epilepsy. *Journal of Neurology* 2017;246(6):1174-84.
59. Hewitson P, Ward AM, Heneghan C, et al. Primary care endorsement letter and a patient leaflet to improve participation in colorectal cancer screening: results of a factorial randomised trial. *British Journal of Cancer* 2011;105(4):475-80.
60. Sajatovic M, Colon-Zimmermann K, Kahriman M, et al. A 6-month prospective randomized controlled trial of remotely delivered group format epilepsy self-management versus waitlist control for high-risk people with epilepsy. *Epilepsia* 2018 59(9):1684-95.
61. Luedke MW, Blalock DV, Goldstein KM, et al. Self-management of epilepsy: A systematic review. *Annals of Internal Medicine* 2019 171(2):117-26.

For peer review only - http://bmjopen.bmj.com/site/about/guidelines.xhtml

62. Thabane L, Ma J, Chu R, et al. A tutorial on pilot studies: the what, why and how. *BMC Medical Research Methodology* 2010;10(1)
63. Eldridge SM, Chan CL, Campbell MJ, et al. CONSORT 2010 statement: extension to randomised pilot and feasibility trials. *British Medical Journal* 2016;355:i5239.
64. Thapar A, Kerr M, Harold G. Stress, anxiety, depression, and epilepsy: Investigating the relationship between psychological factors and seizures. *Epilepsy and Behavior* 2009;14:134-40.
65. Cramer J, Perrine K, Devinsky O, et al. Development and cross-cultural translations of a 31-item quality of life in epilepsy inventory. *Epilepsia* 1998;39:81-88.
66. Zigmond AS, Snaith RP. The hospital anxiety and depression scale. *Acta psychiatrica Scandinavica* 1983;67:361-70.
67. Taylor J, Baker GA, Jacoby A. Levels of epilepsy stigma in an incident population and associated factors. *Epilepsy and Behavior* 2011;21(255-260)
68. Mittan R. Fear of seizures. In: Whitman S, Hermann B, eds. Psychopathology in epilepsy: social dimensions New York, NY: Oxford University Press 1986:90–121.
69. Martiniuk ALC, Campbell MK. Development and psychometric properties of the Thinking about Epilepsy questionnaire assessing children's knowledge and attitudes about epilepsy. *Epilepsy and Behavior* 2007;10:595-603.
70. Wagner AK, Keller SD, Kosinski M, et al. Advances in methods for assessing the impact of epilepsy and antiepileptic drug therapy on patients' health-related quality of life. *Qual of Life Research* 1995;4:115-34.
71. Shore CP, Austin JK, Dunn DW. Maternal adaptation to a child's epilepsy. *Epilepsy and Behavior* 2004;5:557-68.
72. Beecham J, Knapp M. Costing psychiatric interventions. In: Thornicroft G, Brewin C, Wing J, eds. Measuring Mental Health Needs. London: Gaskell 1992: 163-83.
73. Williams A. The role of the EUROQOL instrument in QALY calculations. York: Centre for Health Economics, University of York, 1995.
74. Smyth RM, Duley L, Jacoby A, et al. Women's experiences of participating in the Magpie Trial: a postal survey in the United Kingdom. *Birth* 2009;36:220-29.
75. Vuong Q. Likelihood ratio tests for model selection and non-nested hypotheses. *Econometrica* 1989;B:307- 33.

For peer review only

For peer review only - http://bmjopen.bmj.com/site/about/guidelines.xhtml

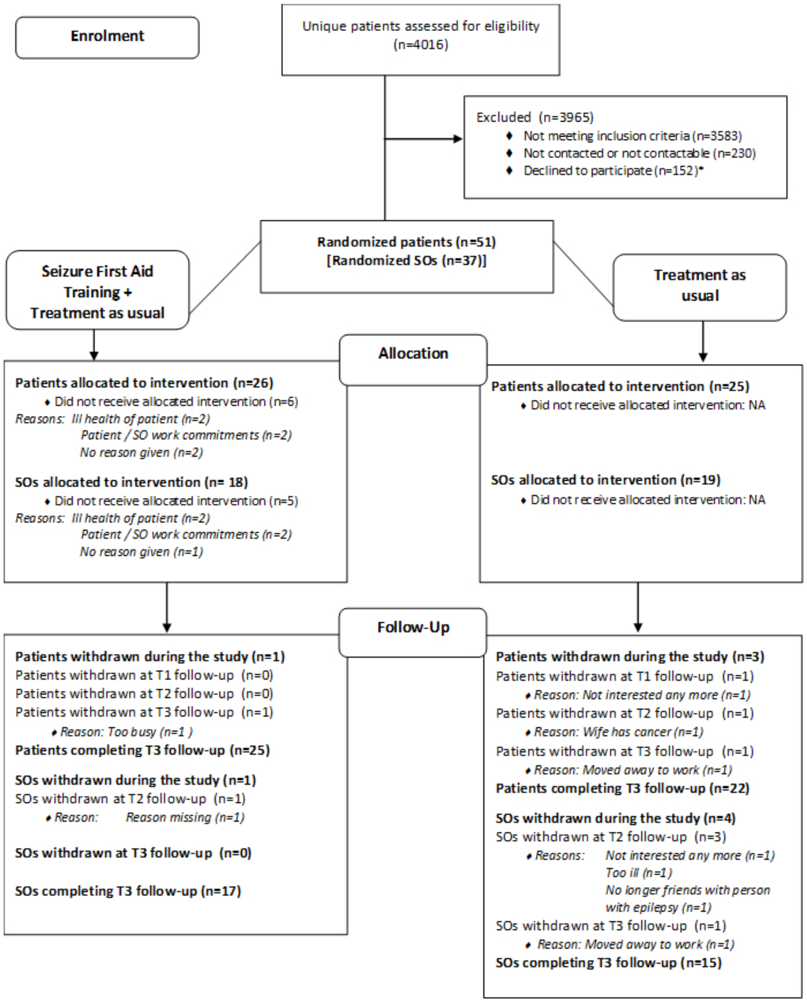

Figure 1 CONSORT diagram of flow through the pilot trial

190x275mm (96 x 96 DPI)

For peer review only - http://bmjopen.bmj.com/site/about/guidelines.xhtml

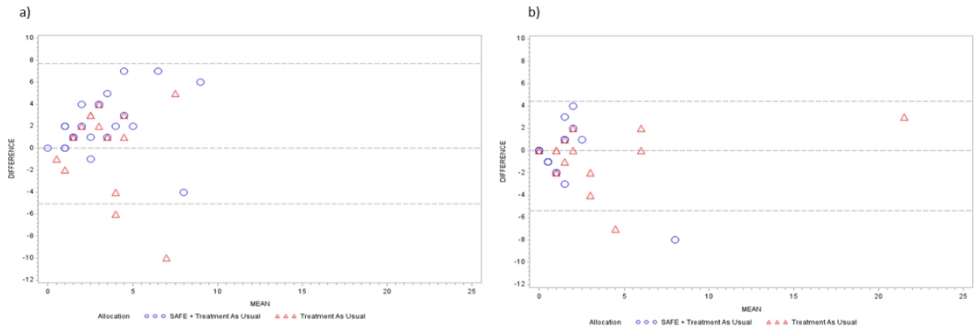

Figure 2 a) Bland Altman plot of agreement between self-reported ED use and routine data on ED use in 12 months prior to randomisation; b) Bland Altman plot of agreement between self-reported ED visits and routine data on ED use over 12 months following randomisation.

338x190mm (96 x 96 DPI)

SUPPLEMENTARY FILES

Supplementary File 1 Search criteria employed at the different recruitment sites to examine their electronic unique attendance record systems to identify potentially suitable persons attending ED for epilepsy

<table>
<tr><th colspan="3">Recruitment site</th></tr>
<tr><th>Site
Aintree University Hospital</th><th>Site
Royal Liverpool University Hospital</th><th>Site
Arrowe Park Hospital</th></tr>
<tr>
<td>Identify those persons who within the free text box ‘presenting complaint’ are recorded as having visited the ED for a:

• “fit”
• “epilepsy”
• “convulsion”
• “seizure”

And those patients admitted as an emergency from ED to a ward with any of the following primary or secondary ICD-10 diagnoses:

• “G40 Epilepsy”
• “G41 Status epilepticus”
• “G83.8 Other specified paralytic syndromes”
• “R56.8 Other and unspecified convulsions”</td>
<td>Identify those persons who attended the ED and were given any of the following presenting complaint-discharge diagnosis profiles:

• Presenting complaint “AE20 AEPRC Fit” AND ED discharge diagnosis “02 AEDIG Contusion/ abrasion”;
• “AE20 AEPRC Fit” AND “04 AEDIG Head injury”;
• “AE20 AEPRC Fit” AND “05 AEDIG Dislocation”;
• “AE20 AEPRC Fit” AND “06 AEDIG Sprain/ ligament injury”;
• “AE20 AEPRC Fit” AND “07 AEDIG Muscle/ tendon injury”;
• “AE20 AEPRC Fit” AND “10 AEDIG Burns and scalds”;
• “AE20 AEPRC Fit” AND “15 AEDIG Near Drowning”;
• “AE20 AEPRC Fit” AND “33 AEDIG Facio-maxillary conditions”;
• “AE20 AEPRC Fit” AND “38 AEDIG Diagnosis not classifiable”.
• “241 AEDIG Epilepsy”.

And those patients admitted as an emergency from ED to a ward with any of the following primary or secondary ICD-10 diagnoses:

• “G40 Epilepsy”
• “G41 Status epilepticus”
• “G83.8 Other specified paralytic syndromes”
• “R56.8 Other and unspecified convulsions”</td>
<td>Identify those persons who within the free text box ‘reason for visit’ or ‘presenting complaint’ are recorded as having visited the ED for:

• “fit”
• “epilep”
• “convuls”
• “seiz”

And those patients admitted as an emergency from ED to a ward with any of the following primary or secondary ICD-10 diagnoses:

• “G40 Epilepsy”
• “G41 Status epilepticus”
• “G83.8 Other specified paralytic syndromes”
• “R56.8 Other and unspecified convulsions”</td>
</tr>
</table>

Notes:

As the architectures of the systems and the coding processes at each sites varied the research team worked with local ED teams to identify the search criteria.

For peer review only

Supplementary File 2 Serious Adverse Event Protocol

Adapted from the Medicines for Human Use (Clinical Trials) Regulations 2004 (SI 2004/1031), http://www.legislation.gov.uk/uksi/2004/1031/pdfs/uksi_20041031_en.pdf [accessed 29th August, 2019] a serious adverse event (SAE) was defined as an adverse event which resulted in any of the following:

- Death
- Was life-threatening (subject at immediate risk of death, e.g., status epilepticus)
- Seizure resulting in hospital admission for ≥24 hours
- Emergency attendance or hospital admission for reason other than seizure
- Results in persistent or significant disability or incapacity, or
- Was otherwise considered medically significant.

'Life-threatening' in the definition of 'serious' referred to an event in which the patient was at risk of death at the time of the event; it did not refer to an event which hypothetically might have caused death if it were more severe. Hospitalisations for a pre-existing condition, including elective procedures that had not worsened, did not constitute an SAE. Prolongation of hospital stay due to social factors, for example, geographical location of the participant's home which prevented discharge was also not considered a SAE.

Given the characteristics of the subject population being studied, the following events were expected in this study population and were not recorded as part of the SAE monitoring process:

- Epileptic seizures with or without injury;
- Emergency or urgent medical attention. This includes visiting a hospital emergency department with the duration of the stay lasting <24 hours, attending an NHS out of hours primary care service (NHS Urgent Care 24), telephoning for an ambulance, telephoning NHS 111, seeking/ having an urgent/ fast-tracked appointment with a usual care provider (GP or specialist) or other registered health professional (e.g., a pharmacist);
- Side-effects of AED medication;
- Diagnosis of a comorbid psychiatric condition.

A delegated medically qualified person within the team (AM; LR) assessed each unexpected SAE. This person considered information on the temporal and physical relationship between the event and possible causes and assessed whether the event was related or unrelated to the patient's participation in the study. Further details on the process followed detailed below.

Monitoring

As part of this trial patient participants did not receive additional medical reviews. There is also no 'live' system which can be used to track SAEs such as emergency admissions and usual care providers are not systematically informed of them. Therefore, to monitor SAEs the research team liaised with patient participants themselves. A standardised form was to be completed at 3- (T1, by telephone), 6- (T2, by telephone) and 12-months (T3, during a face-to-face appointment) post-randomisation to collect information on patient participants' experience of unexpected, SAEs.

In each instance, a maximum of 3 attempts was made to contact the patient participant by telephone (including trying to contact them via their informal carer if they are taking part with one). If the patient was

For peer review only

not be contactable, a letter was be sent the patient's GP asking them to inform the research team if the patient was no longer alive and the circumstances of their death.

Causality

One of the delegated medically qualified persons within the team assessed each unexpected SAE. They considered information on the temporal and physical relationship between the event and possible causes and assessed whether the event was related or unrelated to the patient's participation in the study. In doing this, they used the definitions in the Table below.

Table Definitions of causality for serious adverse event

	**Description**
**Unrelated**	There is no evidence of any causal relationship. There is an alternative cause for the SAE.
**Unlikely**	There is little evidence to suggest there is a causal relationship (e.g. the event did not occur within a reasonable time after receipt of the intervention). There is another reasonable explanation for the event (e.g. the participant's clinical condition, other concomitant treatment).
**Possibly**	There is some evidence to suggest a causal relationship (e.g. because the event occurs within a reasonable time after receipt of the intervention). However, the influence of other factors may have contributed to the event (e.g. the participants clinical condition, other concomitant treatment).
**Probably**	There is evidence to suggest a causal relationship and the influence of other factors is unlikely.
**Almost certainly**	There is clear evidence to suggest a causal relationship and other possible contributing factors can be ruled out.

Reporting

As per National Research Ethics Service (2015) guidelines for non-CTIMPs, the main ethics committee approving the study and its Sponsor were informed within 15 days of the team becoming aware of any SAE that in the opinion of the medical reviewer was both unexpected (that is, the type of event is not listed in the protocol as an expected occurrence) and judged to be "possibly", "probably" or "almost certainly" related to participation in the study (that is, it resulted from administration of any of the research procedures, including the intervention).

For peer review only - http://bmjopen.bmj.com/site/about/guidelines.xhtml

Supplementary File 3 CONSORT diagram of eligibility screening for the trial

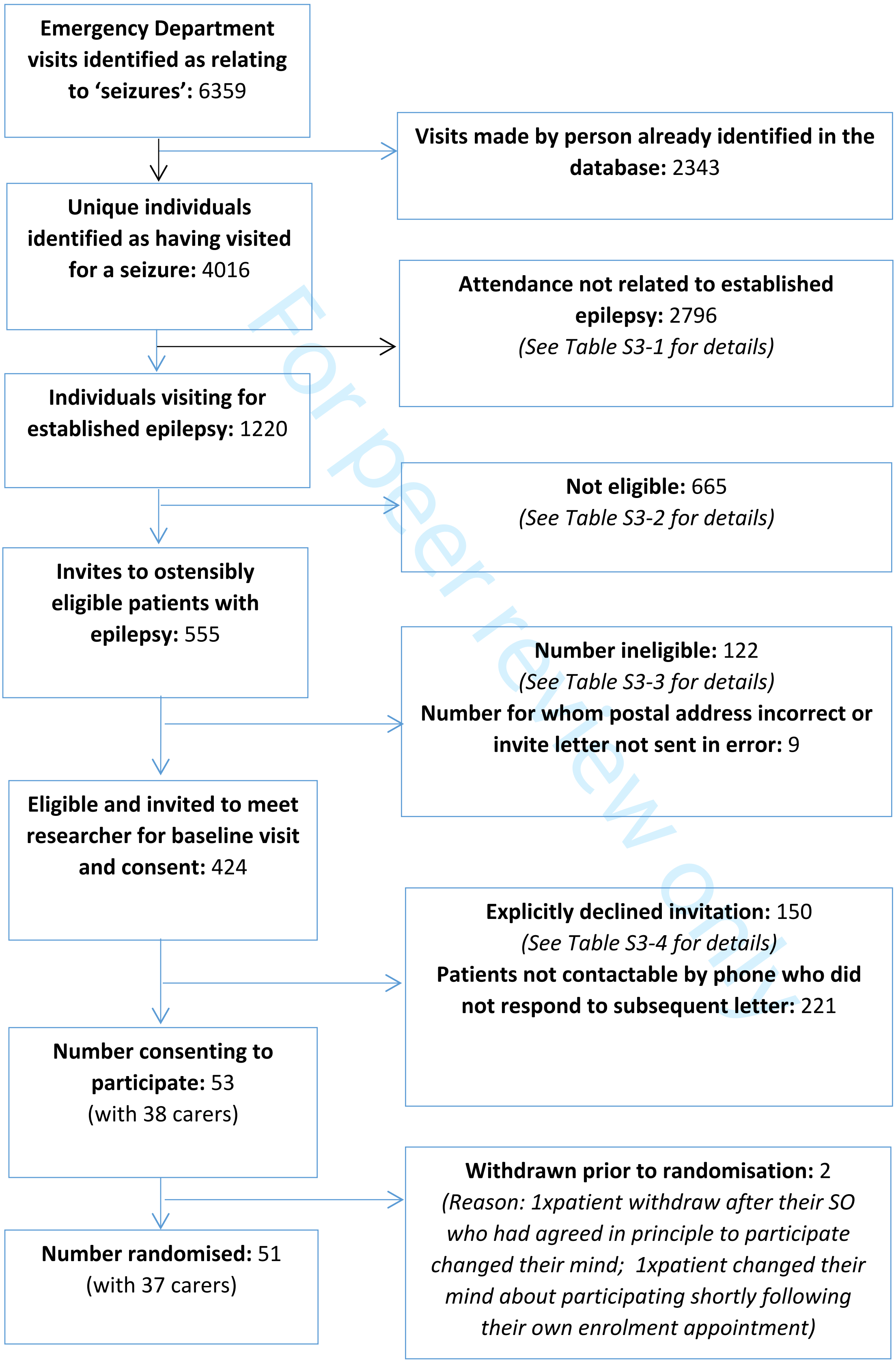

For peer review only

Table S3-1 Attendance at emergency department not related to epilepsy

Reason	Number of participants			
	Site 1	Site 2	Site 3	Total
No confirmed diagnosis of epilepsy/not attending because of epilepsy	737	483	715	1935
Acute symptomatic seizures related to neurological illness or substance abuse	238	240	191	669
Psychogenic non-epileptic seizures	19	55	29	103
Medical record missing	54	5	27	86
Ineligible, but no reason given	2	1	0	3
**Total**	**1050**	**784**	**962**	**2796**

Table S3-2 Other reasons for non-eligibility (individuals visiting for established epilepsy)

Reason	Number of participants			
	Site 1	Site 2	Site 3	Total
Postcode outside catchment area	148	94	60	302
Learning disability likely to impede individuals' capacity to provided signed, informed consent	45	51	52	148
Inability to converse in English and provide signed informed consent	10	24	46	80
Life threatening medical illness	35	26	10	71
Severe psychiatric disorder	11	3	18	32
No fixed abode	10	6	13	29
Participating in another trial	3	0	0	3
**Total**	**262**	**204**	**199**	**665**

Table S3-3 Reasons for non-eligibility (ostensibly eligible individuals)

Reason	Number of participants			
	Site 1	Site 2	Site 3	Total
Has not visited an ED for epilepsy on ≥2 occasions within previous 12 months (as reported by patient)	9	15	24	48
Not able to provide informed consent, participate in SAFE course if randomised or to independently complete questionnaires in English	9	7	7	23
Ineligible, but no reason given[a]	4	7	4	15
Not established diagnosis of epilepsy (<1 year)	3	2	5	10
Moved out of area; postcode no longer within 25 mile catchment area	4	1	3	8
Actual or suspected psychogenic non-epileptic seizures alone or in combination with epilepsy	2	1	4	7
Severe current psychiatric disorders or life-threatening medical illness	1	5	1	7
Not currently being prescribed AED/s	1	0	1	2

Acute symptomatic seizures related to acute neurological illness or substance misuse	0	1	0	1
Participating in another trial	1	0	0	1
**Total**	**34**	**39**	**49**	**122**

Notes:

a. Lack of reason due to patients returning participation slips and no stating actual reason for ineligibility

Table S3-4 Reasons for declining participation (eligible individuals)

**Reason**	**Number of participants**			
	**Site 1**	**Site 2**	**Site 3**	**Total**
Not interested	30	11	23	64
Too busy	11	12	11	34
No reason given	9	10	8	27
Too ill	5	5	9	19
Too well	3	1	2	6
**Total**	**58**	**39**	**53**	**150**

For peer review only

Supplementary File 4 Demographic characteristics of eligible patients who did and did not agree to participate

Demographic characteristic		Agreed to participate	Did not agree to participate
Sex: n (%)		(n=51)	(n=379)[a]
Male		22 (43.1%)	192 (50.7%)
Female		29 (56.9%)	187 (49.3%)
Missing		0 (0.0%)	0 (0%)
Age at presentation to the emergency department (years)			
N		51 (100.0%)	379 (100%)
Mean		39.9	40.6
Standard deviation		15.66	16.83
Minimum		16.4	16.2
Median		38.8	37.5
Maximum		71.3	84.4
Missing		0 (0.0%)	0 (0%)
Index of Multiple Deprivation (IMD)[b]		(n=51)	(n=379)
IMD Decile: 1	n (%)	25 (49.0%)	188 (49.6%)
IMD Rank	Minimum	44	28
	Median	673.0	924.5
	Maximum	3202	3107
IMD Decile: 2 to 3	n (%)	13 (25.4%)	82 (21.6%)
IMD Rank	Minimum	3989	3291
	Median	6785	5309
	Maximum	8281	9835
IMD Decile: 4 to 6	n (%)	6 (11.8%)	59 (15.6%)
IMD Rank	Minimum	9881	10277
	Median	11924	14459
	Maximum	16004	19659
IMD Decile: 7 to 10	n (%)	6 (11.8%)	50 (13.2%)
IMD Rank	Minimum	24971	19826
	Median	28876	22673
	Maximum	32724	32396
IMD Decile missing[a]	n (%)	1 (2.0%)[b]	0 (0%)

Notes:

[a] Includes seven patients who could not be contacted due to an incorrect postal address and two patients who were not sent an invite letter in error.

[b] The Index of Multiple Deprivation (IMD) ranks every small area in England from 1 (most deprived area) to 32,844 (least deprived area).

[c] IMD rank and decile missing for one recruited participant as they resided in Wales.

For peer review only

Supplementary File 5 Demographic characteristics of SO participants

Demographic characteristic	Seizure First Aid Training + TAU	Treatment as usual (TAU)	Total
Relationship with patient participant	(n=18)	(n=19)	(n=37)
Parent	4 (22.2%)	4 (21.1%)	8 (21.6%)
Son / daughter	2 (11.1%)	4 (21.1%)	6 (16.2%)
Grandparent	0 (0.0%)	0 (0.0%)	0 (0.0%)
Spouse / partner	9 (50.0%)	7 (36.8%)	16 (43.2%)
Sibling	1 (5.6%)	0 (0.0%)	1 (2.7%)
Cousin, aunt / uncle	0 (0.0%)	0 (0.0%)	0 (0.0%)
Niece / nephew	0 (0.0%)	1 (5.3%)	1 (2.7%)
Friend	2 (11.1%)	3 (15.8%)	5 (13.5%)
Other	0 (0.0%)	0 (0.0%)	0 (0.0%)
Missing	0 (0.0%)	0 (0.0%)	0 (0.0%)
Co-habitation with patient participant	(n=18)	(n=19)	(n=37)
Yes	14 (77.8%)	14 (73.4%)	28 (75.7%)
No	4 (22.2%)	5 (26.3%)	9 (24.3%)
Missing	0 (0.0%)	0 (0.0%)	0 (0.0%)
Contact with patient participant (number of days per week)	(n=18)	(n=19)	(n=37)
None	0 (0.0%)	0 (0.0%)	0 (0.0%)
One	1 (5.6%)	0 (0.0%)	1 (2.7%)
Two	0 (0.0%)	1 (5.3%)	1 (2.7%)
Three	0 (0.0%)	2 (10.5%)	2 (5.4%)
Four	0 (0.0%)	0 (0.0%)	0 (0.0%)
Five	0 (0.0%)	0 (0.0%)	0 (0.0%)
Six	0 (0.0%)	0 (0.0%)	0 (0.0%)
Seven (every day)	17 (94.4%)	16 (84.2%)	33 (89.2%)
Missing	0 (0.0%)	0 (0.0%)	0 (0.0%)
Sex: n (%)	(n=18)	(n=19)	(n=37)
Male	6 (33.3%)	9 (47.4%)	15 (40.5%)
Female	12 (67.7%)	10 (52.6%)	22 (59.5%)
Missing	0 (0.0%)	0 (0.0%)	0 (0.0%)
Age at consent into the trial (years)			
N	17 (94.4%)	19 (100.0%)	36 (94.6%)
Mean	41.3	44.9	43.2
Standard deviation	18.66	15.69	17.01
Minimum	17.8	18.1	17.8
Median	43.5	49.7	48.0
Maximum	79.7	71.5	79.7
Missing	1 (5.6%)	0 (0.0%)	1 (2.7%)
Ethnicity: n (%)	(n=18)	(n=19)	(n=37)
White	18 (100.0%)	18 (94.5%)	36 (97.3%)
Asian / Asian British	0 (0.0%)	0 (0.0%)	0 (0.0%)
Black / African / Carribean / Black British	0 (0.0%)	0 (0.0%)	0 (0.0%)
Mixed / multiple ethnic groups	0 (0.0%)	0 (0.0%)	0 (0.0%)
Other ethnic group	0 (0.0%)	1 (5.3%)	1 (2.7%)
Missing	0 (0.0%)	0 (0.0%)	0 (0.0%)
Significant medical history: n (%)	(n=18)	(n=19)	(n=37)
No, none	13 (72.2%)	12 (63.2%)	25 (67.6%)
Yes, a medical condition	5 (27.8%)	6 (31.6%)	11 (29.7%)
Yes, a psychiatric condition	0 (0.0%)	0 (0.0%)	0 (0.0%)
Yes, both medical and psychiatric conditions	0 (0.0%)	1 (5.3%)	1 (2.7%)
Missing	0 (0.0%)	0 (0.0%)	0 (0.0%)

Education: n (%)	(n=18)	(n=19)	(n=37)
O' levels/ GCSEs/ Level 1 or 2 NVQ	9 (50.0%)	14 (73.7%)	23 (62.2%)
A' Levels/ Level 3 NVQ	4 (22.2%)	2 (10.5%)	6 (16.2%)
University degree/ Graduate Certificate or Diploma	5 (27.8%)	3 (15.8%)	8 (21.6%)
Postgraduate university degree (e.g., PGCE, MSc, MA, PhD)	0 (0.0%)	0 (0.0%)	0 (0.0%)
Missing	0 (0.0%)	0 (0.0%)	0 (0.0%)

For peer review only

Supplementary File 6 Measure completeness by assessment point, measure and participant type

Table S6.1 Number of patient participants completing assessment tools

Study assessment tool and timepoint	SAFE + TAU	TAU	Total
**Patient Participants: Baseline (T0)**	**(n=26)**	**(n=25)**	**(n=51)**
ED visits (self-report)	24 (92.3%)	21 (84.0%)	45 (88.2%)
Seizure control: Thapar scale	26 (100%)	25 (100%)	51 (100%)
Quality of Life: QOLIE-31P	23 (88.5%)	16 (64.0%)	39 (76.5%)
Distress: HADS [a]	23 (88.5%)	24 (96.0%)	47 (92.2%)
Felt stigma: Stigma of Epilepsy Scale	24 (92.3%)	24 (96.0%)	48 (94.1%)
Health economics: Client Service Receipt Inventory [b]	19 (73.1%)	17 (68.0%)	36 (70.6%)
Health economics: EuroQol-5D	21 (80.8%)	25 (100.0%)	46 (90.2%)
Confidence managing seizures/ epilepsy: Epilepsy Mastery Scale	26 (100.0%)	21 (84.0%)	47 (92.2%)
Fear of seizures:, Epilepsy Knowledge and Management Questionnaire Fears subscale	16 (61.5%)	9 (36.0%)	25 (49.2%)
Knowledge of what to do: Thinking about Epilepsy Questionnaire	26 (100%)	25 (100%)	51 (100%)
**Patient Participants: 6 months (T2)**	**(n=26)**	**(n=23)[c]**	**(n=49)[c]**
Quality of Life: QOLIE-31P	16 (61.5%)	12 (52.2%)	28 (57.1%)
Confidence managing seizures/ epilepsy: Epilepsy Mastery Scale	21 (80.8%)	15 (65.2%)	36 (73.5%)
Seizure control: Thapar scale	16 (61.5%)	15 (65.2%)	28 (57.1%)
**Patient Participants: 12 months (T3)**	**(n=25)[d]**	**(n=22)[d]**	**(n=47)[d]**
ED visits (self-report)	17 (68.0%)	17 (77.3%)	34 (72.3%)
Seizure control: Thapar scale	20 (80.0%)	14 (60.9%)	34 (72.3%)
Quality of Life: QOLIE-31P	10 (40.0%)	8 (36.4%)	18 (38.3%)
Distress: HADS [a]	18 (72.0%)	17 (77.3%)	35 (74.4%)
Felt stigma: Stigma of Epilepsy Scale	18 (72.0%)	17 (77.3%)	35 (74.4%)
Health economics: Client Service Receipt Inventory [b]	13 (52.0%)	10 (45.5%)	23 (48.9%)
Health economics: EuroQol-5D	18 (72.0%)	17 (77.3%)	35 (74.4%)
Confidence managing seizures/ epilepsy: Epilepsy Mastery Scale	18 (72.0%)	16 (72.7%)	34 (72.3%)
Fear of seizures: Epilepsy Knowledge and Management Questionnaire Fears subscale	8 (32.0%)	5 (22.7%)	13 (27.7%)
Knowledge of what to do: Thinking about Epilepsy Questionnaire	18 (72.0%)	17 (77.3%)	35 (74.4%)
Feedback on participation	17 (68.0%)	15 (68.2%)	32 (68.1%)

Notes:

[a] Completeness of the whole HADS scale (both anxiety and depression subscales)

[b] Only six mandatory questions counted. Conditional questions (e.g. if yes, then…) not counted towards total completion

[c] Two patient participants from the TAU group had withdrawn by the T2 visit

[d] Four patient participants (three from the TAU group and one from the SAFE + TAU group) had withdrawn by the T3 visit

[e] Note that whilst some participants did not fully complete all items within a questionnaire, this does not automatically mean their data would need to be excluded from analysis of a change in that domain since for some measures test developers permit imputation when the extent of missing items is low and follows a specific pattern.

For peer review only

Table S6.2 Number of SO participants completing study assessment tools

Study assessment tool and time point	SAFE + TAU	TAU	Total
**Significant other Participants: Baseline (T0)**	**(n=18)**	**(n=19)**	**(n=37)**
Burden: Zarit Caregiver Burden	17 (94.4%)	17 (89.5%)	34 (91.9%)
Distress: HADS [a]	17 (94.4%)	18 (94.7%)	35 (94.6%)
Confidence managing seizures/ epilepsy: Parent Response to Child Illness Scale	18 (100.0%)	19 (100.0%)	37 (100.0%)
Fear of seizures: Epilepsy Knowledge and Management Questionnaire Fears subscale	6 (33.3%)	4 (21.1%)	10 (27.0%)
Knowledge of what to do: Thinking about Epilepsy Questionnaire	18 (100.0%)	19 (100.0%)	37 (100.0%)
**Significant other Participants: 6 months (T2)**	**(n=17)[b]**	**(n=16)[b]**	**(n=33)[b]**
Burden: Zarit Caregiver Burden	15 (88.2%)	8 (50.0%)	23 (69.7%)
Confidence managing seizures/ epilepsy: Parent Response to Child Illness Scale	16 (94.1%)	9 (56.3%)	25 (75.8%)
**Significant other Participants: 12 months (T3)**	**(n=17)[c]**	**(n=15)[c]**	**(n=32)[c]**
Burden: Zarit Caregiver Burden	11 (64.7%)	10 (66.7%)	21 (65.6%)
Distress: HADS [a]	11 (64.7%)	10 (66.7%)	21 (65.6%)
Confidence managing seizures/ epilepsy: Parent Response to Child Illness Scale	11 (64.7%)	10 (66.7%)	21 (65.6%)
Fear of seizures: Epilepsy Knowledge and Management Questionnaire Fears subscale	6 (35.3%)	2 (13.3%)	8 (25.0%)
Knowledge of what to do: Thinking about Epilepsy Questionnaire	15 (88.2%)	13(86.7%)	28 (87.5%)
Feedback on participation	11 (64.7%)	9 (60.0%)	20 (62.5%)

Notes:

[a] Completeness of the whole HADS scale (both anxiety and depression subscales),

[b] Four SO participants (one in the SAFE group and three in the TAU group) had withdrawn by the T2 visit

[c] Five SO participants (one in the SAFE group and four in the TAU group) had withdrawn by the T3 visit

[d] Note that whilst some participants did not fully complete all items within a questionnaire, this does not automatically mean their data would need to be excluded from analysis of a change in that domain since for some measures test developers permit imputation when the extent of missing items is low and follows a specific pattern.

Supplementary File 7 Histogram of number of emergency department attendances in 12 prior to randomisation by n=48 patient participants according to routine data

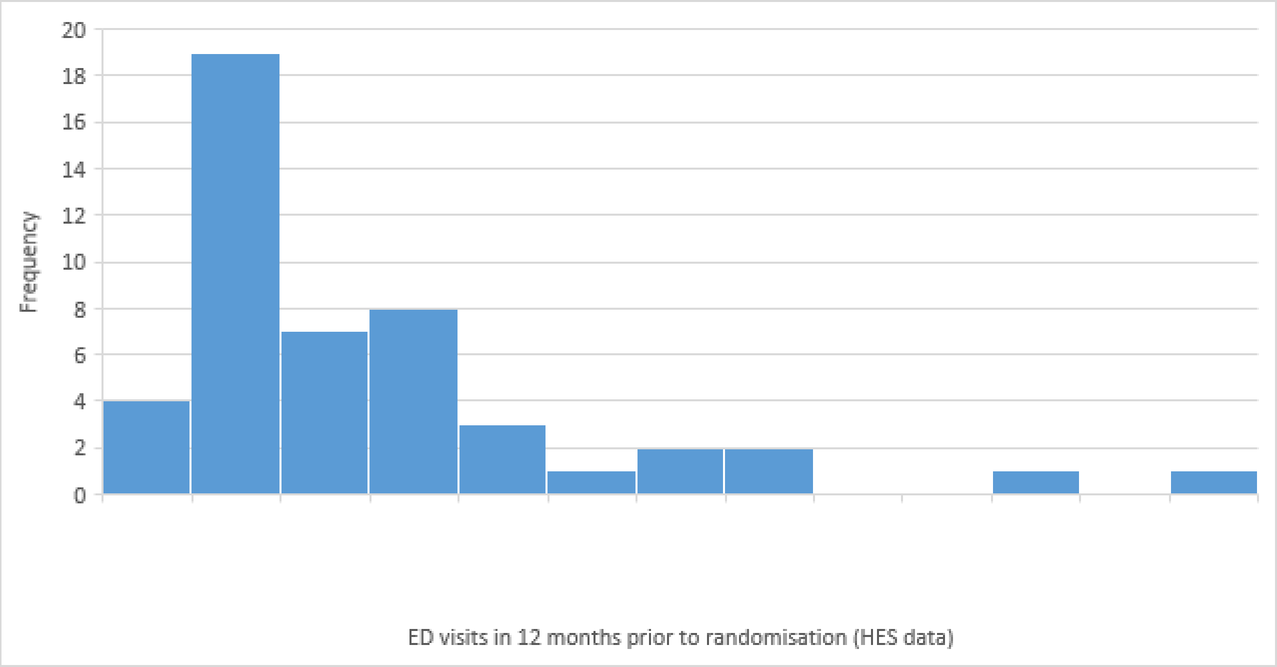

review only

Supplementary File 8 Summary of key milestones in securing ED outcome data from NHS Digital

Period	Project milestone		Milestone in obtaining data from NHS Digital	
	Date	*Note*	*Date*	*Note*
**Period**			**28 Sep 2015**	Received feedback on draft Participant Information Sheets and Consent form for trial to ensure compliance.
Trial period	**19 May 2016**	First patient participant randomised		
			**11 Oct 2017**	Participated in NHS Digital training.
			**18 Oct 2017**	Registered as new user of application system.
			**07 Nov 2017**	Delays experienced. Access to application form granted after chasing.
	**Dec 2017**	Final 12-month follow-up completed		
Post -trial			**16 Feb 2018**	Application submitted to NHS Digital
			**28 Feb 2018**	Teleconference with NHS Digital. Provisional opinion given that only minor changes required and likely timeframe to receipt of data 1.5 months.
			**07 Mar 2018**	Received written feedback from NHS Digital after chasing on minor changes required.
			**22 Mar 2018**	Revised application submitted.
			**03 Apr 2018**	NHS Digital submit new query (1) to applicants requesting additional information.<br>(Applicants respond 03 Apr 2018)
			**26 Apr 2018**	NHS Digital submit new query (2) to applicants requesting additional information.<br>(Applicants respond 26 Apr 2018)
			**30 Apr 2018**	NHS Digital submit new query (3) to applicants requesting additional information.<br>(Applicants respond 30 Apr 2018)
			**05 May 2018**	NHS Digital submit new query (4) to applicants requesting additional information.<br>(Applicants respond 05 May 2018).
			**10 May 2018**	NHS Digital submit new query (5) to applicants requesting additional information (in light of new General Data Protection Regulation introduced on May 2018).<br>(Applicants respond 11 May 2018)
			**30 May 2018**	NHS Digital submit new query (6) to applicants requesting additional information (in light of new General Data Protection Regulation introduced on May 2018).<br>(Applicants respond 30 May 2018)
			**30 May 2018**	NHS Digital submit new query (7) to applicants requesting additional information.<br>(Applicants respond 30 May 2018)
			**15 Jun 2018**	NHS Digital confirm Data Approvals Owner will review revised application.
			**27 Jun 2018**	NHS Digital submit new query (8) to applicants requesting additional information<br>and request revisions to trial website in light of new General Data Protection Regulation introduced on May 2018).<br>(Applicants respond 28 Jun 2018)

			**02 Jul 2018**	NHS Digital submit new query (9) to applicants requesting additional information.<br>(Applicants 02 Jul 2018)
			**06 Jul 2018**	NHS Digital notify applicants that Data Approval Owner has rejected application, primarily because: “concerns [over] whether this pilot would yield findings that were statistically valuable to achieve the stated aims given the small numbers”.
			**10 Jul 2018**	Secured confirmation of right to appeal and process.
			**20 Jul 2018**	Applicants submit letter of appeal.
			**25 Jul 2018**	Appeal accepted by NHS Digital.
			**15 Aug 2018**	NHS Digital submit new query (10) to applicants following Independent Group Advising on the Release of Data (IGARD) committee’s review of application.<br>(Applicants respond 16 Aug 2018)
			**16 Aug 2018**	Teleconference with NHS Digital data production team.
			**04 Sep 2018**	NHS Digital send Data Sharing Agreement to applicants.
			**10 Sep 2018**	NHS Digital confirm receipt of completed Data Sharing Agreement.
			**25 Sep 2018**	Applicants securely transfer patient participants’ details to NHS Digital.
			**31 Sep 2018**	NHS Digital release data to applicants.

Supplementary File 9 Adverse events occurring during pilot trial in descending order according to frequency overall [a]

**Adverse event**		**SAFE + TAU**		**TAU**		**Total**	
Category of events (e.g. body system)	Event	Events (n)	Patients [n(%)]	Events (n)	Patients [n(%)]	Events (n)	Patients [n(%)]
Eyes, ear, nose, throat	Problem with eyes and sinuses	0	0 (0.0%)	1	1 (9.1%)	1	1 (5.6%)
Genito-urinary	Overnight hospital admission required due to urinary tract infection (pre existing)	0	0 (0.0%)	1	1 (9.1%)	1	1 (5.6%)
Haematological	Dislocated shoulder	0	0 (0.0%)	1	1 (9.1%)	1	1 (5.6%)
Neoplasia	Change in seizure pattern [b]	1	1 (14.3%)	0	0 (0.0%)	1	1 (5.6%)
	Increase in seizure frequency [b]	1	1 (14.3%)	0	0 (0.0%)	1	1 (5.6%)
Neurological	Increased seizures, medications changed	1	1 (14.3%)	3	3 (27.3%)	4	4 (22.2%)
	Increased seizures (shift patterns at work)	1	1 (14.3%)	0	0 (0.0%)	1	1 (5.6%)
	New seizure type; frequent absence seizures as well as usual seizure types	1	1 (14.3%)	0	0 (0.0%)	1	1 (5.6%)
	Seizure frequency sodium levels requiring in patient monitoring	0	0 (0.0%)	1	1 (9.1%)	1	1 (5.6%)
	Seizures more severe (tonic clonic)	0	0 (0.0%)	1	1 (9.1%)	1	1 (5.6%)
	Vagus Nerve Stimulation not working properly, going to hospital for observation	0	0 (0.0%)	1	1 (9.1%)	1	1 (5.6%)
Respiratory	Increases number of seizures due to chest infection and antibiotics	0	0 (0.0%)	1	1 (9.1%)	1	1 (5.6%)
Other:	Gynae-pregnancy. Increase in the number of fits due to being pregnant / just given birth	0	0 (0.0%)	2	1 (9.1%)	2	1 (5.6%)
	Diagnosis of status epilepticus	1	1 (14.3%)	0	0 (0.0%)	1	1 (5.6%)
	Vertigo	0	0 (0.0%)	1	1 (9.1%)	1	1 (5.6%)
**Total**		6	6 (100.0%)	13	11 (100.0%)	19	18 (100.0%)

Notes:

[a] Eighteen (35.3%) patient participants reported adverse events (AE) over the course of follow-up. In the SAFE+TAU group, 6 AEs were reported by 6 participants, while 13 AEs were reported by 12 participants from the TAU group. Only one SAE occurred during the study – namely, a diagnosis of previous status epilepticus. This was reported by one SAFE+TAU group participant. A medical review asserted it unlikely to be related to participation as it arose from investigations the participant had been having before participation.

[b] Two participants were under investigation to determine whether a brain tumour could be the cause of their seizure changes; hence 'Body System' is classified as 'Neoplasia' rather than 'Neurological.'

For peer review only

Supplementary File 10 Total observed costs and cost per delegate to deliver the seizure first aid intervention (SAFE) within a research trial with two academic researchers, one Epilepsy Specialist Nurse delivering the training, and administrative support.

**Description**	**Design & set-up**	**Practice Session 1**	**Practice Session 2**	**SAFE+TAU Group 1**	**SAFE+TAU Group 2**	**SAFE+TAU Group 3**	**SAFE+TAU Group 4**	**SAFE+TAU Group 5**	**SAFE+TAU Group 6**	**SAFE+TAU Group 7**	**TAU Group 1**	**TAU Group 2**
Fixed costs (£)												
Equipment	928.85	0.00	0.00	0.00	0.00	0.00	0.00	0.00	0.00	0.00	0.00	0.00
Website	77.12	0.00	0.00	0.00	0.00	0.00	0.00	0.00	0.00	0.00	0.00	0.00
Freepost licence	116.40	0.00	0.00	0.00	0.00	0.00	0.00	0.00	0.00	0.00	0.00	0.00
Venue	0.00	80.00	80.00	80.00	80.00	80.00	80.00	80.00	80.00	80.00	80.00	80.00
Facilitator (staff cost)		192.79	192.79	177.96	177.96	177.96	177.96	177.96	177.96	177.96	177.96	177.96
Facilitation resources		4.96	4.96	4.96	4.96	4.96	4.96	4.96	4.96	4.96	4.96	4.96
Total (fixed)	1122.37	277.75	277.75	262.92	262.92	262.92	262.92	262.92	262.92	262.92	262.92	262.92
Variable costs (£)												
Staff costs	4243.41	612.88	1158.56	591.19	521.84	429.37	429.37	318.41	318.41	318.41	299.96	416.03
Staff travel expenses	44.10	279.87	279.87	274.67	274.67	265.43	265.43	265.43	265.43	265.43	233.63	265.41
Participant expenses	0.00	180.00	200.00	40.00	40.00	40.00	40.00	40.00	40.00	40.00	92.80	0.00
Office costs (inc. telephone)	589.33	33.00	105.50	81.63	85.23	92.60	88.70	89.00	86.60	89.00	57.17	70.13
Stationery, print, post	0.00	156.35	196.96	57.44	93.68	68.93	68.93	34.46	22.98	45.95	182.73	298.44
Refreshments	0.00	72.25	72.25	41.80	41.80	32.81	32.00	41.00	26.60	18.50	21.00	34.50
Advertising	45.00	0.00	0.00	0.00	0.00	0.00	0.00	0.00	0.00	0.00	0.00	0.00
Total (variable)	4921.84	1334.34	2013.13	1086.73	1057.22	929.13	924.42	788.30	760.01	777.29	887.28	1084.51
TOTAL COST (£)	6044.21	1612.09	2290.88	1349.65	1320.14	1192.05	1187.34	1051.22	1022.93	1040.21	1150.20	1347.43
Delegate (n) [patient:SO]		12	15	2:3	4:4	3:3	4:2	3:0	1:1	2:2	4:4	8:5

For peer review only

Mean cost/delegate (£)		134.34	152.73	269.93	165.02	198.68	197.89	350.41	511.47	260.05	143.78	103.65
Mean cost per group (sd)<br>95% central range *				£1,166 (134.22)<br>£1,023-£1,350							£1,249 (139.46)<br>£1,150-£1,347	
Mean cost per delegate<br>95% central range*				£240<br>£211-£278							£119<br>£110-£128	
Mean cost per patient (with or without SO)<br>95% central range*				£408<br>£358-£472							£208<br>£192-£225	

Notes:

*Central ranges generated from 10,000 bootstrap replications; The quantity of use of the resources, was measured. To capture the resources used the 'time and motion method' was used with AN, DS, JB and GM completing electronic data collection forms on which they self-reported the time they dedicated to different activities. The research team (AN, DS) also recorded details of the number of participants invited and attending each SAFE sessions and non-staff resources.

For peer review only

CONSORT 2010 checklist of information to include when reporting a pilot or feasibility trial*

Section/Topic	Item No	Checklist item	Reported on page No
**Title and abstract**			
	1a	Identification as a pilot or feasibility randomised trial in the title	1
	1b	Structured summary of pilot trial design, methods, results, and conclusions (for specific guidance see CONSORT abstract extension for pilot trials)	2
**Introduction**			
Background and objectives	2a	Scientific background and explanation of rationale for future definitive trial, and reasons for randomised pilot trial	4-5
	2b	Specific objectives or research questions for pilot trial	5
**Methods**			
Trial design	3a	Description of pilot trial design (such as parallel, factorial) including allocation ratio	6 (sec. 2.2), 7 (sec.2.6)
	3b	Important changes to methods after pilot trial commencement (such as eligibility criteria), with reasons	6 (sec. 2.2)
Participants	4a	Eligibility criteria for participants	6-7 (sec. 2.2)
	4b	Settings and locations where the data were collected	6 (sec. 2.2)
	4c	How participants were identified and consented	6 (sec. 2.4) & Supplementary File 1
Interventions	5	The interventions for each group with sufficient details to allow replication, including how and when they were actually administered	7 (sec. 2.8), Table 1, and references 32 & 37.
Outcomes	6a	Completely defined prespecified assessments or measurements to address each pilot trial objective specified in 2b, including how and when they were assessed	7-8 (sec. 2.9)
	6b	Any changes to pilot trial assessments or measurements after the pilot trial commenced, with reasons	N/A.
	6c	If applicable, prespecified criteria used to judge whether, or how, to proceed with future definitive trial	7 (sec. 2.9)
Sample size	7a	Rationale for numbers in the pilot trial	9 (sec. 2.10)
	7b	When applicable, explanation of any interim analyses and stopping guidelines	N/A.
Randomisation:			

For peer review only

Sequence generation	8a	Method used to generate the random allocation sequence	7 (sec.2.6)
	8b	Type of randomisation(s); details of any restriction (such as blocking and block size)	7 (sec.2.6)
Allocation concealment mechanism	9	Mechanism used to implement the random allocation sequence (such as sequentially numbered containers), describing any steps taken to conceal the sequence until interventions were assigned	7 (sec.2.6)
Implementation	10	Who generated the random allocation sequence, who enrolled participants, and who assigned participants to interventions	7 (sec.2.6)
Blinding	11a	If done, who was blinded after assignment to interventions (for example, participants, care providers, those assessing outcomes) and how	7 (sec.2.6)
	11b	If relevant, description of the similarity of interventions	N/A.
Statistical methods	12	Methods used to address each pilot trial objective whether qualitative or quantitative	8-9 (sec. 2.10)
**Results**			
Participant flow (a diagram is strongly recommended)	13a	For each group, the numbers of participants who were approached and/or assessed for eligibility, randomly assigned, received intended treatment, and were assessed for each objective	9-10 (sec. 3.1), 10-11 (sec. 3.3), Figure 1
	13b	For each group, losses and exclusions after randomisation, together with reasons	10 (3.1), Figure 1
Recruitment	14a	Dates defining the periods of recruitment and follow-up	6 (sec. 2.1, 2.2)
	14b	Why the pilot trial ended or was stopped	N/A.
Baseline data	15	A table showing baseline demographic and clinical characteristics for each group	Table 4
Numbers analysed	16	For each objective, number of participants (denominator) included in each analysis. If relevant, these numbers should be by randomised group	Tables 4, 5, 6 and Supplementary File
Outcomes and estimation	17	For each objective, results including expressions of uncertainty (such as 95% confidence interval) for any estimates. If relevant, these results should be by randomised group	9-13 (sec. 3)
Ancillary analyses	18	Results of any other analyses performed that could be used to inform the future definitive trial	Supplementary Files
Harms	19	All important harms or unintended effects in each group (for specific guidance see CONSORT for harms)	12 (3.7), Supplementary File 9
	19a	If relevant, other important unintended consequences	N/A
**Discussion**			

For peer review only

Limitations	20	Pilot trial limitations, addressing sources of potential bias and remaining uncertainty about feasibility	16 (sec. 4.4)
Generalisability	21	Generalisability (applicability) of pilot trial methods and findings to future definitive trial and other studies	16 (sec. 4.4)
Interpretation	22	Interpretation consistent with pilot trial objectives and findings, balancing potential benefits and harms, and considering other relevant evidence	13-15 (sec. 4)
	22a	Implications for progression from pilot to future definitive trial, including any proposed amendments	15 (sec. 4.3)
**Other information**			
Registration	23	Registration number for pilot trial and name of trial registry	1
Protocol	24	Where the pilot trial protocol can be accessed, if available	6 (reference 34)
Funding	25	Sources of funding and other support (such as supply of drugs), role of funders	17
	26	Ethical approval or approval by research review committee, confirmed with reference number	6 (sec. 2.3)

Citation: Eldridge SM, Chan CL, Campbell MJ, Bond CM, Hopewell S, Thabane L, et al. CONSORT 2010 statement: extension to randomised pilot and feasibility trials. BMJ. 2016;355.
*We strongly recommend reading this statement in conjunction with the CONSORT 2010, extension to randomised pilot and feasibility trials, Explanation and Elaboration for important clarifications on all the items. If relevant, we also recommend reading CONSORT extensions for cluster randomised trials, non-inferiority and equivalence trials, non-pharmacological treatments, herbal interventions, and pragmatic trials. Additional extensions are forthcoming: for those and for up to date references relevant to this checklist, see www.consort-statement.org.

For peer review only

BMJ Open

Seizure First Aid Training For people with Epilepsy (SAFE) frequently attending emergency departments and their significant others: results of a UK multi-centre randomised controlled pilot trial

Journal:	*BMJ Open*
Manuscript ID	bmjopen-2019-035516.R1
Article Type:	Original research
Date Submitted by the Author:	29-Jan-2020
Complete List of Authors:	Noble, Adam; University of Liverpool, Department of Health Services Research<br>Snape, Dee; University of Liverpool, Department of Health Services Research<br>Nevitt, Sarah; University of Liverpool, Department of Biostatistics<br>Holmes, EA; Centre for Health Economics & Medicines Evaluation<br>Morgan, Myfanwy; King's College London, Institute of Pharmaceutical Science<br>Tudur-Smith, Catrin; University of Liverpool, Biostatistics<br>Hughes, Dyfrig; Bangor University, Centre for Health Economics and Medicines Evaluation<br>Buchanan, Mark; Wirral University Teaching Hospital NHS Foundation Trust, Emergency Department, Arrowe Park Hospital<br>McVicar, Jane; Mackinnon Memorial Hospital, NHS Highland,<br>MacCallum, Elizabeth; Aintree University Hospital, Liverpool University Hospitals NHS Foundation Trust, Emergency Department<br>Goodacre, Steve; University of Sheffield, Medical Care Research Unit<br>Ridsdale, Leone; King's College London, Department of Basic and Clinical Neuroscience, Institute of Psychiatry, Psychology & Neuroscience<br>Marson, Anthony; University of Liverpool, Molecular and Clinical Pharmacology
**Primary Subject Heading**:	Neurology
Secondary Subject Heading:	Health services research, Emergency medicine, Health economics
Keywords:	Epilepsy < NEUROLOGY, ACCIDENT & EMERGENCY MEDICINE, Organisation of health services < HEALTH SERVICES ADMINISTRATION & MANAGEMENT, Health economics < HEALTH SERVICES ADMINISTRATION & MANAGEMENT, Clinical trials < THERAPEUTICS

SCHOLARONE™
Manuscripts

I, the Submitting Author has the right to grant and does grant on behalf of all authors of the Work (as defined in the below author licence), an exclusive licence and/or a non-exclusive licence for contributions from authors who are: i) UK Crown employees; ii) where BMJ has agreed a CC-BY licence shall apply, and/or iii) in accordance with the terms applicable for US Federal Government officers or employees acting as part of their official duties; on a worldwide, perpetual, irrevocable, royalty-free basis to BMJ Publishing Group Ltd ("BMJ") its licensees and where the relevant Journal is co-owned by BMJ to the co-owners of the Journal, to publish the Work in this journal and any other BMJ products and to exploit all rights, as set out in our licence.

The Submitting Author accepts and understands that any supply made under these terms is made by BMJ to the Submitting Author unless you are acting as an employee on behalf of your employer or a postgraduate student of an affiliated institution which is paying any applicable article publishing charge ("APC") for Open Access articles. Where the Submitting Author wishes to make the Work available on an Open Access basis (and intends to pay the relevant APC), the terms of reuse of such Open Access shall be governed by a Creative Commons licence – details of these licences and which Creative Commons licence will apply to this Work are set out in our licence referred to above.

Other than as permitted in any relevant BMJ Author's Self Archiving Policies, I confirm this Work has not been accepted for publication elsewhere, is not being considered for publication elsewhere and does not duplicate material already published. I confirm all authors consent to publication of this Work and authorise the granting of this licence.

For peer review only

TITLE: Seizure First Aid Training For people with Epilepsy (SAFE) frequently attending emergency departments and their significant others: results of a UK multi-centre randomised controlled pilot trial

AUTHORS: A.J. Noble,[1*] D Snape[1], S Nevitt,[2] EAF Holmes,[3] M Morgan,[4] C Tudur Smith,[2] DA Hughes,[3,10] M Buchanan,[5] J McVicar,[6] E MacCallum,[7] S Goodacre,[8] L Ridsdale, [9] AG Marson.[10]

AFFILIATIONS:

[1] Department of Health Services Research, University of Liverpool, Liverpool, UK

[2] Liverpool Clinical Trials Centre, Department of Biostatistics, University of Liverpool, Liverpool, UK

[3] Centre for Health Economics and Medicine Evaluation, Bangor University, Bangor, UK

[4] Institute of Pharmaceutical Science, King's College London, London, UK

[5] Emergency Department, Arrowe Park Hospital, Wirral University Teaching Hospital NHS Foundation Trust, Wirral, UK.

[6] Mackinnon Memorial Hospital, Broadford, NHS Highland, Isle of Skye, UK.

[7] Emergency Department, Aintree University Hospital, Liverpool University Hospitals NHS Foundation Trust

[8] School of Health and Related Research, Sheffield University, Sheffield, UK

[9] Department of Basic and Clinical Neuroscience, King's College London, London, UK

[10] Department of Molecular and Clinical Pharmacology, University of Liverpool, Liverpool, UK

FOR CORRESPONDENCE: Name: Adam Noble PhD; Address: University of Liverpool, Department of Health Services Research, Waterhouse Building, Liverpool, L69 3GB, United Kingdom.; Telephone: +44 151 794 5993; E-mail: adam.noble@liverpool.ac.uk

For peer review only

For peer review only - http://bmjopen.bmj.com/site/about/guidelines.xhtml

ABSTRACT

Objective: To determine the feasibility and optimal design of a randomised controlled trial (RCT) of Seizure First Aid Training For Epilepsy (SAFE).

Design: Pilot RCT with embedded microcosting.

Setting: Three English hospital emergency departments (EDs).

Participants: Patients aged ≥16 with established epilepsy reporting ≥2 ED visits in the prior 12 months and their significant others (SO).

Interventions: Patients (and their SO) were randomly allocated (1:1) to SAFE plus Treatment-As-Usual (TAU) or TAU alone. SAFE is a 4-hour group course.

Main outcome measures: Two criteria evaluated a definitive RCT's feasibility: i) ≥20% of eligible patients needed to be consented into the pilot; ii) routine data on use of ED over the 12-months post-randomisation needed securing for ≥75%. Other measures included: eligibility; ease of obtaining routine data; availability of self-report ED data and comparability; SAFE's effect; and intervention cost.

Results: Of ED attendees with a suspected seizure, 424 (10.6%) were eligible; 53 (12.5%) patients and 38 SOs consented. Fifty-one patients (and 37 SOs) were randomised. Routine data on ED use at 12-months was secured for 94.1%. Self-report ED data was available for 66.7%. Patients reported more visits compared to routine data. Most (76.9%) randomised to SAFE received it and no related serious adverse events occurred. ED use at 12 months was lower in the SAFE+TAU arm compared to TAU alone, but not significantly (Rate Ratio= 0.62, 95% confidence interval 0.33 to 1.17). A definitive trial would need ~674 patient participants and ~39 recruitment sites. Obtaining routine data was challenging, taking ~8.5 months.

Conclusions: In satisfying only one predetermined 'stop/go' criterion, a definitive RCT is not feasible. The low consent rate in the pilot raises concerns about a definitive trial's findings external validity and means it would be expensive to conduct. Research is required into how to optimise recruitment from the target population.

Trial registration number: ISRCTN13 871 327.

For peer review only - http://bmjopen.bmj.com/site/about/guidelines.xhtml

'Strengths and limitations of this study' (5 bullet points; one sentence each, relating to the methods)

1. Randomisation was done remotely by computer and stratification factors and allocations concealed from those collecting baseline and follow-up data and analysing it.
2. Participants were recruited from sites serving areas where social deprivation was high and epilepsy control poor and so similar to those where a definitive RCT would likely need to focus recruitment.
3. We completed one of the few microcostings of a self-management intervention for epilepsy.
4. Despite recruited patient participants stating sufficient ED use when screened, routine data subsequently suggested ~40% did not meet the inclusion criteria.
5. We estimated the effect of SAFE only on the proposed primary outcome measure for a definitive RCT.

KEY WORDS: Epilepsy; Accident & Emergency Medicine; Organisation of health services; Heath economics; Clinical Trials.

Words: 3,955

.

For peer review only

1. BACKGROUND

International evidence shows a significant minority of people with epilepsy (PWE) frequently use hospital emergency departments (EDs).[1-4] In the United Kingdom (UK), around 20% of PWE visit each year, half are admitted [5-7]. Whilst the exact distribution of use amongst attendees is unclear,[8-10] ~60% may make several visits each year.[11]

Emergency hospital use by PWE has been identified as an area for potential cost savings [12] as whilst expensive – ~£70-90M in England each year [8 13 14] – it is often of little clinical benefit since most attendees have known, rather than new epilepsy and experienced uncomplicated seizures.[9 15-17] Guidance states such seizures could be managed by PWE and their significant others (SOs).[18 19] Indeed, iatrogenic harms may arise in seeking emergency care for them.

PWE visiting EDs have a characteristic profile. They report more seizures, anxiety, poorer quality of life and are more likely to live in socially deprived areas compared to the wider epilepsy population.[11 20-23] They therefore share some of the characteristics of those at increased risk of epilepsy associated death.[24 25] In the UK, PWE visiting EDs can also be challenging to identify for research since most (~62%) are not being followed-up by an epilepsy specialist,[1] primary care providers are also not always notified of ED visits by their patients [9] and because EDs do not always code the reason for an attendance.[26]

Whilst the focus on PWE attending EDs is welcomed, it needs to be translated into care improvements. Although a range of promising interventions have been suggested,[27] assessment of their utility is lacking.[28]

One intervention proposed is seizure first aid training. It has potential as PWE frequently visiting EDs and their SOs (to whom care decisions can be delegated [29 30]) express particularly low knowledge and confidence in this domain, are fearful of seizures, and there are indications this leads them to seek emergency medical attention for seizures, rather than self-managing them.[31]

As no such intervention existed, we developed one – called Seizure First Aid Training For Epilepsy (SAFE). SAFE is a manualised group-based self-management course

For peer review only - http://bmjopen.bmj.com/site/about/guidelines.xhtml

(Table 1). It's rationale and development has been reported.[32] In brief, it was co-designed with health professionals, patients and carers. It was developed for delivery to groups of up to 10 patient-carer dyads by a single facilitator with knowledge of epilepsy and lasts for ~4 hours. It contains 6 modules centred around basic epilepsy and first aid knowledge, the recovery position, informing others about epilepsy and how to help if seizures occur, medical identification, seizure triggers and home safety. Materials include presentation slides and professionally produced videos. Its behaviour change potential has been optimised by course recipients completing a Self-Affirmation exercise at the start.

SAFE's efficacy now needs testing. A randomized controlled trial (RCT) would be an appropriate methodology for this. ED use over the 12 months following randomisation could be the primary outcome. It is unknown though whether such a trial is feasible and what its optimal design should be.

Specifically, will patients and their SOs take part, attend SAFE, and be willing and able to be followed-up? It is also not possible to calculate the required sample size because the distribution of ED use is unclear. These uncertainties exist because SAFE is newly developed and no RCT has attempted to recruit the target population.

It is also unclear how to measure ED visits. Funders are encouraging trialists to use routinely recorded data to assess outcomes. In England, routine data on a person's ED use is recorded within the Hospital Episode Statistics system. This data's cost, how long it takes to obtain and its comparability to self-report is though unknown.

In such circumstances, guidelines highlight the importance of pilots.[33] Their primary focus is not effect, but judging feasibility and providing information to optimise a definitive RCT's design.[34] We thus completed a pilot RCT comparing SAFE, plus treatment-As-Usual (TAU) to TAU alone. It had the following objectives, which will be relevant to those interested in SAFE and those researching this population:

i) Estimate likely eligibility, consent, and retention rates in a definitive RCT;

For peer review only - http://bmjopen.bmj.com/site/about/guidelines.xhtml

ii) Estimate annual rate of ED visits in TAU group and the likely dispersion parameter;
iii) Determine feasibility of measuring ED use by routine data;
iv) Estimate completion rates of study assessment tools;
v) Estimate rates of researcher unblinding;
vi) Provide summary statistics to estimate effect of SAFE on ED use and its precision;
vii) Capture patient and SOs views on trial participation;
viii) Estimate the intervention's cost.

2. METHODS

The trial's protocol has been published.[31] Here we provide a brief overview.

2.1 Design

Parallel arm, multicentre, external pilot RCT. Assessments with participants on the definitive trial's proposed primary and secondary outcome measures were performed at baseline prior to randomization (T0) and 12-months later (T3) (Table 2). Interim assessments occurred at 3 (T1) and 6-months (T2).

SAFE was offered to the TAU alone group after T3 assessments were completed.

2.2 Trial setting

Three hospitals in North-West England – which serve populations featuring high social deprivation [35 36] and emergency admissions for epilepsy [22] – were recruitment sites (Acknowledgements). From May to December 2016 patients were invited who had attended any of these hospital's ED over the prior 12 (and with governance approval, later extended to 18) months for epilepsy (see 2.4).

2.3 Ethical considerations and approvals

The National Research Ethics Service Committee (15/NW/0225) and Health Research Authority (166241) approved the study. An independent Trial Steering Committee monitored it.

2.4 Patient recruitment

For peer review only - http://bmjopen.bmj.com/site/about/guidelines.xhtml

ED clinicians searched their hospitals' attendance record systems for potential participants (Supplementary File 1), screened their triage cards and posted invitations to eligible patients. Recipients had 3 weeks to return an opt-out response if not interested in participating. Those not opting-out were telephoned by a researcher to verify eligibility and willingness to participate. Patients taking part (and their SO if they chose to take part with one) provided informed consent at an enrolment appointment with a researcher (DS). For patients, this included consent to access their routine data.

2.5 Eligibility criteria

Table 3 details the criteria. In brief, patients were eligible if they were aged ≥16, diagnosed with epilepsy, prescribed antiepileptic drugs, could give informed consent and, when telephoned, self-reported ≥2 ED visits for epilepsy in the prior 12 months.

2.6 Randomization and blinding

Patients (and their SOs) were randomised (1:1) by an online system managed by the Liverpool Clinical Trials Centre. It used a minimisation program with a built-in random element and two stratification factors (recruitment site and whether the patient reported epilepsy stigma at baseline).

Usual care provider(s) and DS, who was responsible for recruitment and follow-up, were blinded to allocations and stratification factors. Participants could not be blinded and so were asked not to reveal their allocation to DS.

Staff (GM) organizing attendance at SAFE were not involved in data collection and not blinded. The trial (SN) statistician and senior statistician (CTS) were blinded until the database was 'locked'.

2.7 Measures to assess patient and SOs participants' outcomes

Table 2 details these measures.

2.8 Intervention

An epilepsy nurse specialist (JB) delivered SAFE within a local hospital's educational centre. A fidelity assessment found they delivered SAFE with excellent protocol adherence and competence.[37]

For peer review only - http://bmjopen.bmj.com/site/about/guidelines.xhtml

2.9 Outcomes

To achieve the study objectives, rates of eligibility, consent and retention were calculated. Retention being the percentage of patients for which 12-month primary outcome data was secured. Two a priori progression criteria helped judge feasibility: i) ≥20% of eligible patients were recruited; ii) 12-month primary outcome data secured for ≥75% of patients.

By assessing participants on the proposed outcome measures, we obtained estimates of the distribution of ED use, measure completeness and SAFE's effect. To evaluate blinding, DS completed a 'Treatment Guess' form after each participant's T3 assessment or withdrawal. It required her to state which treatment arm she believed the participant had been randomised to. The proportion of participants willing to participate in such a trial again was ascertained and experience of adverse events calculated. Time taken to obtain routine data and the fee payable was recorded. To see if self-reported ED visits provided a reliable estimate compared to routine data the agreement between the two data sources on how many ED visits a patient had made was explored.

2.10 Statistical analyses

As this was a pilot RCT, a power calculation was inappropriate. Instead, the sample size was chosen to provide adequate precision to estimate the parameters required.[38]

Based on existing data,[1 14 17 39 40] it was anticipated 12 months of attendances from each ED would allow identification of ~400 eligible patients. With a 20% consent rate, 80 patient participant accruals could be expected. With 80 patient participants we could estimate a potential drop-out rate of 25% to within a 95% confidence interval of +/-10% and a consent rate of 20% to within a 95% confidence interval of +/-4%. Assuming ED data at T3 was not available for 25% of patients, data from 60 patients would still allow robust estimation of the ED rate and dispersion parameter. Sample sizes of 24 to 50 have been recommended as 'adequate' for pilot studies.[38 41]

Analyses were documented a priori in an analysis plan and performed using SAS (9.4). Baseline characteristics for patient and SO participants are described using

For peer review only - http://bmjopen.bmj.com/site/about/guidelines.xhtml

descriptive statistics and patient participants compared, on a subset, to eligible patients declining participation. Parameters are reported with 95% confidence intervals (CIs).

SAFE's effect on ED use, with and without adjustment for number of ED visits prior to randomisation, was estimated on a modified intention-to-treat basis, including participants with their number of ED visits recorded, with no data imputation, using negative binomial regression (NBR). NBR was the pre-specified statistical approach as over-dispersion (i.e. large variance) was anticipated in the number of ED visits reported. Between-group differences are presented as Rate Ratios and, as per recommendations for hypothesis testing within pilot trials,[42] tested according to 5, 10 and 20% levels of significance.

The proportion of correct treatment guesses was determined and Cohen's Kappa computed. Bland-Altman plots compared ED visits as measured by routine data and self-report.[43]

As no consensus exists about what constitutes a clinically important reduction in ED visits,[44] average annual rate of ED visits in the SAFE+TAU and TAU alone groups post randomisation (measured according to routine data) and the likely dispersion parameter from the adjusted NBR model were used to estimate the sample size of a definitive trial using Keene et al.'s [45] formula. According to the formula, the number of patient participants required per group in a definitive trial to detect the size of the effect shown in the pilot study is:

$$n = \left\{ \frac{z_{1-\beta} + z_{1-\alpha/2}}{\log(\mu_1/\mu_2)} \right\}^2 \times \left\{ \frac{\mu_1 + \mu_2}{\mu_1 \mu_2} + 2k \right\}$$

Where $Z_{1-\alpha/2}$ and $Z_{1-\beta}$ are critical values of the Normal distribution for specific values of alpha (α) and power (β). μ_1 and μ_2 are the estimated ED rates from the two treatment groups and k the negative binomial shape parameter from the associated gamma distribution which explicitly represents variability between subjects. For the calculations, alpha was set at 5%, but the dispersion parameter and power required was varied to explore differences in sample size required.

For peer review only - http://bmjopen.bmj.com/site/about/guidelines.xhtml

2.11 Microcosting

Microcosting adopted the perspective of an academic non-profit making institution and conducted in three steps:[46] (1) resource identification; (2) resource use measurement, applying the time and motion method;[47] and (3) valuation using price year 2017/18 for local and national data. Data were analysed using Microsoft Excel to calculate the fixed and variable costs of delivering SAFE. Fixed costs included, equipment, website, freepost licence, venue hire, facilitator staff cost, and facilitation resources; and assumed 11 groups/year and equipment life of 1-year. Variable costs were support staff and office costs, staff and participant travel expenses, and consumables. Total cost per group was calculated as fixed costs plus variable costs, for each group and each arm (SAFE+TAU; TAU). Mean cost per group was calculated as the sum of total costs / number of groups. Mean cost per delegate (or patient only) were calculated as sum of total cost per group in each arm / sum of delegates (or patients only) in each arm. Results are presented as: cost per training session, mean cost per delegate, mean cost per patient. The 95% central range (CR) for costs and differences were generated using Monte Carlo Simulation of 10,000 replications.

2.12 Patient and Public Involvement statement

This research came about as improving education for patients and families on epilepsy is a top research priority.[48] To ensure SAFE was developed and tested in a way that met service users' needs: the 'Epilepsy Society' and patient and SO representatives from the 'Brain and Spine Foundation' helped develop recruitment materials; the pilot was overseen by a Trial Steering Committee including 2 service user representatives; SAFE's was designed by an Intervention Panel including 2 service user representatives and its content informed by feedback from 23 service users;[32] finally, pilot trial participants reported on the burden of participation with a view to optimising the design of a potential definitive RCT.

3. RESULTS

3.1 Participant recruitment, allocation and treatment

Of the 4016 individuals identified, 1220 (30.4%, CI 29.0% to 31.8%) had visited for established epilepsy. Of these, 424 (34.8%, CI 32.1% to 37.4%) were eligible; eligibility rate 10.6% (CI 9.6% to 11.5%) (Figure 1 and Supplementary File 2).

For peer review only - http://bmjopen.bmj.com/site/about/guidelines.xhtml

Of the 424 patients invited, 53 consented; consent rate 12.5% (CI 9.3% to 15.6%). Telephone contact could though only be made with 203 (47.8%, CI 43.1% to 52.6%) patients. The consent rate amongst those who could be contacted was 26.1% (CI 20.0% to 32.2%). The main reasons for 150 patients declining participation were “lack of interest” (42.7%, CI 34.8% to 50.6%) and being “too busy” (22.6%; CI 16.0% to 29.4%).

Of the 53 consenting patients, 51 were randomised (with 37 SOs) (Figure 1 gives reasons for non-randomisation). Of the 51, 26 (and 18 SOs) were allocated to SAFE+TAU and 25 (and 19 SOs) to TAU alone. Most (20, 76.9%) patients and SOs (13, 72.2%) randomized to SAFE+TAU attended a course.

3.2 Participant demographics and epilepsy characteristics

The patient participants mean age was 39.9 years (SD 15.7, range 16-71); 29 (56.9%) were female and most (74.4%) lived in areas high in deprivation (49% in the 10 percent most socially deprived areas in England) (Table 4).

Recruited patients were comparable in age and deprivation to those declining participation. Females were though over-represented (Table 4).

Recruited patients had an epilepsy diagnosis for a median of 17.3 years. Most (62.8%) reported ≥10 seizures in the previous year and having seen a neurologist (74.5%) (Table 4). Participants’ mean Quality of Life 31-P score was low at 48.3 (SD 17.3; range 17.1 to 79.5). Twenty-six (50.9%) had ‘probable’ clinical anxiety. Most (n=42, 82.3%) reported feeling stigmatised by epilepsy; 15 (29.4%) highly. The treatment groups were broadly similar in demographics and epilepsy characteristics.

SO participants were typically a partner/spouse (43.2%) and most (89.2%) had daily patient contact (Supplementary File 3). With a mean Zarit Caregiver burden score of 18.9 (SD=12.51), SOs typically reported ‘little or no burden’ [49] but anxiety was high [50]; 15 (40.5%) SOs had ‘probable’ clinical anxiety.

3.3 Participant retention

For peer review only - http://bmjopen.bmj.com/site/about/guidelines.xhtml

3.3.1 Proposed primary outcome measure:

Of the randomised patients, 3 (5.8%) formally withdrew over follow-up, removing access to their routine data. Primary outcome data on ED use at 12 months (and for the 12 months prior to randomisation) was available for the remaining 48 patients, giving a retention rate of 94.1%.

3.3.2 Proposed secondary outcome measures:

Thirty-seven (72.5%) randomised patients and 21 (56.8%) SOs attended their 12-months questionnaire appointment (T3). The extent to which measures were completed at these and the interim appointments varied (Supplementary File 4). Self-report data on ED use at T3 was obtained from only 34 patients, giving a retention rate on this measure of 66.7% patients.

3.4 ED use

3.4.1 Baseline, prior to randomisation:

Routine data for the 48 patients for whom consent was maintained showed they made 122 ED visits in the 12 months before randomisation (Supplementary File 5). The mean was 2.5 and median 2 (range 0 to 12). ED use was slightly higher for TAU participants (Table 5).

Despite only consenting patients who when telephoned reported ≥2 ED visits in the prior 12 months, routine data indicated 4 (8.3%) had not made any visits during the prior 12 months. A further 19 (39.6%) made only one.

3.4.2 At 12 months, effect of SAFE:

Compared to the 12-months prior to randomisation, mean ED use over follow-up reduced for the SAFE+TAU group from 2.1 visits to 1.8 (difference -0.3). For the TAU group, it increased from 3 visits to 3.4 (difference 0.4) (Table 5). Unadjusted NBR estimated the visit rate was 46% lower in the SAFE+TAU group compared to the TAU group (Rate Ratio= 0.54; Vuong's test z=-0.17, P=0.87). In the adjusted model, SAFE's effect reduced (Rate Ratio= 0.62). Between group differences were not significant at the 5% or 10% alpha level in either the unadjusted or adjusted model. The effect was significant in both at the 20% level (Table 6). The dispersion parameter under the adjusted NBR model was k=0.69 (CI 0.17 to 1.21).

For peer review only - http://bmjopen.bmj.com/site/about/guidelines.xhtml

3.5 Obtaining routine data and its correspondence with self-report

Routine data took 8.5 months to secure, arriving ~9 months after T3 assessments finished. The direct cost was £6960. Supplementary File 6 shows substantial work on behalf of the research team to obtain the data was required. In one instance, an appeal against a decision to reject the application – 5 months in – was necessary.

Routine data did not match patient self-report in most cases. Forty-two patients had self-report and routine data on ED use at baseline. Only 3 (7.1%) reported the same number of visits as their routine data indicated. Most (76.2%) reported more (by 3.8 visits on average) (Figure 2a). There was greater agreement between routine and self-report data at T3; 11 (32.4%) patients reported the same number of visits as their routine data (Figure 2b).

3.6 Blinding

The researcher correctly guessed which of the two treatment arms35 patient participants had been allocated to by the randomisation process; unblinding rate 68.6% (CI 54.1% to 80.9%). The chance-corrected kappa statistic of 0.37 (CI 0.12 to 0.63) equated to “fair” agreement.

3.7 Safety

No serious adverse events related to participation occurred (Supplementary File 7).

3.8 Participant feedback

Thirty-two (68.1%) patients and 20 (62.5%) SOs completed the T3 feedback questionnaire. All but one said they would participate in such a trial again; participants indicated they perceived benefits from SAFE.

3.9 Sample size calculation

Based on the estimated effect of SAFE (see 3.4.2), Table 7 shows the number of patient participants required *per* group for a definitive trial. If the central value in the estimate range for the dispersion parameter *k* of 0.69 is used and 90% power stipulated, then a total starting sample of 674 patient participants (i.e., [308*2]+58) would need to be recruited. This accounts for the 9.4% of recruited patients who (on

the basis of the pilot trial) would be anticipated to withdraw consent to access their routine ED data. In the pilot, 5 of the 53 patients recruited withdraw consent.

3.10 Microcosting

Delivering SAFE was estimated to cost £333 (CR £288 to £380) per patient (with or without a SO). When including the cost SAFE's development (£181 per person), the mean cost per *attendee*, based on all 55 participants in SAFE+TAU or TAU groups who ultimately attended a SAFE session, was £375 (CR £348 to £402) (Supplementary File 8). This reduced to £194 (CR £167 to £221) when excluding sunk costs. Staff time accounted for 50.01% of the cost of SAFE's delivery. SAFE's facilitator was not local to the trial area. Thus, an analysis of facilitator costs without travel expenses (time and cost), reduced the mean cost by 21.08% from £194 (CR £167 to £221) to £152 (CR £124 to £179) per delegate (£261 per patient with or without SO). The annual fixed cost of setting-up and running SAFE was £1122 (+£35.07 per patient, based on 32 patients/year attending with or without SO).

4. DISCUSSION

The pilot was successful, providing estimates of key parameters, including recruitment and retention. An informed decision regarding feasibility and optimal design of a definitive trial of SAFE can now be made.

4.1 Positives from pilot for feasibility and design of definitive trial

4.1.1 Identification, treatment and safety:

The pilot indicates it is possible to identify, consent, randomise and safely treat persons from the target population within a definitive trial. This was not a given since no RCT had focused recruitment on them.

4.1.2 Retention and measuring ED use:

By using routine data as the basis of the primary outcome measure, a definitive trial should not be affected by attrition. It permitted 12-month data to be secured for 94% of pilot participants. Despite having shorter-follow-ups (6 months), 151 definitive RCTs funded by the NIHR secured outcome data for 89% of patients.[51] The use of routine data in our pilot meant it satisfied the retention progression criterion. It would not have

For peer review only - http://bmjopen.bmj.com/site/about/guidelines.xhtml

been met if self-report data were relied on (only 67% of patients provided it). This leads us to recommend ED use be measured using routine data.

Another reason for the recommendation is its cost would be low compared to employing staff to obtain self-report data. Routine data would also prevent a definitive trial from exposure to apparent recall bias, with patients seemingly over reporting ED use (it has been asserted bias is not an issue for ‘‘big ticket’’ service items).[52 53]

Our pilot does though caution that provision of routine data is slow and not straightforward. It took 8.5 months to obtain. In principle, such applications should be processed within 2 months.[54] Data providers should attend to such issues as those funding and designing trials need confidence data can be secured and realistic estimates for when. It is also worth noting that some of the ED visits attributed to our participants may not have been epilepsy related. Since a diagnostic code is not recorded for ~35% of ED visits, we asked NHS Digital to inform us of any ED visits recorded for our participants.[26]

4.2 Negatives from pilot for feasibility and design of definitive trial

4.2.1 Consent:

Only a small proportion of identified patients were eligible (10.6%) and willing (12.5%) to take part, meaning the progression criterion that ≥20% of eligible patients agree to participate was not met.

The progression criterion was stated a priori because when uptake is so low there is the real possibility that those who are and are not taking part differ. We compared the age and deprivation of patients who did and did not take part and found no obvious differences. We do not know though whether they differed on other indices since access to non-participants' medical records was not ethically permissible. One indication the pilot might not have recruited a representative sample was the high proportion of patients seeing a neurologist in the prior 12 months (~75%). Evidence suggests this should be closer to 38%.[1]

The consent rate also raises questions over the likelihood of a definitive trial being funded since it would make it expensive. On average, each pilot site generated 17.6

For peer review only - http://bmjopen.bmj.com/site/about/guidelines.xhtml

patient accruals from 18 months of attendances. To achieve a sample size of ~674 PWE, a definitive trial could thus require ~39 sites (half of England's EDs). This is 2.5 times more sites than in recent NIHR HTA funded RCTs [51] which already had a mean cost of ~£1.3M.[55] A definitive RCT of SAFE might thus not represent acceptable value for money to funders.[56]

Epilepsy and its consequences (e.g., seizures, memory difficulties, no driving license) makes recruiting PWE challenging. The consent rate in the pilot is nevertheless low. In the largest RCT of self-management (SMILE trial), 37% of the people with uncontrolled epilepsy invited took part.[57] The characteristics of the patients from ED agreeing to participate in our pilot suggests one reason for the low uptake might be stigma; 82% felt some (21.6% felt highly stigmatised). This is higher than in the wider epilepsy population; 63% of SMILE's sample felt stigmatised (12.5% highly).[58] Stigma can make PWE feel ashamed and reluctant to talk about epilepsy. This could explain why the target population is so challenging to recruit. Unfortunately, it is unclear how to revise recruitment to mitigate against this.

Evidence-based strategies were employed in the pilot to maximise recruitment,[31] and invitation materials co-produced with patients. It is generally considered preferable though for a usual care provider, with whom a patient has an established relationship, to invite a person into research. Difficulties identifying the target population (see 1.0) meant we had local ED clinicians do the inviting. A future trial might consider asking EDs to identify ostensibly eligible patients, but for the GPs of the identified patients to do the inviting. This may boost recruitment.[59]

4.2.2 Effect of intervention:

Our pilot estimated SAFE's effect to be modest (reducing ED visits from 2.1 over 12-months to 1.8). This has negative consequences for a definitive trial, not least because it requires it to have a large sample to detect the effect. Efficacy was not though our pilot's primary focus and the estimate may be imprecise. Indeed, it might be that those who declined to participate in the trial and who appeared to differ in some important ways, might have benefited more. Previous evidence does though suggest it is in the region expected.

For peer review only

Specifically, only one RCT – by Sajatovic et al.[60] – has considered change in ED use following epilepsy self-management.[61] Conducted in the U.S. it compared SMART – an 8-session group intervention – to wait-list control. No significant change between groups was found in subsequent ED/ hospitalization use. For SMART, it reduced from a mean of 1.22 by .44 over the 6 months following randomisation. For the controls it reduced from 2.4 by -1.26 visits.

4.3 Judgement regarding progression to definitive trial

Thabane et al.[62] provide a framework for judging whether to progress to a definitive trial. In satisfying only one progression criteria, a definitive trial based on the pilot's design is not feasible. We have also not identified any modifications that will make it feasible. We therefore recommend not proceeding.

4.4 Strengths and limitations

Strengths include the pilot being reported according to guidelines,[63] allocations being concealed, that patients were recruited from sites similar to those likely for a definitive trial and we included a microcosting of SAFE's delivery.

The pilot is not without potential weaknesses. Most importantly, despite recruited patient participants stating sufficient ED use when screened, routine data subsequently suggested ~40% did not meet the inclusion criteria. This could limit our findings external validity and attenuate SAFE's effect. Another potential limitation is we estimated the effect of SAFE only on the proposed primary measure. We did not estimate its wider effects, including on duration of ED visits and admissions.

5. CONCLUSION

A definitive trial of SAFE is not currently feasible. Research is required to determine how people from the target population can be better recruited.

For peer review only - http://bmjopen.bmj.com/site/about/guidelines.xhtml

ACKNOWLEDGEMENTS: We thank the people who kindly participated in this study and the local ED sites for their support – namely, Arrowe Park Hospital (Wirral University Teaching Hospital NHS Foundation Trust), and Aintree University Hospital and the Royal Liverpool University Hospitals (Liverpool University Hospitals NHS Foundation Trust). Other persons who contributed to this work are: Mrs. Juliet Bransgrove [JB] (epilepsy nurse specialist), Dr Duncan Appelbe (website development/ management) and Ms. Gail Moors [GM] (Administrator). We also acknowledge the support we received from our Steering Committee (Prof. Alasdair Gray [chair], Prof. Pete Bower, Dr. Paul Cooper, Mr. Mike Jackson, Ms. Helen Coyle, Mr. Mike Perry, Mrs. Linda Perry, Mrs. Jayne Burton and Mr. Sam Burton). The study sponsor was the University of Liverpool (reference: UoL001108) and the Clinical Trials Research Centre's registration number is 12.

AUTHORS CONTRIBUTIONS: AJN and LR conceived of the study and designed it together with AGM, CTS, DH, MM and SG. SJN and CTS planned and conducted the statistical analysis, EH and DH the economic analysis. AJN was the chief investigator, with MB, JM and EM leading participant identification and DS recruitment and follow-up. AJN wrote the manuscript, with revisions and approval of the final manuscript coming from all authors.

FUNDING STATEMENT: This project was funded by the National Institute for Health Research's Health Services and Delivery Research Programme (HS&DR Programme) (project number 14/19/09). The views and opinions expressed herein are those of the authors and do not necessarily reflect those of the University of Liverpool, the HS&DR programme, the NIHR, the NHS, or the Department of Health and Social Care.

COMPETING INTERESTS: None declared.

Competing interests: None declared.

PATIENT CONSENT FOR PUBLICATION: Not required.

DATA STATEMENT: Requests for anonymised data should be submitted to the corresponding author.

For peer review only - http://bmjopen.bmj.com/site/about/guidelines.xhtml

Table 1 SAFE course and its content

Overview

SAFE was developed to be delivered to groups of ~10 patient-SO dyads by a single facilitator with epilepsy knowledge who follows a treatment manual. Materials include standardised presentation slides, videos and interactive tasks. Patients are given information packs that include copies of the slides, wallet sized first aid instructions cards, paper epilepsy ID Cards and instructions for setting up IDs on smart-phone, and access to a website with the intervention's content on.

SAFE's behaviour change potential is supported by reference to Self-Affirmation Theory.

SAFE's order of presentation and content is as follows:

Order	Topics	Learning activity	Minutes allotted
1	Welcome	Slide	5
2	Goals of this course	Slide	2
3	What would you like from today?	Interactive	20
4	True or false?	Interactive	12
5	Taking on information (Kindness Questionnaire)	Interactive	10
6	Epilepsy, seizures & how the brain works	Video	10
7	First aid for convulsive seizures	Interactive	10
8	What can you do to help someone during a seizure?	Slide	5
9	What not to do during a seizure	Slide	5
10	What to do after the seizure has stopped	Slide	5
11	Questions or comments?	Interactive	10
12	Post-seizure states	Slide	15
13	Injuries	Slide	2
14	When to call an ambulance?	Slide	10
15	Questions or comments?	Slide	10
16	Refreshment break	Networking	10
17	Recovery position	Slide	2
18	Recovery position	Video	2
19	Let's practice the recovery position	Interactive	15
20	Questions or comments?	Interactive	5
21	Who needs to know how to help?	Interactive	5
22	What they need to know & why	Slide	5

For peer review only - http://bmjopen.bmj.com/site/about/guidelines.xhtml

23	How to get this information to them. Family, friends & work colleagues:	Slide	5
24	How to get this information to them. Members of the public and health workers:	Slide	5
25	Questions or comments?	Interactive	5
26	Refreshment break	Networking	5
27	Personal stories - introduction	Slide	2
28	Ben's story (case vignette)	Slide	6
29	How to change what happened to Ben? (Carrying medical ID; triggers)	Interactive	5
30	Triggers	Slide	5
31	Knowing your triggers	Slide	4
32	Some ways of dealing with triggers	Slide	4
33	Sandra's story (case vignette)	Slide	6
34	How to change what happened to Sandra (Warning signs; home safety)	Interactive	2
35	Main points to remember, If you have epilepsy:	Slide	3
36	Main points to remember; If you know someone with epilepsy:	Slide	2
37	Sources of further information	Slide	2
38	What's on the back table and accessing the study website*	Slide	2
39	Questions or comments?	Slide	2
40	Evaluation		-
41	Certificates of attendance		-
**Time in minutes:** 240			

Notes:

* http://www.seizurefirstaid.org.uk/Intervention/

For peer review only - http://bmjopen.bmj.com/site/about/guidelines.xhtml

Table 2 Proposed primary and secondary outcome measures for a definitive trial used within pilot by assessment and participant type [a]

Outcome	Domain	Participants	Measure and derivation of outcome	Baseline (T0)	3-month (T1)	6-month (T2)	12 month (T3)
Primary	ED visits	PWE	*Routine data, Hospital Episode Statistics Accident & Emergency system*<br>NHS numbers of patient participants securely transferred to NHS Digital who hold the Hospital Episode Statistics system. NHS Digital then provided the number of ED visits these patients were recorded as having made in the 12 months prior to and in the 12 months following their randomisation. [b]	✓	-	-	✓
Secondary	ED visits	PWE	*Self-report, "How many times have you used Casualty / Accident & Emergency (A&E) over the past 12 months for epilepsy?"*<br>(For T2 the time frame was 6 months)<br>Provided with seizure diary at T0 to assist.	✓	-	✓	✓
	Seizure control	PWE	Baseline: *Thapar's*[64] *Seizure Frequency Scale* for the prior 12 months.<br>T2 and T3: Asked for number of epileptic seizures (of any type) since last assessment and dates of the first and most recent. Provided with seizure diary at T0 to assist.	✓	-	✓	✓
	Quality of life	PWE	*Quality of Life in Epilepsy Scale-31-P (31 items)* [65]	✓	-	✓	✓
	Distress	PWE; SOs	*Hospital Anxiety and Depression Scale (14 items)* [66] [c]	✓	-	-	✓
	Felt Stigma	PWE; SOs	*Stigma of Epilepsy Scale (9 items)* [67] [d]	✓	-	-	✓
	Burden	SOs	*Zarit Caregiver Burden Inventory (22 items)* [49] [e]	✓	-	✓	✓
	Adverse events	PWE	*Monitored by PWE completing a checklist.* [f]	-	✓	✓	✓
	Fear of seizures	PWE; SOs	*Epilepsy Knowledge and Management Questionnaire – Fears subscale (5 items)* [68]	✓	-	-	✓

For peer review only - http://bmjopen.bmj.com/site/about/guidelines.xhtml

	Knowledge of what to do when faced with a seizure	PWE; SOs	*Thinking About Epilepsy Questionnaire (13 items)*[69]	✓	-	-	✓
	Confidence managing seizures/ epilepsy	PWE; SOs	PWE: *Epilepsy Mastery Scale (6 items)* [70]<br>SOs: *Parents Response to Child Illness Scale- Condition Management subscale* (6 items) [71]	✓	-	✓	✓
	Health economics	PWE	*Client Service Receipt Inventory* [72] and *EQ-5D* (13 items) [73]	✓	-	-	✓
-	Feedback on trial participation	PWE; SOs	Adapted from Magpie Trial (3 items) [74]<br>(1) "If time suddenly went backward, and you had to do it all over again, would you agree to participate in the Seizure First Aid Training trial?";<br>(2) "Please tell us if there was anything about the Seizure First Aid Training Trial that you think could have been done better";<br>(3) "Please tell us if there was anything about the Seizure First Aid Training Trial, or your experience of joining the trial, that you think was particularly good"	-	-	-	✓

Notes:

ED= Emergency Department; PWE= Person with epilepsy; SOs= Significant others.

[a] Unless a patient formally withdrew consent to participate in the trial, routine data on their ED use was requested and up to 3 attempts were made to contact patients or SO participant each time one of their follow-up assessments was due.

[b] To minimise cost, a single application for routine data was submitted, rather than one relating to the baseline period and one for follow-up.

[c] Total anxiety and total depression scores were categorised according to the following: 0 to 7, 'Normal range'; 8 to 10, 'Suggestive of anxiety / depression'; 11 to 21, 'Probable anxiety / depression.'

[d] Total stigma scores were categorised as follows: 0, 'No stigma'; 1 to 6, 'Mildly to moderately stigmatized'; 7 to 9, 'Highly stigmatized;

[e] Total Zarit Burden scores were categorised as follows: 0 to 20, 'Little or no burden', 21 to 40, 'Mild to moderate burden', 41 to 60, 'Moderate to severe burden'; 61 to 88, 'Severe burden.'

[f] Definitions of serious adverse event and how relatedness was assessed is presented in Supplementary File 9.

For peer review only

Table 3 Participant inclusion and exclusion criteria

Participants	Inclusion criteria	Exclusion criteria
Patients	• Established diagnosis of epilepsy (≥1 year);<br>• All epilepsy syndromes and all types of focal and generalised seizures;<br>• Currently being prescribed antiepileptic medication;<br>• Age ≥16 years (no upper age limit);<br>• Visited an ED for epilepsy on ≥2 occasions within the previous 12 months (as reported by patient);<br>• Live within 25 miles of any of the ED recruitment sites;<br>• Able to provide informed consent, participate in intervention and independently complete questionnaires in English.	• Actual or suspected psychogenic non-epileptic seizures alone or in combination with epilepsy;<br>• Acute symptomatic seizures related to acute neurological illness or substance misuse;<br>• Severe current psychiatric disorders (e.g. acute psychosis) or life-threatening medical illness;<br>• Enrolled in other epilepsy related non-pharmacological treatment studies.
Carers	• A significant other to the patient (e.g., family member, friend) who the patient identifies as providing informal support;<br>• Age ≥16 years (no upper age limit);<br>• Lives in the North West area of England	• Severe current psychiatric disorders or life threatening medical illness;<br>• Enrolled in other epilepsy related non-pharmacological treatment studies.

Notes:

Patient participants were permitted to take part without a carer. Carers could not, however, take part without a patient partner having at least consented to take part into the study.

For peer review only - http://bmjopen.bmj.com/site/about/guidelines.xhtml

Table 4 Characteristics of eligible patients who did and did not agree to participate

Variable		Agreed to participate *SAFE + TAU* (n=26)	Agreed to participate *TAU* (n=25)	Agreed to participate *Combined* (n=51)	Did not agree to participate (n=379)[b]
Sex: n (%)					
Male		10 (38.5%)	12 (48.0%)	22 (43.1%)	192 (50.7%)
Female		16 (61.5%)	13 (52.0%)	29 (56.9%)	187 (49.3%)
Age at presentation to emergency department (years)					
Mean		39.2	40.7	39.9	40.6
Standard deviation		13.96	17.52	15.66	16.83
Median (min., max.)		37.1 (18.9, 69.9)	41.4 (16.5, 71.3)	38.8 (16.4, 71.3)	37.5 (16.2, 84.4)
Index of Multiple Deprivation (IMD)[a]					
IMD Decile: 1	n (%)	11 (42.3%)	14 (56.0%)	25 (49.0%)	188 (49.6%)
IMD Rank	Median (min., max.)	574.0 (44, 3202)	1231.5 (48, 2166)	673.0 (44, 3202)	924.5 (28, 3107)
IMD Decile: 2 to 3	n (%)	7 (26.9%)	6 (24.0)	13 (25.4%)	82 (21.6%)
IMD Rank	Median (min., max.)	6785 (4649, 8281)	6665 (3989, 7816)	6785 (3989, 8281)	5309 (3291,9835)
IMD Decile: 4 to 6	n (%)	2 (7.7%)	4 (16.0%)	6 (11.8%)	59 (15.6%)
IMD Rank	Median (min., max.)	12836 (9881, 15791)	11924 (11480, 16004)	11924 (9881, 16004)	14459 (10277, 19659)
IMD Decile: 7 to 10	n (%)	5 (19.2%)	1 (4.0%)	6 (11.8%)	50 (13.2%)
IMD Rank	Median (min., max.)	27642 (24971, 31002)	32724 (32724, 32724)	28876 (24971, 32724)	22673 (19826, 32396)
	Missing	1 (3.8%)	0 (0.0%)	1 (2.0%)	0 (0.0%)
Ethnicity: n (%)					
White		25 (96.2%)	23 (92.0%)	48 (94.1%)	-
Asian / Asian British		0 (0.0%)	0 (0.0%)	0 (0.0%)	-
Black / African / Caribbean / Black British		1 (3.8%)	1 (4.0%)	2 (3.9%)	-
Mixed / multiple ethnic groups		0 (0.0%)	0 (0.0%)	0 (0.0%)	-
Other ethnic group		0 (0.0%)	1 (4.0%)	1 (2.0%)	-
Other significant medical history: n (%)					
No, none		13 (50.0%)	10 (40.0%)	23 (45.1%)	-
Yes, another medical condition/s		10 (38.5%)	13 (52.0%)	23 (45.1%)	-

For peer review only - http://bmjopen.bmj.com/site/about/guidelines.xhtml

Yes, a psychiatric condition	1 (3.8%)	0 (0.0%)	1 (2.0%)	-
Yes, both medical and psychiatric conditions	2 (7.7%)	2 (8.0%)	4 (7.8%)	-
Education: n (%)				
O' levels/ GCSEs/ Level 1 or 2 NVQ	13 (50.0%)	14 (56.0%)	27 (53.0%)	-
A' Levels/ Level 3 NVQ	5 (19.2%)	3 (12.0%)	8 (15.7%)	-
University degree/ Graduate Certificate or Diploma	5 (19.2%)	5 (20.0%)	10 (19.6%)	-
Postgraduate university degree (e.g., PGCE, MSc, MA, PhD)	2 (7.7%)	0 (0.0%)	2 (3.9%)	-
Missing	1 (3.9%)	3 (12.0%)	4 (7.8%)	-
Time since epilepsy diagnosis (years)				
Mean	19.9	22.6	21.2	-
Standard deviation	14.85	18.38	16.57	-
Median (mix., max.)	16.8 (1.8, 53.9)	19.3 (1.7, 64.9)	17.3 (1.7, 64.9)	-
Time since last epileptic seizure (days)				
Mean	53.6	40.1	47.0	-
Standard deviation	101.10	61.21	83.34	-
Median (min., max.)	14.0 (1, 340)	10.5 (0, 235)	14.0 (0, 340)	-
Missing	3 (11.5%)	3 (12.0%)	6 (11.8%)	-
Number of epileptic seizures in the last 12 months				
0-3	4 (15.3%)	3 (12.0%)	7 (13.8%)	-
4-6	1 (3.8%)	5 (20.0%)	6 (11.7%)	-
7-9	3 (11.5%)	3 (12.0%)	6 (11.7%)	-
10 or more	18 (69.2%)	14 (56.0%)	32 (62.8%)	-

Notes:

[a] The Index of Multiple Deprivation (IMD) ranks every small area in England from 1 (most deprived area) to 32,844 (least deprived area).IMD rank and decile missing for one recruited participant as they resided in Wales.; [b] Includes seven patients who could not be contacted due to an incorrect postal address and two patients who were not sent an invite letter in error.min.= minimum, max.= maximum.

For peer review only - http://bmjopen.bmj.com/site/about/guidelines.xhtml

Table 5 Number of ED visits patient participants made according to routine data

Number of ED visits		SAFE+TAU (n=26)	TAU (n=25)	Total (n=51)
In the 12 months to baseline	Mean	2.1	3	2.5
	Standard deviation	2.22	2.76	2.51
	Median (min., max.)	1 (0, 10)	2 (1, 12)	2 (0, 12)
	Missing	1 (3.8%)	2 (8.0%)	3 (5.9%)
In the 12 months following randomisation	Mean	1.8	3.4	2.6
	Standard deviation	3.14	4.78	4.05
	Median (min., max.)	1 (0, 12)	2 (0, 20)	1 (0, 20)
	Missing	1 (3.8%)	2 (8.0%)	3 (5.9%)
Change from baseline over 12 months following randomisation	Mean	-0.3	0.4	0.1
	Standard deviation	1.99	3.81	2.99
	Median (min., max.)	0 (-4, 5)	0 (-5, 16)	0 (-5, 16)
	Missing	1 (3.8%)	2 (8.0%)	3 (5.9%)

For peer review only

Table 6 Between intervention group differences in number of ED visits

Model and parameter	Parameter	Confidence Interval (CI)			P-value
		95%	90%	80%	
12 months following randomisation according to routine data[a]					
Negative binomial: SAFE + TAU (Rate Ratio)	0.54	0.24 to 1.18	0.28 to 1.04	0.32 to 0.90	0.12
Negative binomial: Dispersion parameter	1.53	0.67 to 2.39	0.80 to 2.25	0.96 to 2.09	NA
Vuong's Test[b]	-0.17	NA	NA	NA	0.87
12 months following randomisation according to routine data with adjustment for baseline ED visits					
Negative binomial: SAFE + TAU (Rate Ratio)	0.62	0.33 to 1.17	0.36 to 1.06	0.41 to 0.94	0.14
Negative binomial: Baseline ED visits (Rate Ratio)	1.33	1.18 to 1.52	1.20 to 1.49	1.23 to 1.45	<0.001
Negative binomial: Dispersion parameter	0.69	0.17 to 1.21	0.26 to 1.13	0.35 to 1.03	NA
Vuong's Test[b]	-0.13	NA	NA	NA	0.90

Notes:

ED=Emergency Department; SAFE=Seizure First Aid Training; TAU=Treatment-As-Usual,

[a] Analysis based on 48 patient participants

[b] Vuong's test p-value interpretation, a significantly negative parameter value favours the Negative Binomial model and significantly positive favours the Zero-inflated Negative Binomial model. A non-significant value indicates no significant difference between the models therefore the simpler Negative Binomial model is preferred.[75]

For peer review only

For peer review only - http://bmjopen.bmj.com/site/about/guidelines.xhtml

Table 7 Required sample size for a definitive trial to detect estimated effect of SAFE+TAU on ED use

**Dispersion parameter (*k*)**	**~47% reduction (from 3.4 to 1.8 visits) in 12 months compared to TAU**	
	**n per group** (α= 0.05; 80% power, β=0.2)	**n per group** (α= 0.05; 90% power, β=0.1)
0.17	123	164
0.5	191	255
0.69	230	308
1	293	393
1.21	337	451

Notes:
Dispersion parameter taken from the adjusted NBR model in the pilot RCT (i.e., k=0.69; 95% CI: 0.17 to 1.21); *see* Table 6

For peer review only

Figure Legend

Figure 1 CONSORT diagram of flow through the pilot trial

Figure 2 a) Bland Altman plot of agreement between self-reported ED use and routine data on ED use in 12 months prior to randomisation; b) Bland Altman plot of agreement between self-reported ED visits and routine data on ED use over 12 months following randomisation.

For peer review only

For peer review only - http://bmjopen.bmj.com/site/about/guidelines.xhtml

References

1. Dixon PA, Kirkham JJ, Marson AG, et al. National Audit of Seizure management in Hospitals (NASH): results of the national audit of adult epilepsy in the UK. *BMJ Open* 2015;5:e007325.
2. Rizos T, Jüttler E, Sykora M, et al. Common disorders in the neurological emergency room – experience at a tertiary care hospital. *European Journal of Neurology* 2011;18:430–35.
3. Royl G, Ploner CJ, Möckel M, et al. Neurological chief complaints in an emergency roo. *Der Nervenarzt* 2010;81(10):1226-30.
4. Marson T, Taylor C, Tudur-Smith C, et al. Seizure management in hospitals in Europe. ILAE 13th European Congress on Epileptology. Vienna, 2018.
5. Hart YM, Shorvon SD. The nature of epilepsy in the general population. II. Medical care. *Epilepsy research* 1995;21(1):51-58.
6. Jacoby A, Buck D, Baker G, et al. Uptake and costs of care for epilepsy: findings from a UK regional study. *Epilepsia* 1998;39(7):776-86.
7. Kitson A, Shorvon S. Services for patients with epilepsy: Report of a CSAG committee: Department of Health, 2000.
8. Dickson JM, Jacques R, Reuber M, et al. Emergency hospital care for adults with suspected seizures in the NHS in England 2007–2013: a cross-sectional study. *BMJ open* 2018;8(10):e023352.
9. Pearson M, Marson A, Dixon P, et al. National Audit of Seizure Management in Hospitals: St. Elsewhere's Hospital Clinical Report 2014 [Available from: http://www.nashstudy.org.uk/Newsletters/St%20Elsewhere's%20Clinical%20Report%20NASH%202.pdf accessed 9th April 2019.
10. Whiston S, Coyle B, Chappel D. Health needs assessment for long term neurological conditions in North East England. Stockton on Tees: North East Public Health Observatory 2009.
11. Noble AJ, Goldstein LH, Seed P, et al. Characteristics of people with epilepsy who attend emergency departments: Prospective study of metropolitan hospital attendees. *Epilepsia* 2012;53(10):1820–28.
12. NHS England. NHS Outcomes Framework indicators - Feb 2017 release. 2017 [Available from: https://digital.nhs.uk/data-and-information/publications/clinical-indicators/nhs-outcomes-framework/archive/nhs-outcomes-framework-indicators---february-2017-release accessed 8th August 2017.
13. House of Commons Committee of Public Accounts. Services to people with neurological conditions: progress review London House of Commons; 2015 [Available from: https://publications.parliament.uk/pa/cm201516/cmselect/cmpubacc/502/502.pdf accessed 4th April 2019.
14. Ridsdale L, McCrone P, Morgan M, et al. Can an epilepsy nurse specialist-led self-management intervention reduce attendance at emergency departments and promote well-being for people with severe epilepsy? A non-randomised trial with a nested qualitative phase. *Health Services and Delivery Research* 2013;1(9)
15. Dickson JM, Taylor LH, Shewan J, et al. Cross-sectional study of the prehospital management of adult patients with a suspected seizure (EPIC1). *BMJ Open* 2016;6:e010573.

For peer review only - http://bmjopen.bmj.com/site/about/guidelines.xhtml

16. Peter A Dixon, Jamie J Kirkham, Anthony G Marson, et al. National Audit of Seizure management in Hospitals (NASH): results of the national audit of adult epilepsy in the UK *BMJ Open* 2015;5(3):e007325.
17. Reuber M, Hattingh L, Goulding PJ. Epileptological emergencies in Accident and Emergency: a survey at St James's University Hospital, Leeds. *Seizure* 2000;9(3):216-20.
18. NHS Choices. What to do if someone has a seizure (fit) 2017 [Available from: https://www.nhs.uk/conditions/what-to-do-if-someone-has-a-seizure-fit/ accessed 4th April 2019.
19. Epilepsy Action. What to do when someone has a seizure 2016 [Available from: https://www.epilepsy.org.uk/info/firstaid/what-to-do accessed 4th April 2019.
20. Ashworth M, Seed P, Armstrong D, et al. The relationship between social deprivation and the quality of primary care: a national survey using indicators from the UK Quality and Outcomes Framework. *Br J Gen Pract* 2007;57(539):441-48.
21. Bautista RED, Glen ET, Wludyka PS, et al. Factors associated with utilization of healthcare resources among epilepsy patients. *Epilepsy research* 2008;79(2-3):120-29.
22. QIPP/Right Care. The NHS atlas of variation in healthcare 2010 [Available from: fingertips.phe.org.uk/documents/Atlas_2010%20Compendium.pdf.
23. Allard J, Shankar R, Henley W, et al. Frequency and factors associated with emergency department attendance for people with epilepsy in a rural UK population. *Epilepsy & Behavior* 2017;68:192-95.
24. Faught E, Duh MS, Weiner JR, et al. Nonadherence to antiepileptic drugs and increased mortality: findings from the RANSOM Study. *Neurology* 2008;71(20):1572-78.
25. Ridsdale L, Charlton J, Ashworth M, et al. Epilepsy mortality and risk factors for death in epilepsy: a population-based study. *Br J Gen Pract* 2011;61(586):e271-e78.
26. Health and Social Care Information Centre. Hospital Accident and Emergency Activity Supporting Information, 2017-18 2018 [cited Available from: https://files.digital.nhs.uk/8B/94BB9E/AE1718_supporting_information.pdf accessed 27th August 2019.
27. International League Against Epilepsy - British Branch. Epilepsy health services for epilepsy - the proceedings of an expert workshop London2016 [Available from: https://ilaebritish.org.uk/epilepsy-emergency-care/ accessed 22nd October 2019.
28. National Clinical Guideline Centre. Pharmacological Update of Clinical Guideline 20. The Epilepsies: The Diagnosis and Management of the Epilepsies in Adults and Children in Primary and Secondary Care London: National Clinical Guideline Centre (UK); 2012 [Available from: ncbi.nlm.nih.gov/pubmedhealth/PMH0068980.
29. Noble AJ, Morgan M, Virdi C, et al. A nurse-led self-management intervention for people who attend emergency departments with epilepsy: the patients' view. *Journal of Neurology* 2013;260(4):1022–30.
30. Ridsdale L, Virdi C, Noble AJ, et al. Explanations given by people with epilepsy for using emergency medical services: a qualitative study. *Epilepsy Behaviour* 2012;25:529–33.
31. Noble AJ, Marson AG, Tudur-Smith C, et al. 'Seizure First Aid Training' for people with epilepsy who attend emergency departments, and their family and

For peer review only - http://bmjopen.bmj.com/site/about/guidelines.xhtml

friends: study protocol for intervention development and a pilot randomised controlled trial. *BMJ Open* 2015;5:e009040.
32. Snape DA, Morgan M, Ridsdale L, et al. Developing and assessing the acceptability of an epilepsy first aid training intervention for patients who visit UK emergency departments: A multi-method study of patients and professionals. *Epilepsy and Behavior* 2017;68:177-85.
33. Craig P, Dieppe P, Macintyre S, et al. Developing and evaluating complex interventions: the new Medical Research Council guidance. *Bmj* 2008;337:a1655.
34. Lancaster GA, Dodd S, Williamson PR. Design and analysis of pilot studies: recommendations for good practice. *Journal of Evaluation in Clinical Practice* 2004 10(2):307-12.
35. Department of Health. Health Profile 2012. Liverpool. 2012 [Available from: http://fingertipsreports.phe.org.uk/health-profiles/2012/e08000012.pdf accessed 11th April 2018.
36. Department of Health. Health Profile 2012. Wirral 2012 [Available from: http://fingertipsreports.phe.org.uk/health-profiles/2012/e08000015.pdf accessed 11th April 2018.
37. Noble AJ, Snape D, Ridsdale L, et al. Assessing treatment fidelity within an epilepsy randomized controlled trial: Seizure first aid training for people with epilepsy who visit emergency departments. *Behavioural Neurology* 2019;19:1-11.
38. Sim J, Lewis M. 2012. *Journal of Clinical Epidemiology* The size of a pilot study for a clinical trial should be calculated in relation to considerations of precision and efficiency;65:301-08.
39. Digital N. Accident and Emergency Attendances in England - 2012-13 2014 [Available from: https://digital.nhs.uk/data-and-information/publications/statistical/hospital-accident--emergency-activity/accident-and-emergency-attendances-in-england-2012-13 accessed 22nd October 2019.
40. Bhatt H, Matharu MS, Henderson K, et al. An audit of first seizures presenting to an Accident and Emergency Department. *Seizure* 2005;14:58-61.
41. Julious SA. Sample size of 12 per group rule of thumb for a pilot study. *Pharmaceutical Statistics* 2005;4:287–91.
42. Lee EC, Whitehead AL, Jacques RM, et al. The statistical interpretation of pilot trials: should significance thresholds be reconsidered? *BMC Medical Research Methodology* 2014;14:41.
43. Bland JM, Altman D. Statistical methods for assessing agreement between two methods of clinical measurement. *The Lancet* 1986;327:307-10.
44. Noble AJ, Marson AG. Which outcomes should we measure in adult epilepsy trials? The views of people with epilepsy and informal carers. *Epilepsy and Behavior* 2016;59:105-10.
45. Keene ON, Jones MR, Lane PW, et al. Analysis of exacerbation rates in asthma and chronic obstructive pulmonary disease: example from the TRISTAN study. *Pharmaceutical Statistics* 2007;6:89-97.
46. Sach TH, Dsborough J, Houghton J, et al. Applying microcosting methods to estimate the costs of pharmacy interventions: an illustration using multiprofessional clinical medication reviews in care homes for older people. *International Journal of Pharmacy Practice* 2015;23:237-47.

For peer review only - http://bmjopen.bmj.com/site/about/guidelines.xhtml

47. Lopetegui M, Yen PY, Lai A, et al. Time motion studies in healthcare: what are we talking about? *Journal of Biomedical Informatics* 2014;49: 292-99.
48. Piccenna L, Walker W, Shears G, et al. Priorities for epilepsy research: A briefing report 2017 [Available from: https://pdfs.semanticscholar.org/5861/867345dc168e35918e386e7beaa89c7c6586.pdf accessed 23rd October 2019.
49. Zarit SH, Reever KE, Bach-Peterson J. Relatives of the impaired elderly: correlates of feelings of burden. *Gerontologist* 1980;20:649-55.
50. Crawford JR, Henry JD, Crombie C, et al. Normative data for the HADS from a large non-clinical sample. *British Journal of Clin Psychology* 2001;40(4):429-34.
51. Walters SJ, Bonacho Dos Anjos Henriques-Cadby I, Bortolami O, et al. Recruitment and retention of participants in randomised controlled trials: a review of trials funded and published by the United Kingdom Health Technology Assessment Programme. *BMJ Open* 2017;7:e015276.
52. Monfared AA, Lelorier J. Accuracy and validity of using medical claims data to identify episodes of hospitalizations in patients with COPD. *Pharmacoepidemiology and drug safety* 2006;15:19-29.
53. Roberts RO, Bergstralh EJ, Schmidt L, et al. Comparison of self-reported and medical record health care utilization measures. *Journal of Clinical Epidemiology* 1996;49:989-95.
54. Medical Research Council. Obtaining data from NHS Digital for health research – a guide for researchers 2016 [Available from: https://mrc.ukri.org/documents/pdf/obtaining-data-from-nhs-digital-v101016/ accessed 10th October 2016.
55. Raftery J, Young A, Stanton L, et al. Clinical trial metadata: defining and extracting metadata on the design, conduct, results and costs of 125 randomised clinical trials funded by the National Institute for Health Research Health Technology Assessment programme. *Health Technology Assessment* 2015;19:1-138.
56. National Institute for Health Research. HTA Supporting Information: Reasonable costs and value for money 2019 [Available from: https://www.nihr.ac.uk/documents/hta-supporting-information/11929#Reasonable_costs_and_value_for_money accessed 22nd October 2019.
57. Ridsdale L, Wojewodka G, Robinson EJ, et al. The effectiveness of a group self-management education course for adults with poorly controlled epilepsy, SMILE (UK): A randomized controlled trial. *Epilepsia* 2018 59(5):1048-61.
58. Ridsdale L, Wojewodka G, Robinson E, et al. Characteristics associated with quality of life among people with drug-resistant epilepsy. *Journal of Neurology* 2017;246(6):1174-84.
59. Hewitson P, Ward AM, Heneghan C, et al. Primary care endorsement letter and a patient leaflet to improve participation in colorectal cancer screening: results of a factorial randomised trial. *British Journal of Cancer* 2011;105(4):475-80.
60. Sajatovic M, Colon-Zimmermann K, Kahriman M, et al. A 6-month prospective randomized controlled trial of remotely delivered group format epilepsy self-management versus waitlist control for high-risk people with epilepsy. *Epilepsia* 2018 59(9):1684-95.
61. Luedke MW, Blalock DV, Goldstein KM, et al. Self-management of epilepsy: A systematic review. *Annals of Internal Medicine* 2019 171(2):117-26.

For peer review only - http://bmjopen.bmj.com/site/about/guidelines.xhtml

62. Thabane L, Ma J, Chu R, et al. A tutorial on pilot studies: the what, why and how. *BMC Medical Research Methodology* 2010;10(1)
63. Eldridge SM, Chan CL, Campbell MJ, et al. CONSORT 2010 statement: extension to randomised pilot and feasibility trials. *British Medical Journal* 2016;355:i5239.
64. Thapar A, Kerr M, Harold G. Stress, anxiety, depression, and epilepsy: Investigating the relationship between psychological factors and seizures. *Epilepsy and Behavior* 2009;14:134-40.
65. Cramer J, Perrine K, Devinsky O, et al. Development and cross-cultural translations of a 31-item quality of life in epilepsy inventory. *Epilepsia* 1998;39:81-88.
66. Zigmond AS, Snaith RP. The hospital anxiety and depression scale. *Acta psychiatrica Scandinavica* 1983;67:361-70.
67. Taylor J, Baker GA, Jacoby A. Levels of epilepsy stigma in an incident population and associated factors. *Epilepsy and Behavior* 2011;21(255-260)
68. Mittan R. Fear of seizures. In: Whitman S, Hermann B, eds. Psychopathology in epilepsy: social dimensions New York, NY: Oxford University Press 1986:90–121.
69. Martiniuk ALC, Campbell MK. Development and psychometric properties of the Thinking about Epilepsy questionnaire assessing children's knowledge and attitudes about epilepsy. *Epilepsy and Behavior* 2007;10:595-603.
70. Wagner AK, Keller SD, Kosinski M, et al. Advances in methods for assessing the impact of epilepsy and antiepileptic drug therapy on patients' health-related quality of life. *Qual of Life Research* 1995;4:115-34.
71. Shore CP, Austin JK, Dunn DW. Maternal adaptation to a child's epilepsy. *Epilepsy and Behavior* 2004;5:557-68.
72. Beecham J, Knapp M. Costing psychiatric interventions. In: Thornicroft G, Brewin C, Wing J, eds. Measuring Mental Health Needs. London: Gaskell 1992: 163-83.
73. Williams A. The role of the EUROQOL instrument in QALY calculations. York: Centre for Health Economics, University of York, 1995.
74. Smyth RM, Duley L, Jacoby A, et al. Women's experiences of participating in the Magpie Trial: a postal survey in the United Kingdom. *Birth* 2009;36:220-29.
75. Vuong Q. Likelihood ratio tests for model selection and non-nested hypotheses. *Econometrica* 1989;B:307- 33.

For peer review only

For peer review only - http://bmjopen.bmj.com/site/about/guidelines.xhtml

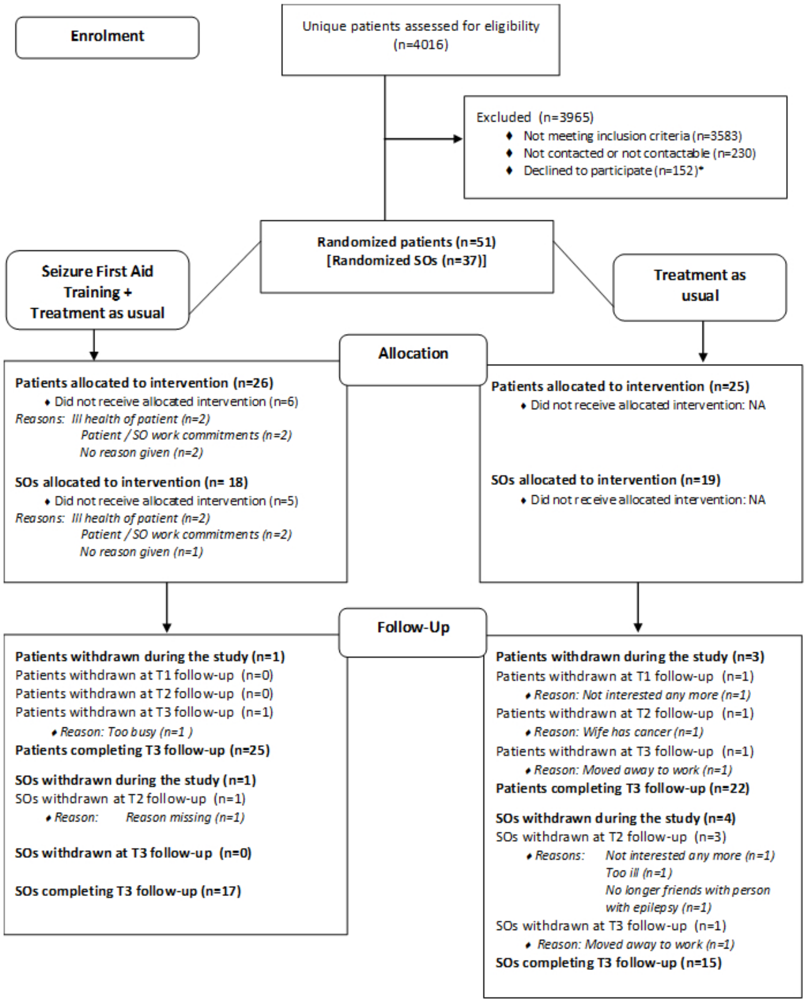

Figure 1 CONSORT diagram of flow through the pilot trial

60x88mm (300 x 300 DPI)

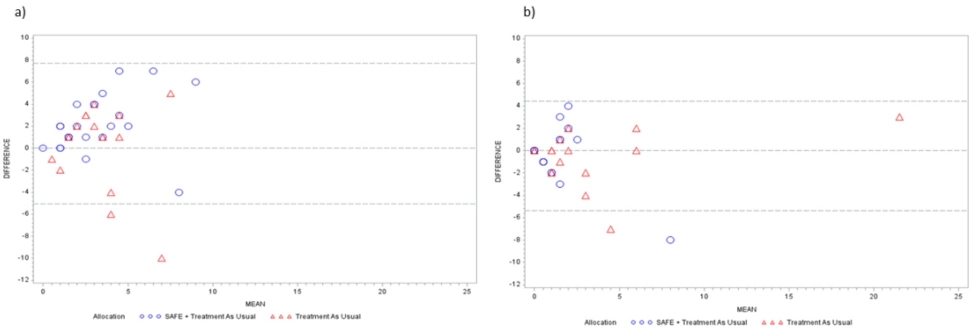

Figure 2 a) Bland Altman plot of agreement between self-reported ED use and routine data on ED use in 12 months prior to randomisation; b) Bland Altman plot of agreement between self-reported ED visits and routine data on ED use over 12 months following randomisation.

108x60mm (300 x 300 DPI)

SUPPLEMENTARY FILES

Supplementary File 1 Search criteria employed at the different recruitment sites to examine their electronic unique attendance record systems to identify potentially suitable persons attending ED for epilepsy

Recruitment site		
**Site**<br>**Aintree University Hospital**	**Site**<br>**Royal Liverpool University Hospital**	**Site**<br>**Arrowe Park Hospital**
Identify those persons who within the free text box 'presenting complaint' are recorded as having visited the ED for a:<br>• "fit"<br>• "epilepsy"<br>• "convulsion"<br>• "seizure"<br><br>And those patients admitted as an emergency from ED to a ward with any of the following primary or secondary ICD-10 diagnoses:<br>• "G40 Epilepsy"<br>• "G41 Status epilepticus"<br>• "G83.8 Other specified paralytic syndromes"<br>• "R56.8 Other and unspecified convulsions"	Identify those persons who attended the ED and were given any of the following presenting complaint-discharge diagnosis profiles:<br>• Presenting complaint "AE20 AEPRC Fit" AND ED discharge diagnosis "02 AEDIG Contusion/ abrasion";<br>• "AE20 AEPRC Fit" AND "04 AEDIG Head injury";<br>• "AE20 AEPRC Fit" AND "05 AEDIG Dislocation";<br>• "AE20 AEPRC Fit" AND "06 AEDIG Sprain/ ligament injury";<br>• "AE20 AEPRC Fit" AND "07 AEDIG Muscle/ tendon injury";<br>• "AE20 AEPRC Fit" AND "10 AEDIG Burns and scalds";<br>• "AE20 AEPRC Fit" AND "15 AEDIG Near Drowning";<br>• "AE20 AEPRC Fit" AND "33 AEDIG Facio-maxillary conditions";<br>• "AE20 AEPRC Fit" AND "38 AEDIG Diagnosis not classifiable".<br>• "241 AEDIG Epilepsy".<br><br>And those patients admitted as an emergency from ED to a ward with any of the following primary or secondary ICD-10 diagnoses:<br>• "G40 Epilepsy"<br>• "G41 Status epilepticus"<br>• "G83.8 Other specified paralytic syndromes"<br>• "R56.8 Other and unspecified convulsions"	Identify those persons who within the free text box 'reason for visit' or 'presenting complaint' are recorded as having visited the ED for:<br>• "fit"<br>• "epilep"<br>• "convuls"<br>• "seiz"<br><br>And those patients admitted as an emergency from ED to a ward with any of the following primary or secondary ICD-10 diagnoses:<br>• "G40 Epilepsy"<br>• "G41 Status epilepticus"<br>• "G83.8 Other specified paralytic syndromes"<br>• "R56.8 Other and unspecified convulsions"

Notes:

As the architectures of the systems and the coding processes at each sites varied the research team worked with local ED teams to identify the search criteria.

For peer review only

Supplementary File 2 CONSORT diagram of eligibility screening for the trial

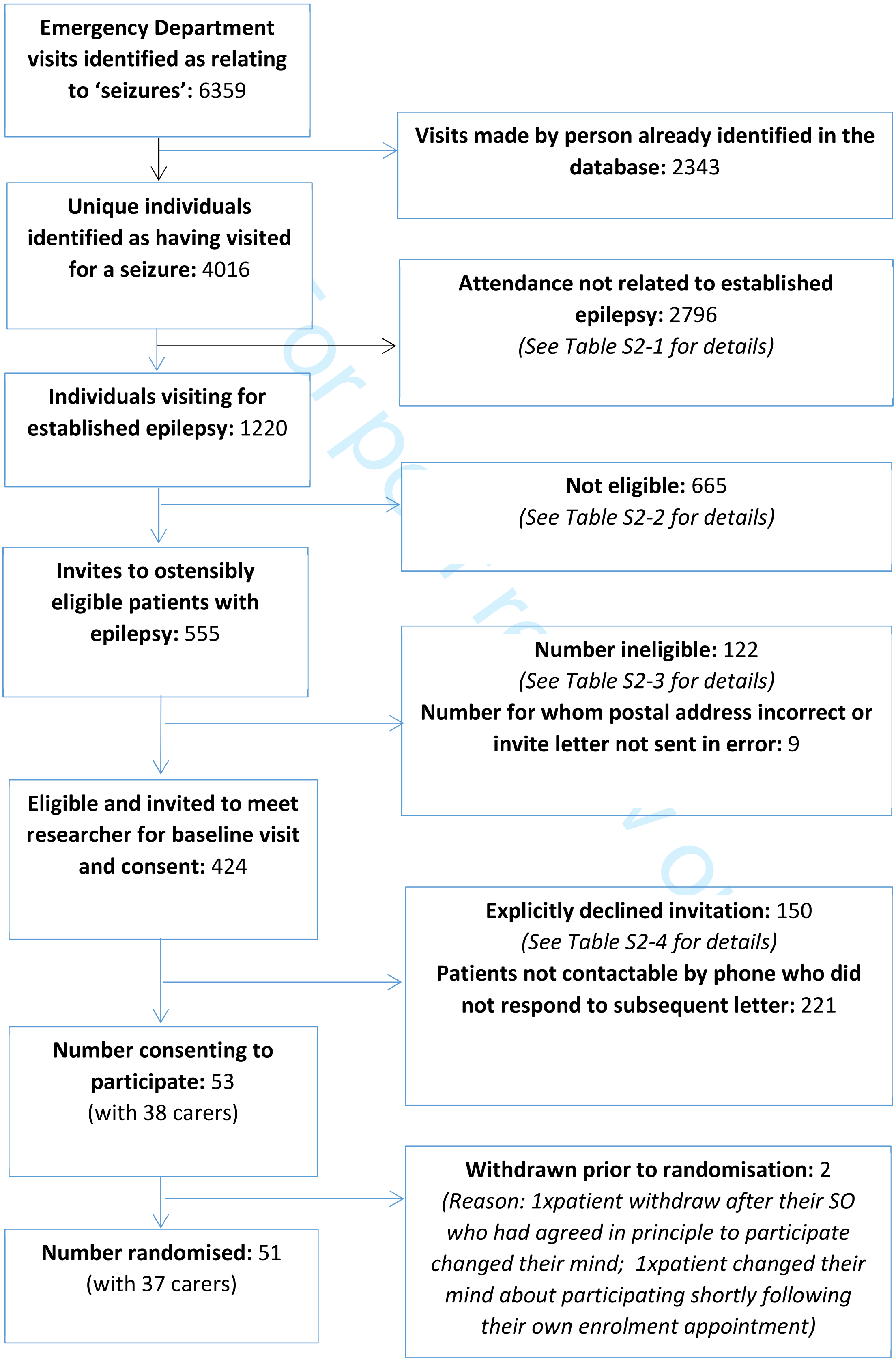

Table S2-1 Attendance at emergency department not related to epilepsy

Reason	Number of participants			
	Site 1	Site 2	Site 3	Total
No confirmed diagnosis of epilepsy/not attending because of epilepsy	737	483	715	1935
Acute symptomatic seizures related to neurological illness or substance abuse	238	240	191	669
Psychogenic non-epileptic seizures	19	55	29	103
Medical record missing	54	5	27	86
Ineligible, but no reason given	2	1	0	3
**Total**	**1050**	**784**	**962**	**2796**

Table S2-2 Other reasons for non-eligibility (individuals visiting for established epilepsy)

Reason	Number of participants			
	Site 1	Site 2	Site 3	Total
Postcode outside catchment area	148	94	60	302
Learning disability likely to impede individuals' capacity to provided signed, informed consent	45	51	52	148
Inability to converse in English and provide signed informed consent	10	24	46	80
Life threatening medical illness	35	26	10	71
Severe psychiatric disorder	11	3	18	32
No fixed abode	10	6	13	29
Participating in another trial	3	0	0	3
**Total**	**262**	**204**	**199**	**665**

Table S2-3 Reasons for non-eligibility (ostensibly eligible individuals)

Reason	Number of participants			
	Site 1	Site 2	Site 3	Total
Has not visited an ED for epilepsy on ≥2 occasions within previous 12 months (as reported by patient)	9	15	24	48
Not able to provide informed consent, participate in SAFE course if randomised or to independently complete questionnaires in English	9	7	7	23
Ineligible, but no reason given[a]	4	7	4	15
Not established diagnosis of epilepsy (<1 year)	3	2	5	10
Moved out of area; postcode no longer within 25 mile catchment area	4	1	3	8
Actual or suspected psychogenic non-epileptic seizures alone or in combination with epilepsy	2	1	4	7
Severe current psychiatric disorders or life-threatening medical illness	1	5	1	7

For peer review only - http://bmjopen.bmj.com/site/about/guidelines.xhtml

Not currently being prescribed AED/s	1	0	1	2
Acute symptomatic seizures related to acute neurological illness or substance misuse	0	1	0	1
Participating in another trial	1	0	0	1
**Total**	**34**	**39**	**49**	**122**

Notes:

a. Lack of reason due to patients returning participation slips and no stating actual reason for ineligibility

Table S2-4 Reasons for declining participation (eligible individuals)

**Reason**	**Number of participants**			
	**Site 1**	**Site 2**	**Site 3**	**Total**
Not interested	30	11	23	64
Too busy	11	12	11	34
No reason given	9	10	8	27
Too ill	5	5	9	19
Too well	3	1	2	6
**Total**	**58**	**39**	**53**	**150**

For peer review only

Supplementary File 3 Demographic characteristics of SO participants

Demographic characteristic	Seizure First Aid Training + TAU	Treatment as usual (TAU)	Total
Relationship with patient participant	(n=18)	(n=19)	(n=37)
Parent	4 (22.2%)	4 (21.1%)	8 (21.6%)
Son / daughter	2 (11.1%)	4 (21.1%)	6 (16.2%)
Grandparent	0 (0.0%)	0 (0.0%)	0 (0.0%)
Spouse / partner	9 (50.0%)	7 (36.8%)	16 (43.2%)
Sibling	1 (5.6%)	0 (0.0%)	1 (2.7%)
Cousin, aunt / uncle	0 (0.0%)	0 (0.0%)	0 (0.0%)
Niece / nephew	0 (0.0%)	1 (5.3%)	1 (2.7%)
Friend	2 (11.1%)	3 (15.8%)	5 (13.5%)
Other	0 (0.0%)	0 (0.0%)	0 (0.0%)
Missing	0 (0.0%)	0 (0.0%)	0 (0.0%)
Co-habitation with patient participant	(n=18)	(n=19)	(n=37)
Yes	14 (77.8%)	14 (73.4%)	28 (75.7%)
No	4 (22.2%)	5 (26.3%)	9 (24.3%)
Missing	0 (0.0%)	0 (0.0%)	0 (0.0%)
Contact with patient participant (number of days per week)	(n=18)	(n=19)	(n=37)
None	0 (0.0%)	0 (0.0%)	0 (0.0%)
One	1 (5.6%)	0 (0.0%)	1 (2.7%)
Two	0 (0.0%)	1 (5.3%)	1 (2.7%)
Three	0 (0.0%)	2 (10.5%)	2 (5.4%)
Four	0 (0.0%)	0 (0.0%)	0 (0.0%)
Five	0 (0.0%)	0 (0.0%)	0 (0.0%)
Six	0 (0.0%)	0 (0.0%)	0 (0.0%)
Seven (every day)	17 (94.4%)	16 (84.2%)	33 (89.2%)
Missing	0 (0.0%)	0 (0.0%)	0 (0.0%)
Sex: n (%)	(n=18)	(n=19)	(n=37)
Male	6 (33.3%)	9 (47.4%)	15 (40.5%)
Female	12 (67.7%)	10 (52.6%)	22 (59.5%)
Missing	0 (0.0%)	0 (0.0%)	0 (0.0%)
Age at consent into the trial (years)			
N	17 (94.4%)	19 (100.0%)	36 (94.6%)
Mean	41.3	44.9	43.2
Standard deviation	18.66	15.69	17.01
Minimum	17.8	18.1	17.8
Median	43.5	49.7	48.0
Maximum	79.7	71.5	79.7
Missing	1 (5.6%)	0 (0.0%)	1 (2.7%)
Ethnicity: n (%)	(n=18)	(n=19)	(n=37)
White	18 (100.0%)	18 (94.5%)	36 (97.3%)
Asian / Asian British	0 (0.0%)	0 (0.0%)	0 (0.0%)
Black / African / Carribean / Black British	0 (0.0%)	0 (0.0%)	0 (0.0%)
Mixed / multiple ethnic groups	0 (0.0%)	0 (0.0%)	0 (0.0%)
Other ethnic group	0 (0.0%)	1 (5.3%)	1 (2.7%)
Missing	0 (0.0%)	0 (0.0%)	0 (0.0%)
Significant medical history: n (%)	(n=18)	(n=19)	(n=37)
No, none	13 (72.2%)	12 (63.2%)	25 (67.6%)
Yes, a medical condition	5 (27.8%)	6 (31.6%)	11 (29.7%)
Yes, a psychiatric condition	0 (0.0%)	0 (0.0%)	0 (0.0%)
Yes, both medical and psychiatric conditions	0 (0.0%)	1 (5.3%)	1 (2.7%)
Missing	0 (0.0%)	0 (0.0%)	0 (0.0%)

Education: n (%)	(n=18)	(n=19)	(n=37)
O' levels/ GCSEs/ Level 1 or 2 NVQ	9 (50.0%)	14 (73.7%)	23 (62.2%)
A' Levels/ Level 3 NVQ	4 (22.2%)	2 (10.5%)	6 (16.2%)
University degree/ Graduate Certificate or Diploma	5 (27.8%)	3 (15.8%)	8 (21.6%)
Postgraduate university degree (e.g., PGCE, MSc, MA, PhD)	0 (0.0%)	0 (0.0%)	0 (0.0%)
Missing	0 (0.0%)	0 (0.0%)	0 (0.0%)

For peer review only

Supplementary File 4 Measure completeness by assessment point, measure and participant type

Table S4.1 Number of patient participants completing assessment tools

Study assessment tool and timepoint	SAFE + TAU	TAU	Total
**Patient Participants: Baseline (T0)**	**(n=26)**	**(n=25)**	**(n=51)**
ED visits (self-report)	24 (92.3%)	21 (84.0%)	45 (88.2%)
Seizure control: Thapar scale	26 (100%)	25 (100%)	51 (100%)
Quality of Life: QOLIE-31P	23 (88.5%)	16 (64.0%)	39 (76.5%)
Distress: HADS [a]	23 (88.5%)	24 (96.0%)	47 (92.2%)
Felt stigma: Stigma of Epilepsy Scale	24 (92.3%)	24 (96.0%)	48 (94.1%)
Health economics: Client Service Receipt Inventory [b]	19 (73.1%)	17 (68.0%)	36 (70.6%)
Health economics: EuroQol-5D	21 (80.8%)	25 (100.0%)	46 (90.2%)
Confidence managing seizures/ epilepsy: Epilepsy Mastery Scale	26 (100.0%)	21 (84.0%)	47 (92.2%)
Fear of seizures:, Epilepsy Knowledge and Management Questionnaire Fears subscale	16 (61.5%)	9 (36.0%)	25 (49.2%)
Knowledge of what to do: Thinking about Epilepsy Questionnaire	26 (100%)	25 (100%)	51 (100%)
**Patient Participants: 6 months (T2)**	**(n=26)**	**(n=23)[c]**	**(n=49)[c]**
Quality of Life: QOLIE-31P	16 (61.5%)	12 (52.2%)	28 (57.1%)
Confidence managing seizures/ epilepsy: Epilepsy Mastery Scale	21 (80.8%)	15 (65.2%)	36 (73.5%)
Seizure control: Thapar scale	16 (61.5%)	15 (65.2%)	28 (57.1%)
**Patient Participants: 12 months (T3)**	**(n=25)[d]**	**(n=22)[d]**	**(n=47)[d]**
ED visits (self-report)	17 (68.0%)	17 (77.3%)	34 (72.3%)
Seizure control: Thapar scale	20 (80.0%)	14 (60.9%)	34 (72.3%)
Quality of Life: QOLIE-31P	10 (40.0%)	8 (36.4%)	18 (38.3%)
Distress: HADS [a]	18 (72.0%)	17 (77.3%)	35 (74.4%)
Felt stigma: Stigma of Epilepsy Scale	18 (72.0%)	17 (77.3%)	35 (74.4%)
Health economics: Client Service Receipt Inventory [b]	13 (52.0%)	10 (45.5%)	23 (48.9%)
Health economics: EuroQol-5D	18 (72.0%)	17 (77.3%)	35 (74.4%)
Confidence managing seizures/ epilepsy: Epilepsy Mastery Scale	18 (72.0%)	16 (72.7%)	34 (72.3%)
Fear of seizures: Epilepsy Knowledge and Management Questionnaire Fears subscale	8 (32.0%)	5 (22.7%)	13 (27.7%)
Knowledge of what to do: Thinking about Epilepsy Questionnaire	18 (72.0%)	17 (77.3%)	35 (74.4%)
Feedback on participation	17 (68.0%)	15 (68.2%)	32 (68.1%)

Notes:

[a] Completeness of the whole HADS scale (both anxiety and depression subscales)

[b] Only six mandatory questions counted. Conditional questions (e.g. if yes, then…) not counted towards total completion

[c] Two patient participants from the TAU group had withdrawn by the T2 visit

[d] Four patient participants (three from the TAU group and one from the SAFE + TAU group) had withdrawn by the T3 visit

[e] Note that whilst some participants did not fully complete all items within a questionnaire, this does not automatically mean their data would need to be excluded from analysis of a change in that domain since for some measures test developers permit imputation when the extent of missing items is low and follows a specific pattern.

For peer review only

Table S4.2 Number of SO participants completing study assessment tools

Study assessment tool and time point	SAFE + TAU	TAU	Total
**Significant other Participants: Baseline (T0)**	**(n=18)**	**(n=19)**	**(n=37)**
Burden: Zarit Caregiver Burden	17 (94.4%)	17 (89.5%)	34 (91.9%)
Distress: HADS [a]	17 (94.4%)	18 (94.7%)	35 (94.6%)
Confidence managing seizures/ epilepsy: Parent Response to Child Illness Scale	18 (100.0%)	19 (100.0%)	37 (100.0%)
Fear of seizures: Epilepsy Knowledge and Management Questionnaire Fears subscale	6 (33.3%)	4 (21.1%)	10 (27.0%)
Knowledge of what to do: Thinking about Epilepsy Questionnaire	18 (100.0%)	19 (100.0%)	37 (100.0%)
**Significant other Participants: 6 months (T2)**	**(n=17)[b]**	**(n=16)[b]**	**(n=33)[b]**
Burden: Zarit Caregiver Burden	15 (88.2%)	8 (50.0%)	23 (69.7%)
Confidence managing seizures/ epilepsy: Parent Response to Child Illness Scale	16 (94.1%)	9 (56.3%)	25 (75.8%)
**Significant other Participants: 12 months (T3)**	**(n=17)[c]**	**(n=15)[c]**	**(n=32)[c]**
Burden: Zarit Caregiver Burden	11 (64.7%)	10 (66.7%)	21 (65.6%)
Distress: HADS [a]	11 (64.7%)	10 (66.7%)	21 (65.6%)
Confidence managing seizures/ epilepsy: Parent Response to Child Illness Scale	11 (64.7%)	10 (66.7%)	21 (65.6%)
Fear of seizures: Epilepsy Knowledge and Management Questionnaire Fears subscale	6 (35.3%)	2 (13.3%)	8 (25.0%)
Knowledge of what to do: Thinking about Epilepsy Questionnaire	15 (88.2%)	13(86.7%)	28 (87.5%)
Feedback on participation	11 (64.7%)	9 (60.0%)	20 (62.5%)

Notes:

[a] Completeness of the whole HADS scale (both anxiety and depression subscales),

[b] Four SO participants (one in the SAFE group and three in the TAU group) had withdrawn by the T2 visit

[c] Five SO participants (one in the SAFE group and four in the TAU group) had withdrawn by the T3 visit

[d] Note that whilst some participants did not fully complete all items within a questionnaire, this does not automatically mean their data would need to be excluded from analysis of a change in that domain since for some measures test developers permit imputation when the extent of missing items is low and follows a specific pattern.

For peer review only

Supplementary File 5 Histogram of number of emergency department attendances in 12 prior to randomisation by n=48 patient participants according to routine data

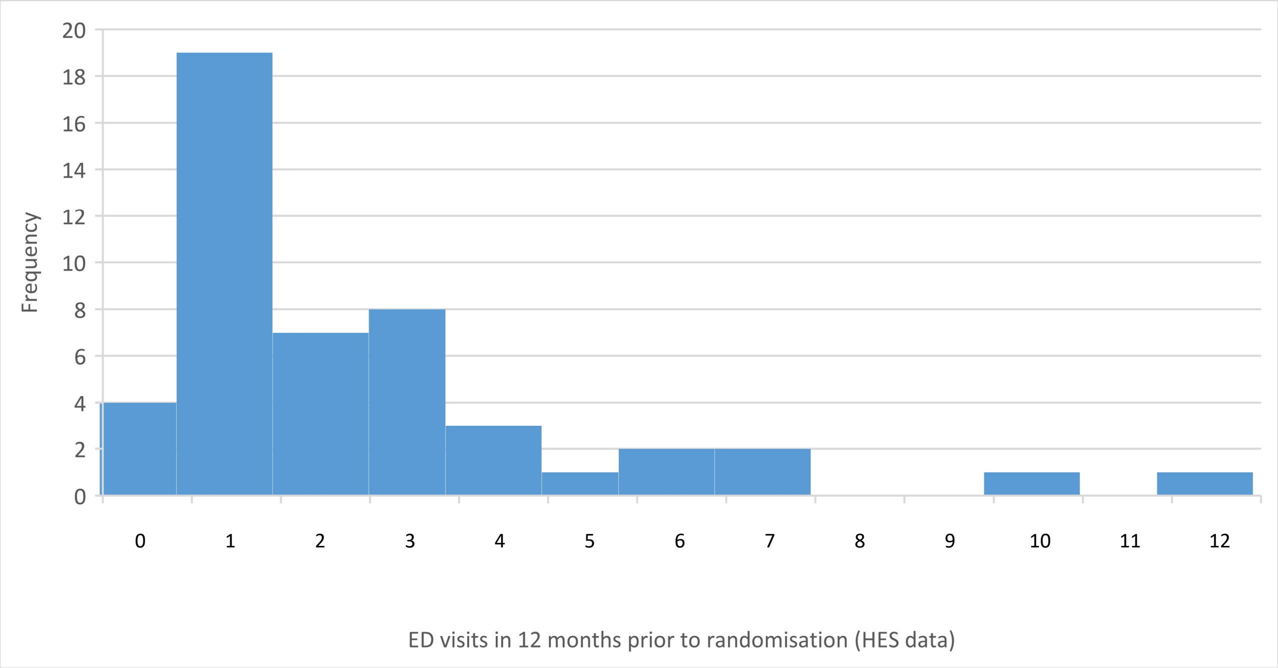

review only

Supplementary File 6 Summary of key milestones in securing ED outcome data from NHS Digital

	**Project milestone**		**Milestone in obtaining data from NHS Digital**	
	Date	*Note*	*Date*	*Note*
**Period**			**28 Sep 2015**	Received feedback on draft Participant Information Sheets and Consent form for trial to ensure compliance.
Trial period	**19 May 2016**	First patient participant randomised		
			**11 Oct 2017**	Participated in NHS Digital training.
			**18 Oct 2017**	Registered as new user of application system.
			**07 Nov 2017**	Delays experienced. Access to application form granted after chasing.
	**Dec 2017**	Final 12-month follow-up completed		
Post -trial			**16 Feb 2018**	Application submitted to NHS Digital
			**28 Feb 2018**	Teleconference with NHS Digital. Provisional opinion given that only minor changes required and likely timeframe to receipt of data 1.5 months.
			**07 Mar 2018**	Received written feedback from NHS Digital after chasing on minor changes required.
			**22 Mar 2018**	Revised application submitted.
			**03 Apr 2018**	NHS Digital submit new query (1) to applicants requesting additional information. (Applicants respond 03 Apr 2018)
			**26 Apr 2018**	NHS Digital submit new query (2) to applicants requesting additional information. (Applicants respond 26 Apr 2018)
			**30 Apr 2018**	NHS Digital submit new query (3) to applicants requesting additional information. (Applicants respond 30 Apr 2018)
			**05 May 2018**	NHS Digital submit new query (4) to applicants requesting additional information. (Applicants respond 05 May 2018).
			**10 May 2018**	NHS Digital submit new query (5) to applicants requesting additional information (in light of new General Data Protection Regulation introduced on May 2018). (Applicants respond 11 May 2018)
			**30 May 2018**	NHS Digital submit new query (6) to applicants requesting additional information (in light of new General Data Protection Regulation introduced on May 2018). (Applicants respond 30 May 2018)
			**30 May 2018**	NHS Digital submit new query (7) to applicants requesting additional information. (Applicants respond 30 May 2018)
			**15 Jun 2018**	NHS Digital confirm Data Approvals Owner will review revised application.
			**27 Jun 2018**	NHS Digital submit new query (8) to applicants requesting additional information and request revisions to trial website in light of new General Data Protection Regulation introduced on May 2018). (Applicants respond 28 Jun 2018)

			**02 Jul 2018**	NHS Digital submit new query (9) to applicants requesting additional information.<br>(Applicants 02 Jul 2018)
			**06 Jul 2018**	NHS Digital notify applicants that Data Approval Owner has rejected application, primarily because: “concerns [over] whether this pilot would yield findings that were statistically valuable to achieve the stated aims given the small numbers”.
			**10 Jul 2018**	Secured confirmation of right to appeal and process.
			**20 Jul 2018**	Applicants submit letter of appeal.
			**25 Jul 2018**	Appeal accepted by NHS Digital.
			**15 Aug 2018**	NHS Digital submit new query (10) to applicants following Independent Group Advising on the Release of Data (IGARD) committee’s review of application.<br>(Applicants respond 16 Aug 2018)
			**16 Aug 2018**	Teleconference with NHS Digital data production team.
			**04 Sep 2018**	NHS Digital send Data Sharing Agreement to applicants.
			**10 Sep 2018**	NHS Digital confirm receipt of completed Data Sharing Agreement.
			**25 Sep 2018**	Applicants securely transfer patient participants’ details to NHS Digital.
			**31 Sep 2018**	NHS Digital release data to applicants.

Supplementary File 7 Adverse events occurring during pilot trial in descending order according to frequency overall [a]

**Adverse event**		**SAFE + TAU**		**TAU**		**Total**	
Category of events (e.g. body system)	Event	Events (n)	Patients [n(%)]	Events (n)	Patients [n(%)]	Events (n)	Patients [n(%)]
Eyes, ear, nose, throat	Problem with eyes and sinuses	0	0 (0.0%)	1	1 (9.1%)	1	1 (5.6%)
Genito-urinary	Overnight hospital admission required due to urinary tract infection (pre existing)	0	0 (0.0%)	1	1 (9.1%)	1	1 (5.6%)
Haematological	Dislocated shoulder	0	0 (0.0%)	1	1 (9.1%)	1	1 (5.6%)
Neoplasia	Change in seizure pattern [b]	1	1 (14.3%)	0	0 (0.0%)	1	1 (5.6%)
	Increase in seizure frequency [b]	1	1 (14.3%)	0	0 (0.0%)	1	1 (5.6%)
Neurological	Increased seizures, medications changed	1	1 (14.3%)	3	3 (27.3%)	4	4 (22.2%)
	Increased seizures (shift patterns at work)	1	1 (14.3%)	0	0 (0.0%)	1	1 (5.6%)
	New seizure type; frequent absence seizures as well as usual seizure types	1	1 (14.3%)	0	0 (0.0%)	1	1 (5.6%)
	Seizure frequency sodium levels requiring in patient monitoring	0	0 (0.0%)	1	1 (9.1%)	1	1 (5.6%)
	Seizures more severe (tonic clonic)	0	0 (0.0%)	1	1 (9.1%)	1	1 (5.6%)
	Vagus Nerve Stimulation not working properly, going to hospital for observation	0	0 (0.0%)	1	1 (9.1%)	1	1 (5.6%)
Respiratory	Increases number of seizures due to chest infection and antibiotics	0	0 (0.0%)	1	1 (9.1%)	1	1 (5.6%)
Other:	Gynae-pregnancy. Increase in the number of fits due to being pregnant / just given birth	0	0 (0.0%)	2	1 (9.1%)	2	1 (5.6%)
	Diagnosis of status epilepticus	1	1 (14.3%)	0	0 (0.0%)	1	1 (5.6%)
	Vertigo	0	0 (0.0%)	1	1 (9.1%)	1	1 (5.6%)
**Total**		6	6 (100.0%)	13	11 (100.0%)	19	18 (100.0%)

Notes:

[a] Eighteen (35.3%) patient participants reported adverse events (AE) over the course of follow-up. In the SAFE+TAU group, 6 AEs were reported by 6 participants, while 13 AEs were reported by 12 participants from the TAU group. Only one SAE occurred during the study – namely, a diagnosis of previous status epilepticus. This was reported by one SAFE+TAU group participant. A medical review asserted it unlikely to be related to participation as it arose from investigations the participant had been having before participation.

[b] Two participants were under investigation to determine whether a brain tumour could be the cause of their seizure changes; hence 'Body System' is classified as 'Neoplasia' rather than 'Neurological.'

For peer review only - http://bmjopen.bmj.com/site/about/guidelines.xhtml

Supplementary File 8 Total observed costs and cost per delegate to deliver the seizure first aid intervention (SAFE) within a research trial with two academic researchers, one Epilepsy Specialist Nurse delivering the training, and administrative support.

**Description**	**Design & set-up**	**Practice Session 1**	**Practice Session 2**	**SAFE+TAU Group 1**	**SAFE+TAU Group 2**	**SAFE+TAU Group 3**	**SAFE+TAU Group 4**	**SAFE+TAU Group 5**	**SAFE+TAU Group 6**	**SAFE+TAU Group 7**	**TAU Group 1**	**TAU Group 2**
Fixed costs (£)												
Equipment	928.85	0.00	0.00	0.00	0.00	0.00	0.00	0.00	0.00	0.00	0.00	0.00
Website	77.12	0.00	0.00	0.00	0.00	0.00	0.00	0.00	0.00	0.00	0.00	0.00
Freepost licence	116.40	0.00	0.00	0.00	0.00	0.00	0.00	0.00	0.00	0.00	0.00	0.00
Venue	0.00	80.00	80.00	80.00	80.00	80.00	80.00	80.00	80.00	80.00	80.00	80.00
Facilitator (staff cost)		192.79	192.79	177.96	177.96	177.96	177.96	177.96	177.96	177.96	177.96	177.96
Facilitation resources		4.96	4.96	4.96	4.96	4.96	4.96	4.96	4.96	4.96	4.96	4.96
Total (fixed)	1122.37	277.75	277.75	262.92	262.92	262.92	262.92	262.92	262.92	262.92	262.92	262.92
Variable costs (£)												
Staff costs	4243.41	612.88	1158.56	591.19	521.84	429.37	429.37	318.41	318.41	318.41	299.96	416.03
Staff travel expenses	44.10	279.87	279.87	274.67	274.67	265.43	265.43	265.43	265.43	265.43	233.63	265.41
Participant expenses	0.00	180.00	200.00	40.00	40.00	40.00	40.00	40.00	40.00	40.00	92.80	0.00
Office costs (inc. telephone)	589.33	33.00	105.50	81.63	85.23	92.60	88.70	89.00	86.60	89.00	57.17	70.13
Stationery, print, post	0.00	156.35	196.96	57.44	93.68	68.93	68.93	34.46	22.98	45.95	182.73	298.44
Refreshments	0.00	72.25	72.25	41.80	41.80	32.81	32.00	41.00	26.60	18.50	21.00	34.50
Advertising	45.00	0.00	0.00	0.00	0.00	0.00	0.00	0.00	0.00	0.00	0.00	0.00
Total (variable)	4921.84	1334.34	2013.13	1086.73	1057.22	929.13	924.42	788.30	760.01	777.29	887.28	1084.51
TOTAL COST (£)	6044.21	1612.09	2290.88	1349.65	1320.14	1192.05	1187.34	1051.22	1022.93	1040.21	1150.20	1347.43
Delegate (n) [patient:SO]		12	15	~~2:~~3:2	4:4	3:3	4:2	3:0	1:1	2:2	4:4	8:5

For peer review only

Mean cost/delegate (£)		134.34	152.73	269.93	165.02	198.68	197.89	350.41	511.47	260.05	143.78	103.65
Mean cost per group (sd)<br>95% central range *				£1,166 (134.22)<br>£1,023-£1,350							£1,249 (139.46)<br>£1,150-£1,347	
Mean cost per delegate<br>95% central range*				£240<br>£211-£278							£119<br>£110-£128	
Mean cost per patient (with or without SO)<br>95% central range*				£408<br>£358-£472							£208<br>£192-£225	

Notes:

*Central ranges generated from 10,000 bootstrap replications; The quantity of use of the resources, was measured. To capture the resources used the 'time and motion method' was used with AN, DS, JB and GM completing electronic data collection forms on which they self-reported the time they dedicated to different activities. The research team (AN, DS) also recorded details of the number of participants invited and attending each SAFE sessions and non-staff resources.

For peer review only

Supplemental File 9 Serious Adverse Event Protocol

Adapted from the Medicines for Human Use (Clinical Trials) Regulations 2004 (SI 2004/1031), http://www.legislation.gov.uk/uksi/2004/1031/pdfs/uksi_20041031_en.pdf [accessed 29th August, 2019] a serious adverse event (SAE) was defined as an adverse event which resulted in any of the following:

- Death
- Was life-threatening (subject at immediate risk of death, e.g., status epilepticus)
- Seizure resulting in hospital admission for ≥24 hours
- Emergency attendance or hospital admission for reason other than seizure
- Results in persistent or significant disability or incapacity, or
- Was otherwise considered medically significant.

'Life-threatening' in the definition of 'serious' referred to an event in which the patient was at risk of death at the time of the event; it did not refer to an event which hypothetically might have caused death if it were more severe. Hospitalisations for a pre-existing condition, including elective procedures that had not worsened, did not constitute an SAE. Prolongation of hospital stay due to social factors, for example, geographical location of the participant's home which prevented discharge was also not considered a SAE.

Given the characteristics of the subject population being studied, the following events were expected in this study population and were not recorded as part of the SAE monitoring process:

- Epileptic seizures with or without injury;
- Emergency or urgent medical attention. This includes visiting a hospital emergency department with the duration of the stay lasting <24 hours, attending an NHS out of hours primary care service (NHS Urgent Care 24), telephoning for an ambulance, telephoning NHS 111, seeking/ having an urgent/ fast-tracked appointment with a usual care provider (GP or specialist) or other registered health professional (e.g., a pharmacist);
- Side-effects of AED medication;
- Diagnosis of a comorbid psychiatric condition.

A delegated medically qualified person within the team (AM; LR) assessed each unexpected SAE. This person considered information on the temporal and physical relationship between the event and possible causes and assessed whether the event was related or unrelated to the patient's participation in the study. Further details on the process followed detailed below.

Monitoring

As part of this trial patient participants did not receive additional medical reviews. There is also no 'live' system which can be used to track SAEs such as emergency admissions and usual care providers are not systematically informed of them. Therefore, to monitor SAEs the research team liaised with patient participants themselves. A standardised form was to be completed at 3- (T1, by telephone), 6- (T2, by telephone) and 12-months (T3, during a face-to-face appointment) post-randomisation to collect information on patient participants' experience of unexpected, SAEs.

In each instance, a maximum of 3 attempts was made to contact the patient participant by telephone (including trying to contact them via their informal carer if they are taking part with one). If the patient was

For peer review only

not be contactable, a letter was be sent the patient's GP asking them to inform the research team if the patient was no longer alive and the circumstances of their death.

Causality

One of the delegated medically qualified persons within the team assessed each unexpected SAE. They considered information on the temporal and physical relationship between the event and possible causes and assessed whether the event was related or unrelated to the patient's participation in the study. In doing this, they used the definitions in the Table below.

Table Definitions of causality for serious adverse event

	**Description**
**Unrelated**	There is no evidence of any causal relationship. There is an alternative cause for the SAE.
**Unlikely**	There is little evidence to suggest there is a causal relationship (e.g. the event did not occur within a reasonable time after receipt of the intervention). There is another reasonable explanation for the event (e.g. the participant's clinical condition, other concomitant treatment).
**Possibly**	There is some evidence to suggest a causal relationship (e.g. because the event occurs within a reasonable time after receipt of the intervention). However, the influence of other factors may have contributed to the event (e.g. the participants clinical condition, other concomitant treatment).
**Probably**	There is evidence to suggest a causal relationship and the influence of other factors is unlikely.
**Almost certainly**	There is clear evidence to suggest a causal relationship and other possible contributing factors can be ruled out.

Reporting

As per National Research Ethics Service (2015) guidelines for non-CTIMPs, the main ethics committee approving the study and its Sponsor were informed within 15 days of the team becoming aware of any SAE that in the opinion of the medical reviewer was both unexpected (that is, the type of event is not listed in the protocol as an expected occurrence) and judged to be "possibly", "probably" or "almost certainly" related to participation in the study (that is, it resulted from administration of any of the research procedures, including the intervention).

CONSORT 2010 checklist of information to include when reporting a pilot or feasibility trial*

Section/Topic	Item No	Checklist item	Reported on page No
**Title and abstract**			
	1a	Identification as a pilot or feasibility randomised trial in the title	1
	1b	Structured summary of pilot trial design, methods, results, and conclusions (for specific guidance see CONSORT abstract extension for pilot trials)	2
**Introduction**			
Background and objectives	2a	Scientific background and explanation of rationale for future definitive trial, and reasons for randomised pilot trial	4-5
	2b	Specific objectives or research questions for pilot trial	5
**Methods**			
Trial design	3a	Description of pilot trial design (such as parallel, factorial) including allocation ratio	6 (sec. 2.2), 7 (sec.2.6)
	3b	Important changes to methods after pilot trial commencement (such as eligibility criteria), with reasons	6 (sec. 2.2)
Participants	4a	Eligibility criteria for participants	6-7 (sec. 2.2)
	4b	Settings and locations where the data were collected	6 (sec. 2.2)
	4c	How participants were identified and consented	6 (sec. 2.4) & Supplementary File 1
Interventions	5	The interventions for each group with sufficient details to allow replication, including how and when they were actually administered	7 (sec. 2.8), Table 1, and references 32 & 37.
Outcomes	6a	Completely defined prespecified assessments or measurements to address each pilot trial objective specified in 2b, including how and when they were assessed	7-8 (sec. 2.9)
	6b	Any changes to pilot trial assessments or measurements after the pilot trial commenced, with reasons	N/A.
	6c	If applicable, prespecified criteria used to judge whether, or how, to proceed with future definitive trial	7 (sec. 2.9)
Sample size	7a	Rationale for numbers in the pilot trial	9 (sec. 2.10)
	7b	When applicable, explanation of any interim analyses and stopping guidelines	N/A.
Randomisation:			

For peer review only

Sequence generation	8a	Method used to generate the random allocation sequence	7 (sec.2.6)
	8b	Type of randomisation(s); details of any restriction (such as blocking and block size)	7 (sec.2.6)
Allocation concealment mechanism	9	Mechanism used to implement the random allocation sequence (such as sequentially numbered containers), describing any steps taken to conceal the sequence until interventions were assigned	7 (sec.2.6)
Implementation	10	Who generated the random allocation sequence, who enrolled participants, and who assigned participants to interventions	7 (sec.2.6)
Blinding	11a	If done, who was blinded after assignment to interventions (for example, participants, care providers, those assessing outcomes) and how	7 (sec.2.6)
	11b	If relevant, description of the similarity of interventions	N/A.
Statistical methods	12	Methods used to address each pilot trial objective whether qualitative or quantitative	8-9 (sec. 2.10)
**Results**			
Participant flow (a diagram is strongly recommended)	13a	For each group, the numbers of participants who were approached and/or assessed for eligibility, randomly assigned, received intended treatment, and were assessed for each objective	9-10 (sec. 3.1), 10-11 (sec. 3.3), Figure 1
	13b	For each group, losses and exclusions after randomisation, together with reasons	10 (3.1), Figure 1
Recruitment	14a	Dates defining the periods of recruitment and follow-up	6 (sec. 2.1, 2.2)
	14b	Why the pilot trial ended or was stopped	N/A.
Baseline data	15	A table showing baseline demographic and clinical characteristics for each group	Table 4
Numbers analysed	16	For each objective, number of participants (denominator) included in each analysis. If relevant, these numbers should be by randomised group	Tables 4, 5, 6 and Supplementary File
Outcomes and estimation	17	For each objective, results including expressions of uncertainty (such as 95% confidence interval) for any estimates. If relevant, these results should be by randomised group	9-13 (sec. 3)
Ancillary analyses	18	Results of any other analyses performed that could be used to inform the future definitive trial	Supplementary Files
Harms	19	All important harms or unintended effects in each group (for specific guidance see CONSORT for harms)	12 (3.7), Supplementary File 9
	19a	If relevant, other important unintended consequences	N/A
**Discussion**			

For peer review only

Limitations	20	Pilot trial limitations, addressing sources of potential bias and remaining uncertainty about feasibility	16 (sec. 4.4)
Generalisability	21	Generalisability (applicability) of pilot trial methods and findings to future definitive trial and other studies	16 (sec. 4.4)
Interpretation	22	Interpretation consistent with pilot trial objectives and findings, balancing potential benefits and harms, and considering other relevant evidence	13-15 (sec. 4)
	22a	Implications for progression from pilot to future definitive trial, including any proposed amendments	15 (sec. 4.3)
**Other information**			
Registration	23	Registration number for pilot trial and name of trial registry	1
Protocol	24	Where the pilot trial protocol can be accessed, if available	6 (reference 34)
Funding	25	Sources of funding and other support (such as supply of drugs), role of funders	17
	26	Ethical approval or approval by research review committee, confirmed with reference number	6 (sec. 2.3)

Citation: Eldridge SM, Chan CL, Campbell MJ, Bond CM, Hopewell S, Thabane L, et al. CONSORT 2010 statement: extension to randomised pilot and feasibility trials. BMJ. 2016;355.
*We strongly recommend reading this statement in conjunction with the CONSORT 2010, extension to randomised pilot and feasibility trials, Explanation and Elaboration for important clarifications on all the items. If relevant, we also recommend reading CONSORT extensions for cluster randomised trials, non-inferiority and equivalence trials, non-pharmacological treatments, herbal interventions, and pragmatic trials. Additional extensions are forthcoming: for those and for up to date references relevant to this checklist, see www.consort-statement.org.

For peer review only

Seizure First Aid Training For people with Epilepsy (SAFE) frequently attending emergency departments and their significant others: results of a UK multi-centre randomised controlled pilot trial

Journal:	*BMJ Open*
Manuscript ID	bmjopen-2019-035516.R2
Article Type:	Original research
Date Submitted by the Author:	19-Feb-2020
Complete List of Authors:	Noble, Adam; University of Liverpool, Department of Health Services Research<br>Snape, Dee; University of Liverpool, Department of Health Services Research<br>Nevitt, Sarah; University of Liverpool, Department of Biostatistics<br>Holmes, EA; Centre for Health Economics & Medicines Evaluation<br>Morgan, Myfanwy; King's College London, Institute of Pharmaceutical Science<br>Tudur-Smith, Catrin; University of Liverpool, Biostatistics<br>Hughes, Dyfrig; Bangor University, Centre for Health Economics and Medicines Evaluation<br>Buchanan, Mark; Wirral University Teaching Hospital NHS Foundation Trust, Emergency Department, Arrowe Park Hospital<br>McVicar, Jane; Mackinnon Memorial Hospital, NHS Highland,<br>MacCallum, Elizabeth; Aintree University Hospital, Liverpool University Hospitals NHS Foundation Trust, Emergency Department<br>Goodacre, Steve; University of Sheffield, Medical Care Research Unit<br>Ridsdale, Leone; King's College London, Department of Basic and Clinical Neuroscience, Institute of Psychiatry, Psychology & Neuroscience<br>Marson, Anthony; University of Liverpool, Molecular and Clinical Pharmacology
**Primary Subject Heading**:	Neurology
Secondary Subject Heading:	Health services research, Emergency medicine, Health economics
Keywords:	Epilepsy < NEUROLOGY, ACCIDENT & EMERGENCY MEDICINE, Organisation of health services < HEALTH SERVICES ADMINISTRATION & MANAGEMENT, Health economics < HEALTH SERVICES ADMINISTRATION & MANAGEMENT, Clinical trials < THERAPEUTICS

SCHOLARONE™
Manuscripts

I, the Submitting Author has the right to grant and does grant on behalf of all authors of the Work (as defined in the below author licence), an exclusive licence and/or a non-exclusive licence for contributions from authors who are: i) UK Crown employees; ii) where BMJ has agreed a CC-BY licence shall apply, and/or iii) in accordance with the terms applicable for US Federal Government officers or employees acting as part of their official duties; on a worldwide, perpetual, irrevocable, royalty-free basis to BMJ Publishing Group Ltd ("BMJ") its licensees and where the relevant Journal is co-owned by BMJ to the co-owners of the Journal, to publish the Work in this journal and any other BMJ products and to exploit all rights, as set out in our licence.

The Submitting Author accepts and understands that any supply made under these terms is made by BMJ to the Submitting Author unless you are acting as an employee on behalf of your employer or a postgraduate student of an affiliated institution which is paying any applicable article publishing charge ("APC") for Open Access articles. Where the Submitting Author wishes to make the Work available on an Open Access basis (and intends to pay the relevant APC), the terms of reuse of such Open Access shall be governed by a Creative Commons licence – details of these licences and which Creative Commons licence will apply to this Work are set out in our licence referred to above.

Other than as permitted in any relevant BMJ Author's Self Archiving Policies, I confirm this Work has not been accepted for publication elsewhere, is not being considered for publication elsewhere and does not duplicate material already published. I confirm all authors consent to publication of this Work and authorise the granting of this licence.

For peer review only

TITLE: Seizure First Aid Training For people with Epilepsy (SAFE) frequently attending emergency departments and their significant others: results of a UK multi-centre randomised controlled pilot trial

AUTHORS: A.J. Noble,[1*] D Snape[1], S Nevitt,[2] EAF Holmes,[3] M Morgan,[4] C Tudur Smith,[2] DA Hughes,[3,10] M Buchanan,[5] J McVicar,[6] E MacCallum,[7] S Goodacre,[8] L Ridsdale, [9] AG Marson.[10]

AFFILIATIONS:

[1] Department of Health Services Research, University of Liverpool, Liverpool, UK

[2] Liverpool Clinical Trials Centre, Department of Biostatistics, University of Liverpool, Liverpool, UK

[3] Centre for Health Economics and Medicine Evaluation, Bangor University, Bangor, UK

[4] Institute of Pharmaceutical Science, King's College London, London, UK

[5] Emergency Department, Arrowe Park Hospital, Wirral University Teaching Hospital NHS Foundation Trust, Wirral, UK.

[6] Mackinnon Memorial Hospital, Broadford, NHS Highland, Isle of Skye, UK.

[7] Emergency Department, Aintree University Hospital, Liverpool University Hospitals NHS Foundation Trust

[8] School of Health and Related Research, Sheffield University, Sheffield, UK

[9] Department of Basic and Clinical Neuroscience, King's College London, London, UK

[10] Department of Molecular and Clinical Pharmacology, University of Liverpool, Liverpool, UK

FOR CORRESPONDENCE: Name: Adam Noble PhD; Address: University of Liverpool, Department of Health Services Research, Waterhouse Building, Liverpool, L69 3GB, United Kingdom.; Telephone: +44 151 794 5993; E-mail: adam.noble@liverpool.ac.uk

For peer review only

ABSTRACT

Objective: To determine the feasibility and optimal design of a randomised controlled trial (RCT) of Seizure First Aid Training For Epilepsy (SAFE).

Design: Pilot RCT with embedded microcosting.

Setting: Three English hospital emergency departments (EDs).

Participants: Patients aged ≥16 with established epilepsy reporting ≥2 ED visits in the prior 12 months and their significant others (SO).

Interventions: Patients (and their SO) were randomly allocated (1:1) to SAFE plus Treatment-As-Usual (TAU) or TAU alone. SAFE is a 4-hour group course.

Main outcome measures: Two criteria evaluated a definitive RCT's feasibility: i) ≥20% of eligible patients needed to be consented into the pilot; ii) routine data on use of ED over the 12-months post-randomisation needed securing for ≥75%. Other measures included: eligibility; ease of obtaining routine data; availability of self-report ED data and comparability; SAFE's effect; and intervention cost.

Results: Of ED attendees with a suspected seizure, 424 (10.6%) were eligible; 53 (12.5%) patients and 38 SOs consented. Fifty-one patients (and 37 SOs) were randomised. Routine data on ED use at 12-months was secured for 94.1%. Self-report ED data was available for 66.7%. Patients reported more visits compared to routine data. Most (76.9%) randomised to SAFE received it and no related serious adverse events occurred. ED use at 12 months was lower in the SAFE+TAU arm compared to TAU alone, but not significantly (Rate Ratio= 0.62, 95% confidence interval 0.33 to 1.17). A definitive trial would need ~674 patient participants and ~39 recruitment sites. Obtaining routine data was challenging, taking ~8.5 months.

Conclusions: In satisfying only one predetermined 'stop/go' criterion, a definitive RCT is not feasible. The low consent rate in the pilot raises concerns about a definitive trial's findings external validity and means it would be expensive to conduct. Research is required into how to optimise recruitment from the target population.

Trial registration number: ISRCTN13 871 327.

For peer review only - http://bmjopen.bmj.com/site/about/guidelines.xhtml

'Strengths and limitations of this study' (5 bullet points; one sentence each, relating to the methods)

1. Randomisation was done remotely by computer and stratification factors and allocations concealed from those collecting baseline and follow-up data and analysing it.
2. Participants were recruited from sites serving areas where social deprivation was high and epilepsy control poor and so similar to those where a definitive RCT would likely need to focus recruitment.
3. We completed one of the few microcostings of a self-management intervention for epilepsy.
4. Despite recruited patient participants stating sufficient ED use when screened, routine data subsequently suggested ~40% did not meet the inclusion criteria.
5. We estimated the effect of SAFE only on the proposed primary outcome measure for a definitive RCT.

KEY WORDS: Epilepsy; Accident & Emergency Medicine; Organisation of health services; Heath economics; Clinical Trials.

Words: 3,955

.

For peer review only

For peer review only - http://bmjopen.bmj.com/site/about/guidelines.xhtml

1. BACKGROUND

International evidence shows a significant minority of people with epilepsy (PWE) frequently use hospital emergency departments (EDs).[1-4] In the United Kingdom (UK), around 20% of PWE visit each year, half are admitted [5-7]. Whilst the exact distribution of use amongst attendees is unclear,[8-10] ~60% may make several visits each year.[11]

Emergency hospital use by PWE has been identified as an area for potential cost savings [12] as whilst expensive – ~£70-90M in England each year [8 13 14] – it is often of little clinical benefit since most attendees have known, rather than new epilepsy and experienced uncomplicated seizures.[9 15-17] Guidance states such seizures could be managed by PWE and their significant others (SOs).[18 19] Indeed, iatrogenic harms may arise in seeking emergency care for them.

PWE visiting EDs have a characteristic profile. They report more seizures, anxiety, poorer quality of life and are more likely to live in socially deprived areas compared to the wider epilepsy population.[11 20-23] They therefore share some of the characteristics of those at increased risk of epilepsy associated death.[24 25] In the UK, PWE visiting EDs can also be challenging to identify for research since most (~62%) are not being followed-up by an epilepsy specialist,[1] primary care providers are also not always notified of ED visits by their patients [9] and because EDs do not always code the reason for an attendance.[26]

Whilst the focus on PWE attending EDs is welcomed, it needs to be translated into care improvements. Although a range of promising interventions have been suggested,[27] assessment of their utility is lacking.[28]

One intervention proposed is seizure first aid training. It has potential as PWE frequently visiting EDs and their SOs (to whom care decisions can be delegated [29 30]) express particularly low knowledge and confidence in this domain, are fearful of seizures, and there are indications this leads them to seek emergency medical attention for seizures, rather than self-managing them.[31]

As no such intervention existed, we developed one – called Seizure First Aid Training For Epilepsy (SAFE). SAFE is a manualised group-based self-management course

For peer review only - http://bmjopen.bmj.com/site/about/guidelines.xhtml

(Table 1). It's rationale and development has been reported.[32] In brief, it was co-designed with health professionals, patients and carers. It was developed for delivery to groups of up to 10 patient-carer dyads by a single facilitator with knowledge of epilepsy and lasts for ~4 hours. It contains 6 modules centred around basic epilepsy and first aid knowledge, the recovery position, informing others about epilepsy and how to help if seizures occur, medical identification, seizure triggers and home safety. Materials include presentation slides and professionally produced videos. Its behaviour change potential has been optimised by course recipients completing a Self-Affirmation exercise at the start.

SAFE's efficacy now needs testing. A randomized controlled trial (RCT) would be an appropriate methodology for this. ED use over the 12 months following randomisation could be the primary outcome. It is unknown though whether such a trial is feasible and what its optimal design should be.

Specifically, will patients and their SOs take part, attend SAFE, and be willing and able to be followed-up? It is also not possible to calculate the required sample size because the distribution of ED use is unclear. These uncertainties exist because SAFE is newly developed and no RCT has attempted to recruit the target population.

It is also unclear how to measure ED visits. Funders are encouraging trialists to use routinely recorded data to assess outcomes. In England, routine data on a person's ED use is recorded within the Hospital Episode Statistics system. This data's cost, how long it takes to obtain and its comparability to self-report is though unknown.

In such circumstances, guidelines highlight the importance of pilots.[33] Their primary focus is not effect, but judging feasibility and providing information to optimise a definitive RCT's design.[34] We thus completed a pilot RCT comparing SAFE, plus treatment-As-Usual (TAU) to TAU alone. It had the following objectives, which will be relevant to those interested in SAFE and those researching this population:

i) Estimate likely eligibility, consent, and retention rates in a definitive RCT;

For peer review only - http://bmjopen.bmj.com/site/about/guidelines.xhtml

ii) Estimate annual rate of ED visits in TAU group and the likely dispersion parameter;

iii) Determine feasibility of measuring ED use by routine data;

iv) Estimate completion rates of study assessment tools;

v) Estimate rates of researcher unblinding;

vi) Provide summary statistics to estimate effect of SAFE on ED use and its precision;

vii) Capture patient and SOs views on trial participation;

viii) Estimate the intervention's cost.

2. METHODS

The trial's protocol has been published.[31] Here we provide a brief overview.

2.1 Design

Parallel arm, multicentre, external pilot RCT. Assessments with participants on the definitive trial's proposed primary and secondary outcome measures were performed at baseline prior to randomization (T0) and 12-months later (T3) (Table 2). Interim assessments occurred at 3 (T1) and 6-months (T2).

SAFE was offered to the TAU alone group after T3 assessments were completed.

2.2 Trial setting

Three hospitals in North-West England – which serve populations featuring high social deprivation [35 36] and emergency admissions for epilepsy [22] – were recruitment sites (Acknowledgements). From May to December 2016 patients were invited who had attended any of these hospital's ED over the prior 12 (and with governance approval, later extended to 18) months for epilepsy (see 2.4).

2.3 Ethical considerations and approvals

The National Research Ethics Service Committee (15/NW/0225) and Health Research Authority (166241) approved the study. An independent Trial Steering Committee monitored it.

2.4 Patient recruitment

For peer review only - http://bmjopen.bmj.com/site/about/guidelines.xhtml

ED clinicians searched their hospitals' attendance record systems for potential participants (Supplementary File 1), screened their triage cards and posted invitations to eligible patients. Recipients had 3 weeks to return an opt-out response if not interested in participating. Those not opting-out were telephoned by a researcher to verify eligibility and willingness to participate. Patients taking part (and their SO if they chose to take part with one) provided informed consent at an enrolment appointment with a researcher (DS). For patients, this included consent to access their routine data.

2.5 Eligibility criteria

Table 3 details the criteria. In brief, patients were eligible if they were aged ≥16, diagnosed with epilepsy, prescribed antiepileptic drugs, could give informed consent and, when telephoned, self-reported ≥2 ED visits for epilepsy in the prior 12 months.

2.6 Randomization and blinding

Patients (and their SOs) were randomised (1:1) by an online system managed by the Liverpool Clinical Trials Centre. It used a minimisation program with a built-in random element and two stratification factors (recruitment site and whether the patient reported epilepsy stigma at baseline).

Usual care provider(s) and DS, who was responsible for recruitment and follow-up, were blinded to allocations and stratification factors. Participants could not be blinded and so were asked not to reveal their allocation to DS.

Staff (GM) organizing attendance at SAFE were not involved in data collection and not blinded. The trial (SN) statistician and senior statistician (CTS) were blinded until the database was 'locked'.

2.7 Measures to assess patient and SOs participants' outcomes

Table 2 details these measures.

2.8 Intervention

An epilepsy nurse specialist (JB) delivered SAFE within a local hospital's educational centre. A fidelity assessment found they delivered SAFE with excellent protocol adherence and competence.[37]

For peer review only - http://bmjopen.bmj.com/site/about/guidelines.xhtml

2.9 Outcomes

To achieve the study objectives, rates of eligibility, consent and retention were calculated. Retention being the percentage of patients for which 12-month primary outcome data was secured. Two a priori progression criteria helped judge feasibility: i) ≥20% of eligible patients were recruited; ii) 12-month primary outcome data secured for ≥75% of patients.

By assessing participants on the proposed outcome measures, we obtained estimates of the distribution of ED use, measure completeness and SAFE's effect. To evaluate blinding, DS completed a 'Treatment Guess' form after each participant's T3 assessment or withdrawal. It required her to state which treatment arm she believed the participant had been randomised to. The proportion of participants willing to participate in such a trial again was ascertained and experience of adverse events calculated. Time taken to obtain routine data and the fee payable was recorded. To see if self-reported ED visits provided a reliable estimate compared to routine data the agreement between the two data sources on how many ED visits a patient had made was explored.

2.10 Statistical analyses

As this was a pilot RCT, a power calculation was inappropriate. Instead, the sample size was chosen to provide adequate precision to estimate the parameters required.[38]

Based on existing data,[1 14 17 39 40] it was anticipated 12 months of attendances from each ED would allow identification of ~400 eligible patients. With a 20% consent rate, 80 patient participant accruals could be expected. With 80 patient participants we could estimate a potential drop-out rate of 25% to within a 95% confidence interval of +/-10% and a consent rate of 20% to within a 95% confidence interval of +/-4%. Assuming ED data at T3 was not available for 25% of patients, data from 60 patients would still allow robust estimation of the ED rate and dispersion parameter. Sample sizes of 24 to 50 have been recommended as 'adequate' for pilot studies.[38 41]

Analyses were documented a priori in an analysis plan and performed using SAS (9.4). Baseline characteristics for patient and SO participants are described using

For peer review only - http://bmjopen.bmj.com/site/about/guidelines.xhtml

descriptive statistics and patient participants compared, on a subset, to eligible patients declining participation. Parameters are reported with 95% confidence intervals (CIs).

SAFE's effect on ED use, with and without adjustment for number of ED visits prior to randomisation, was estimated using negative binomial regression (NBR) on a modified intention-to-treat basis (as defined by Del Re et al.[42]). Participants were included with their number of ED visits recorded with no data imputation. NBR was the pre-specified statistical approach as over-dispersion (i.e. large variance) was anticipated in the number of ED visits reported. Between-group differences are presented as Rate Ratios and, as per recommendations for hypothesis testing within pilot trials,[43] tested according to 5, 10 and 20% levels of significance.

The proportion of correct treatment guesses was determined and Cohen's Kappa computed. Bland-Altman plots compared ED visits as measured by routine data and self-report.[44]

As no consensus exists about what constitutes a clinically important reduction in ED visits,[45] average annual rate of ED visits in the SAFE+TAU and TAU alone groups post randomisation (measured according to routine data) and the likely dispersion parameter from the adjusted NBR model were used to estimate the sample size of a definitive trial using Keene et al.'s [46] formula. According to the formula, the number of patient participants required per group in a definitive trial to detect the size of the effect shown in the pilot study is:

$$n = \left\{ \frac{z_{1-\beta} + z_{1-\alpha/2}}{\log(\mu_1/\mu_2)} \right\}^2 \times \left\{ \frac{\mu_1 + \mu_2}{\mu_1 \mu_2} + 2k \right\}$$

Where $Z_{1-\alpha/2}$ and $Z_{1-\beta}$ are critical values of the Normal distribution for specific values of alpha (α) and power (β). μ_1 and μ_2 are the estimated ED rates from the two treatment groups and k the negative binomial shape parameter from the associated gamma distribution which explicitly represents variability between subjects. For the calculations, alpha was set at 5%, but the dispersion parameter and power required was varied to explore differences in sample size required.

For peer review only - http://bmjopen.bmj.com/site/about/guidelines.xhtml

2.11 Microcosting

Microcosting adopted the perspective of an academic non-profit making institution and conducted in three steps:[47] (1) resource identification; (2) resource use measurement, applying the time and motion method;[48] and (3) valuation using price year 2017/18 for local and national data. Data were analysed using Microsoft Excel to calculate the fixed and variable costs of delivering SAFE. Fixed costs included, equipment, website, freepost licence, venue hire, facilitator staff cost, and facilitation resources; and assumed 11 groups/year and equipment life of 1-year. Variable costs were support staff and office costs, staff and participant travel expenses, and consumables. Total cost per group was calculated as fixed costs plus variable costs, for each group and each arm (SAFE+TAU; TAU). Mean cost per group was calculated as the sum of total costs / number of groups. Mean cost per delegate (or patient only) were calculated as sum of total cost per group in each arm / sum of delegates (or patients only) in each arm. Results are presented as: cost per training session, mean cost per delegate, mean cost per patient. The 95% central range (CR) for costs and differences were generated using Monte Carlo Simulation of 10,000 replications.

2.12 Patient and Public Involvement statement

This research came about as improving education for patients and families on epilepsy is a top research priority.[49] To ensure SAFE was developed and tested in a way that met service users' needs: the 'Epilepsy Society' and patient and SO representatives from the 'Brain and Spine Foundation' helped develop recruitment materials; the pilot was overseen by a Trial Steering Committee including 2 service user representatives; SAFE's was designed by an Intervention Panel including 2 service user representatives and its content informed by feedback from 23 service users;[32] finally, pilot trial participants reported on the burden of participation with a view to optimising the design of a potential definitive RCT.

3. RESULTS

3.1 Participant recruitment, allocation and treatment

Of the 4016 individuals identified, 1220 (30.4%, CI 29.0% to 31.8%) had visited for established epilepsy. Of these, 424 (34.8%, CI 32.1% to 37.4%) were eligible; eligibility rate 10.6% (CI 9.6% to 11.5%) (Figure 1 and Supplementary File 2).

For peer review only - http://bmjopen.bmj.com/site/about/guidelines.xhtml

Of the 424 patients invited, 53 consented; consent rate 12.5% (CI 9.3% to 15.6%). Telephone contact could though only be made with 203 (47.8%, CI 43.1% to 52.6%) patients. The consent rate amongst those who could be contacted was 26.1% (CI 20.0% to 32.2%). The main reasons for 150 patients declining participation were “lack of interest” (42.7%, CI 34.8% to 50.6%) and being “too busy” (22.6%; CI 16.0% to 29.4%).

Of the 53 consenting patients, 51 were randomised (with 37 SOs) (Figure 1 gives reasons for non-randomisation). Of the 51, 26 (and 18 SOs) were allocated to SAFE+TAU and 25 (and 19 SOs) to TAU alone. Most (20, 76.9%) patients and SOs (13, 72.2%) randomized to SAFE+TAU attended a course.

3.2 Participant demographics and epilepsy characteristics

The patient participants mean age was 39.9 years (SD 15.7, range 16-71); 29 (56.9%) were female and most (74.4%) lived in areas high in deprivation (49% in the 10 percent most socially deprived areas in England) (Table 4).

Recruited patients were comparable in age and deprivation to those declining participation. Females were though over-represented (Table 4).

Recruited patients had an epilepsy diagnosis for a median of 17.3 years. Most (62.8%) reported ≥10 seizures in the previous year and having seen a neurologist (74.5%) (Table 4). Participants' mean Quality of Life 31-P score was low at 48.3 (SD 17.3; range 17.1 to 79.5). Twenty-six (50.9%) had ‘probable’ clinical anxiety. Most (n=42, 82.3%) reported feeling stigmatised by epilepsy; 15 (29.4%) highly. The treatment groups were broadly similar in demographics and epilepsy characteristics.

SO participants were typically a partner/spouse (43.2%) and most (89.2%) had daily patient contact (Supplementary File 3). With a mean Zarit Caregiver burden score of 18.9 (SD=12.51), SOs typically reported ‘little or no burden’ [50] but anxiety was high [51]; 15 (40.5%) SOs had ‘probable’ clinical anxiety.

3.3 Participant retention

3.3.1 Proposed primary outcome measure:

Of the randomised patients, 3 (5.8%) formally withdrew over follow-up, removing access to their routine data. Primary outcome data on ED use at 12 months (and for the 12 months prior to randomisation) was available for the remaining 48 patients, giving a retention rate of 94.1%.

3.3.2 Proposed secondary outcome measures:

Thirty-seven (72.5%) randomised patients and 21 (56.8%) SOs attended their 12-months questionnaire appointment (T3). The extent to which measures were completed at these and the interim appointments varied (Supplementary File 4). Self-report data on ED use at T3 was obtained from only 34 patients, giving a retention rate on this measure of 66.7% patients.

3.4 ED use

3.4.1 Baseline, prior to randomisation:

Routine data for the 48 patients for whom consent was maintained showed they made 122 ED visits in the 12 months before randomisation (Supplementary File 5). The mean was 2.5 and median 2 (range 0 to 12). ED use was slightly higher for TAU participants (Table 5).

Despite only consenting patients who when telephoned reported ≥2 ED visits in the prior 12 months, routine data indicated 4 (8.3%) had not made any visits during the prior 12 months. A further 19 (39.6%) made only one.

3.4.2 At 12 months, effect of SAFE:

Compared to the 12-months prior to randomisation, mean ED use over follow-up reduced for the SAFE+TAU group from 2.1 visits to 1.8 (difference -0.3). For the TAU group, it increased from 3 visits to 3.4 (difference 0.4) (Table 5). Unadjusted NBR estimated the visit rate was 46% lower in the SAFE+TAU group compared to the TAU group (Rate Ratio= 0.54; Vuong's test z=-0.17, P=0.87). In the adjusted model, SAFE's effect reduced (Rate Ratio= 0.62). Between group differences were not significant at the 5% or 10% alpha level in either the unadjusted or adjusted model. The effect was significant in both at the 20% level (Table 6). The dispersion parameter under the adjusted NBR model was k=0.69 (CI 0.17 to 1.21).

For peer review only - http://bmjopen.bmj.com/site/about/guidelines.xhtml

3.5 Obtaining routine data and its correspondence with self-report

Routine data took 8.5 months to secure, arriving ~9 months after T3 assessments finished. The direct cost was £6960. Supplementary File 6 shows substantial work on behalf of the research team to obtain the data was required. In one instance, an appeal against a decision to reject the application – 5 months in – was necessary.

Routine data did not match patient self-report in most cases. Forty-two patients had self-report and routine data on ED use at baseline. Only 3 (7.1%) reported the same number of visits as their routine data indicated. Most (76.2%) reported more (by 3.8 visits on average) (Figure 2a). There was greater agreement between routine and self-report data at T3; 11 (32.4%) patients reported the same number of visits as their routine data (Figure 2b).

3.6 Blinding

The researcher correctly guessed which of the two treatment arms35 patient participants had been allocated to by the randomisation process; unblinding rate 68.6% (CI 54.1% to 80.9%). The chance-corrected kappa statistic of 0.37 (CI 0.12 to 0.63) equated to "fair" agreement.

3.7 Safety

No serious adverse events related to participation occurred (Supplementary File 7).

3.8 Participant feedback

Thirty-two (68.1%) patients and 20 (62.5%) SOs completed the T3 feedback questionnaire. All but one said they would participate in such a trial again; participants indicated they perceived benefits from SAFE.

3.9 Sample size calculation

Based on the estimated effect of SAFE (see 3.4.2), Table 7 shows the number of patient participants required *per* group for a definitive trial. If the central value in the estimate range for the dispersion parameter *k* of 0.69 is used and 90% power stipulated, then a total starting sample of 674 patient participants (i.e., [308*2]+58) would need to be recruited. This accounts for the 9.4% of recruited patients who (on

For peer review only - http://bmjopen.bmj.com/site/about/guidelines.xhtml

the basis of the pilot trial) would be anticipated to withdraw consent to access their routine ED data. In the pilot, 5 of the 53 patients recruited withdraw consent.

3.10 Microcosting

Delivering SAFE was estimated to cost £333 (CR £288 to £380) per patient (with or without a SO). When including the cost SAFE's development (£181 per person), the mean cost per *attendee*, based on all 55 participants in SAFE+TAU or TAU groups who ultimately attended a SAFE session, was £375 (CR £348 to £402) (Supplementary File 8). This reduced to £194 (CR £167 to £221) when excluding sunk costs. Staff time accounted for 50.01% of the cost of SAFE's delivery. SAFE's facilitator was not local to the trial area. Thus, an analysis of facilitator costs without travel expenses (time and cost), reduced the mean cost by 21.08% from £194 (CR £167 to £221) to £152 (CR £124 to £179) per delegate (£261 per patient with or without SO). The annual fixed cost of setting-up and running SAFE was £1122 (+£35.07 per patient, based on 32 patients/year attending with or without SO).

4. DISCUSSION

The pilot was successful, providing estimates of key parameters, including recruitment and retention. An informed decision regarding feasibility and optimal design of a definitive trial of SAFE can now be made.

4.1 Positives from pilot for feasibility and design of definitive trial

4.1.1 Identification, treatment and safety:

The pilot indicates it is possible to identify, consent, randomise and safely treat persons from the target population within a definitive trial. This was not a given since no RCT had focused recruitment on them.

4.1.2 Retention and measuring ED use:

By using routine data as the basis of the primary outcome measure, a definitive trial should not be affected by attrition. It permitted 12-month data to be secured for 94% of pilot participants. Despite having shorter-follow-ups (6 months), 151 definitive RCTs funded by the NIHR secured outcome data for 89% of patients.[52] The use of routine data in our pilot meant it satisfied the retention progression criterion. It would not have

For peer review only - http://bmjopen.bmj.com/site/about/guidelines.xhtml

been met if self-report data were relied on (only 67% of patients provided it). This leads us to recommend ED use be measured using routine data.

Another reason for the recommendation is its cost would be low compared to employing staff to obtain self-report data. Routine data would also prevent a definitive trial from exposure to apparent recall bias, with patients seemingly over reporting ED use (it has been asserted bias is not an issue for ‘‘big ticket’’ service items).[53 54]

Our pilot does though caution that provision of routine data is slow and not straightforward. It took 8.5 months to obtain. In principle, such applications should be processed within 2 months.[55] Data providers should attend to such issues as those funding and designing trials need confidence data can be secured and realistic estimates for when. It is also worth noting that some of the ED visits attributed to our participants may not have been epilepsy related. Since a diagnostic code is not recorded for ~35% of ED visits, we asked NHS Digital to inform us of any ED visits recorded for our participants.[26]

4.2 Negatives from pilot for feasibility and design of definitive trial

4.2.1 Consent:

Only a small proportion of identified patients were eligible (10.6%) and willing (12.5%) to take part, meaning the progression criterion that ≥20% of eligible patients agree to participate was not met.

The progression criterion was stated a priori because when uptake is so low there is the real possibility that those who are and are not taking part differ. We compared the age and deprivation of patients who did and did not take part and found no obvious differences. We do not know though whether they differed on other indices since access to non-participants' medical records was not ethically permissible. One indication the pilot might not have recruited a representative sample was the high proportion of patients seeing a neurologist in the prior 12 months (~75%). Evidence suggests this should be closer to 38%.[1]

The consent rate also raises questions over the likelihood of a definitive trial being funded since it would make it expensive. On average, each pilot site generated 17.6

For peer review only - http://bmjopen.bmj.com/site/about/guidelines.xhtml

patient accruals from 18 months of attendances. To achieve a sample size of ~674 PWE, a definitive trial could thus require ~39 sites (half of England's EDs). This is 2.5 times more sites than in recent NIHR HTA funded RCTs [52] which already had a mean cost of ~£1.3M.[56] A definitive RCT of SAFE might thus not represent acceptable value for money to funders.[57]

Epilepsy and its consequences (e.g., seizures, memory difficulties, no driving license) makes recruiting PWE challenging. The consent rate in the pilot is nevertheless low. In the largest RCT of self-management (SMILE trial), 37% of the people with uncontrolled epilepsy invited took part.[58] The characteristics of the patients from ED agreeing to participate in our pilot suggests one reason for the low uptake might be stigma; 82% felt some (21.6% felt highly stigmatised). This is higher than in the wider epilepsy population; 63% of SMILE's sample felt stigmatised (12.5% highly).[59] Stigma can make PWE feel ashamed and reluctant to talk about epilepsy. This could explain why the target population is so challenging to recruit. Unfortunately, it is unclear how to revise recruitment to mitigate against this.

Evidence-based strategies were employed in the pilot to maximise recruitment,[31] and invitation materials co-produced with patients. It is generally considered preferable though for a usual care provider, with whom a patient has an established relationship, to invite a person into research. Difficulties identifying the target population (see 1.0) meant we had local ED clinicians do the inviting. A future trial might consider asking EDs to identify ostensibly eligible patients, but for the GPs of the identified patients to do the inviting. This may boost recruitment.[60]

4.2.2 Effect of intervention:

Our pilot estimated SAFE's effect to be modest (reducing ED visits from 2.1 over 12-months to 1.8). This has negative consequences for a definitive trial, not least because it requires it to have a large sample to detect the effect. Efficacy was not though our pilot's primary focus and the estimate may be imprecise. Indeed, it might be that those who declined to participate in the trial and who appeared to differ in some important ways, might have benefited more. Previous evidence does though suggest it is in the region expected.

For peer review only - http://bmjopen.bmj.com/site/about/guidelines.xhtml

Specifically, only one RCT – by Sajatovic et al.[61] – has considered change in ED use following epilepsy self-management.[62] Conducted in the U.S. it compared SMART – an 8-session group intervention – to wait-list control. No significant change between groups was found in subsequent ED/ hospitalization use. For SMART, it reduced from a mean of 1.22 by .44 over the 6 months following randomisation. For the controls it reduced from 2.4 by -1.26 visits.

4.3 Judgement regarding progression to definitive trial

Thabane et al.[63] provide a framework for judging whether to progress to a definitive trial. In satisfying only one progression criteria, a definitive trial based on the pilot's design is not feasible. We have also not identified any modifications that will make it feasible. We therefore recommend not proceeding.

4.4 Strengths and limitations

Strengths include the pilot being reported according to guidelines,[64] allocations being concealed, that patients were recruited from sites similar to those likely for a definitive trial and we included a microcosting of SAFE's delivery.

The pilot is not without potential weaknesses. Most importantly, despite recruited patient participants stating sufficient ED use when screened, routine data subsequently suggested ~40% did not meet the inclusion criteria. This could limit our findings external validity and attenuate SAFE's effect. Another potential limitation is we estimated the effect of SAFE only on the proposed primary measure. We did not estimate its wider effects, including on duration of ED visits and admissions.

5. CONCLUSION

A definitive trial of SAFE is not currently feasible. Research is required to determine how people from the target population can be better recruited.

For peer review only - http://bmjopen.bmj.com/site/about/guidelines.xhtml

ACKNOWLEDGEMENTS: We thank the people who kindly participated in this study and the local ED sites for their support – namely, Arrowe Park Hospital (Wirral University Teaching Hospital NHS Foundation Trust), and Aintree University Hospital and the Royal Liverpool University Hospitals (Liverpool University Hospitals NHS Foundation Trust). Other persons who contributed to this work are: Mrs. Juliet Bransgrove [JB] (epilepsy nurse specialist), Dr Duncan Appelbe (website development/ management) and Ms. Gail Moors [GM] (Administrator). We also acknowledge the support we received from our Steering Committee (Prof. Alasdair Gray [chair], Prof. Pete Bower, Dr. Paul Cooper, Mr. Mike Jackson, Ms. Helen Coyle, Mr. Mike Perry, Mrs. Linda Perry, Mrs. Jayne Burton and Mr. Sam Burton). The study sponsor was the University of Liverpool (reference: UoL001108) and the Clinical Trials Research Centre's registration number is 12.

AUTHORS CONTRIBUTIONS: AJN and LR conceived of the study and designed it together with AGM, CTS, DH, MM and SG. SJN and CTS planned and conducted the statistical analysis, EH and DH the economic analysis. AJN was the chief investigator, with MB, JM and EM leading participant identification and DS recruitment and follow-up. AJN wrote the manuscript, with revisions and approval of the final manuscript coming from all authors.

FUNDING STATEMENT: This project was funded by the National Institute for Health Research's Health Services and Delivery Research Programme (HS&DR Programme) (project number 14/19/09). The views and opinions expressed herein are those of the authors and do not necessarily reflect those of the University of Liverpool, the HS&DR programme, the NIHR, the NHS, or the Department of Health and Social Care.

ew only

COMPETING INTERESTS: None declared.

Competing interests: None declared.

PATIENT CONSENT FOR PUBLICATION: Not required.

DATA STATEMENT: Requests for anonymised data should be submitted to the corresponding author.

For peer review only - http://bmjopen.bmj.com/site/about/guidelines.xhtml

Table 1 SAFE course and its content

Overview

SAFE was developed to be delivered to groups of ~10 patient-SO dyads by a single facilitator with epilepsy knowledge who follows a treatment manual. Materials include standardised presentation slides, videos and interactive tasks. Patients are given information packs that include copies of the slides, wallet sized first aid instructions cards, paper epilepsy ID Cards and instructions for setting up IDs on smart-phone, and access to a website with the intervention's content on.

SAFE's behaviour change potential is supported by reference to Self-Affirmation Theory.

SAFE's order of presentation and content is as follows:

Order	Topics	Learning activity	Minutes allotted
1	Welcome	Slide	5
2	Goals of this course	Slide	2
3	What would you like from today?	Interactive	20
4	True or false?	Interactive	12
5	Taking on information (Kindness Questionnaire)	Interactive	10
6	Epilepsy, seizures & how the brain works	Video	10
7	First aid for convulsive seizures	Interactive	10
8	What can you do to help someone during a seizure?	Slide	5
9	What not to do during a seizure	Slide	5
10	What to do after the seizure has stopped	Slide	5
11	Questions or comments?	Interactive	10
12	Post-seizure states	Slide	15
13	Injuries	Slide	2
14	When to call an ambulance?	Slide	10
15	Questions or comments?	Slide	10
16	Refreshment break	Networking	10
17	Recovery position	Slide	2
18	Recovery position	Video	2
19	Let's practice the recovery position	Interactive	15
20	Questions or comments?	Interactive	5
21	Who needs to know how to help?	Interactive	5
22	What they need to know & why	Slide	5

For peer review only - http://bmjopen.bmj.com/site/about/guidelines.xhtml

23	How to get this information to them. Family, friends & work colleagues:	Slide	5
24	How to get this information to them. Members of the public and health workers:	Slide	5
25	Questions or comments?	Interactive	5
26	Refreshment break	Networking	5
27	Personal stories - introduction	Slide	2
28	Ben's story (case vignette)	Slide	6
29	How to change what happened to Ben? (Carrying medical ID; triggers)	Interactive	5
30	Triggers	Slide	5
31	Knowing your triggers	Slide	4
32	Some ways of dealing with triggers	Slide	4
33	Sandra's story (case vignette)	Slide	6
34	How to change what happened to Sandra (Warning signs; home safety)	Interactive	2
35	Main points to remember, If you have epilepsy:	Slide	3
36	Main points to remember; If you know someone with epilepsy:	Slide	2
37	Sources of further information	Slide	2
38	What's on the back table and accessing the study website*	Slide	2
39	Questions or comments?	Slide	2
40	Evaluation		-
41	Certificates of attendance		-
**Time in minutes:** 240			

Notes:

* http://www.seizurefirstaid.org.uk/Intervention/

For peer review only - http://bmjopen.bmj.com/site/about/guidelines.xhtml

Table 2 Proposed primary and secondary outcome measures for a definitive trial used within pilot by assessment and participant type [a]

Outcome	Domain	Participants	Measure and derivation of outcome	Baseline (T0)	3-month (T1)	6-month (T2)	12 month (T3)
Primary	ED visits	PWE	*Routine data, Hospital Episode Statistics Accident & Emergency system*<br>NHS numbers of patient participants securely transferred to NHS Digital who hold the Hospital Episode Statistics system. NHS Digital then provided the number of ED visits these patients were recorded as having made in the 12 months prior to and in the 12 months following their randomisation. [b]	✓	-	-	✓
Secondary	ED visits	PWE	*Self-report, "How many times have you used Casualty / Accident & Emergency (A&E) over the past 12 months for epilepsy?"*<br>(For T2 the time frame was 6 months)<br>Provided with seizure diary at T0 to assist.	✓	-	✓	✓
	Seizure control	PWE	Baseline: *Thapar's*[65] *Seizure Frequency Scale* for the prior 12 months.<br>T2 and T3: Asked for number of epileptic seizures (of any type) since last assessment and dates of the first and most recent. Provided with seizure diary at T0 to assist.	✓	-	✓	✓
	Quality of life	PWE	*Quality of Life in Epilepsy Scale-31-P (31 items)* [66]	✓	-	✓	✓
	Distress	PWE; SOs	*Hospital Anxiety and Depression Scale (14 items)* [67] [c]	✓	-	-	✓
	Felt Stigma	PWE; SOs	*Stigma of Epilepsy Scale (9 items)* [68] [d]	✓	-	-	✓
	Burden	SOs	*Zarit Caregiver Burden Inventory (22 items)* [50] [e]	✓	-	✓	✓
	Adverse events	PWE	*Monitored by PWE completing a checklist.* [f]	-	✓	✓	✓
	Fear of seizures	PWE; SOs	*Epilepsy Knowledge and Management Questionnaire – Fears subscale (5 items)* [69]	✓	-	-	✓

For peer review only - http://bmjopen.bmj.com/site/about/guidelines.xhtml

	Knowledge of what to do when faced with a seizure	PWE; SOs	*Thinking About Epilepsy Questionnaire (13 items)*[70]	✓	-	-	✓
	Confidence managing seizures/ epilepsy	PWE; SOs	PWE: *Epilepsy Mastery Scale (6 items)*[71]<br>SOs: *Parents Response to Child Illness Scale- Condition Management subscale* (6 items)[72]	✓	-	✓	✓
	Health economics	PWE	*Client Service Receipt Inventory*[73] and *EQ-5D* (13 items)[74]	✓	-	-	✓
-	Feedback on trial participation	PWE; SOs	Adapted from Magpie Trial (3 items)[75]<br>(1) "If time suddenly went backward, and you had to do it all over again, would you agree to participate in the Seizure First Aid Training trial?";<br>(2) "Please tell us if there was anything about the Seizure First Aid Training Trial that you think could have been done better";<br>(3) "Please tell us if there was anything about the Seizure First Aid Training Trial, or your experience of joining the trial, that you think was particularly good"	-	-	-	✓

Notes:

ED= Emergency Department; PWE= Person with epilepsy; SOs= Significant others.

[a] Unless a patient formally withdrew consent to participate in the trial, routine data on their ED use was requested and up to 3 attempts were made to contact patients or SO participant each time one of their follow-up assessments was due.

[b] To minimise cost, a single application for routine data was submitted, rather than one relating to the baseline period and one for follow-up.

[c] Total anxiety and total depression scores were categorised according to the following: 0 to 7, 'Normal range'; 8 to 10, 'Suggestive of anxiety / depression'; 11 to 21, 'Probable anxiety / depression.'

[d] Total stigma scores were categorised as follows: 0, 'No stigma'; 1 to 6, 'Mildly to moderately stigmatized'; 7 to 9, 'Highly stigmatized;

[e] Total Zarit Burden scores were categorised as follows: 0 to 20, 'Little or no burden', 21 to 40, 'Mild to moderate burden', 41 to 60, 'Moderate to severe burden'; 61 to 88, 'Severe burden.'

[f] Definitions of serious adverse event and how relatedness was assessed is presented in Supplementary File 9.

For peer review only

Table 3 Participant inclusion and exclusion criteria

Participants	Inclusion criteria	Exclusion criteria
Patients	• Established diagnosis of epilepsy (≥1 year);<br>• All epilepsy syndromes and all types of focal and generalised seizures;<br>• Currently being prescribed antiepileptic medication;<br>• Age ≥16 years (no upper age limit);<br>• Visited an ED for epilepsy on ≥2 occasions within the previous 12 months (as reported by patient);<br>• Live within 25 miles of any of the ED recruitment sites;<br>• Able to provide informed consent, participate in intervention and independently complete questionnaires in English.	• Actual or suspected psychogenic non-epileptic seizures alone or in combination with epilepsy;<br>• Acute symptomatic seizures related to acute neurological illness or substance misuse;<br>• Severe current psychiatric disorders (e.g. acute psychosis) or life-threatening medical illness;<br>• Enrolled in other epilepsy related non-pharmacological treatment studies.
Carers	• A significant other to the patient (e.g., family member, friend) who the patient identifies as providing informal support;<br>• Age ≥16 years (no upper age limit);<br>• Lives in the North West area of England	• Severe current psychiatric disorders or life threatening medical illness;<br>• Enrolled in other epilepsy related non-pharmacological treatment studies.

Notes:

Patient participants were permitted to take part without a carer. Carers could not, however, take part without a patient partner having at least consented to take part into the study.

For peer review only

For peer review only - http://bmjopen.bmj.com/site/about/guidelines.xhtml

Table 4 Characteristics of eligible patients who did and did not agree to participate

Variable		Agreed to participate *SAFE + TAU* (n=26)	Agreed to participate *TAU* (n=25)	Agreed to participate *Combined* (n=51)	Did not agree to participate (n=379)[b]
Sex: n (%)					
Male		10 (38.5%)	12 (48.0%)	22 (43.1%)	192 (50.7%)
Female		16 (61.5%)	13 (52.0%)	29 (56.9%)	187 (49.3%)
Age at presentation to emergency department (years)					
Mean		39.2	40.7	39.9	40.6
Standard deviation		13.96	17.52	15.66	16.83
Median (min., max.)		37.1 (18.9, 69.9)	41.4 (16.5, 71.3)	38.8 (16.4, 71.3)	37.5 (16.2, 84.4)
Index of Multiple Deprivation (IMD) [a]					
IMD Decile: 1	n (%)	11 (42.3%)	14 (56.0%)	25 (49.0%)	188 (49.6%)
IMD Rank	Median (min., max.)	574.0 (44, 3202)	1231.5 (48, 2166)	673.0 (44, 3202)	924.5 (28, 3107)
IMD Decile: 2 to 3	n (%)	7 (26.9%)	6 (24.0)	13 (25.4%)	82 (21.6%)
IMD Rank	Median (min., max.)	6785 (4649, 8281)	6665 (3989, 7816)	6785 (3989, 8281)	5309 (3291,9835)
IMD Decile: 4 to 6	n (%)	2 (7.7%)	4 (16.0%)	6 (11.8%)	59 (15.6%)
IMD Rank	Median (min., max.)	12836 (9881, 15791)	11924 (11480, 16004)	11924 (9881, 16004)	14459 (10277, 19659)
IMD Decile: 7 to 10	n (%)	5 (19.2%)	1 (4.0%)	6 (11.8%)	50 (13.2%)
IMD Rank	Median (min., max.)	27642 (24971, 31002)	32724 (32724, 32724)	28876 (24971, 32724)	22673 (19826, 32396)
	Missing	1 (3.8%)	0 (0.0%)	1 (2.0%)	0 (0.0%)
Ethnicity: n (%)					
White		25 (96.2%)	23 (92.0%)	48 (94.1%)	-
Asian / Asian British		0 (0.0%)	0 (0.0%)	0 (0.0%)	-
Black / African / Caribbean / Black British		1 (3.8%)	1 (4.0%)	2 (3.9%)	-
Mixed / multiple ethnic groups		0 (0.0%)	0 (0.0%)	0 (0.0%)	-
Other ethnic group		0 (0.0%)	1 (4.0%)	1 (2.0%)	-
Other significant medical history: n (%)					
No, none		13 (50.0%)	10 (40.0%)	23 (45.1%)	-
Yes, another medical condition/s		10 (38.5%)	13 (52.0%)	23 (45.1%)	-

For peer review only - http://bmjopen.bmj.com/site/about/guidelines.xhtml

Yes, a psychiatric condition	1 (3.8%)	0 (0.0%)	1 (2.0%)	-
Yes, both medical and psychiatric conditions	2 (7.7%)	2 (8.0%)	4 (7.8%)	-
Education: n (%)				
O' levels/ GCSEs/ Level 1 or 2 NVQ	13 (50.0%)	14 (56.0%)	27 (53.0%)	-
A' Levels/ Level 3 NVQ	5 (19.2%)	3 (12.0%)	8 (15.7%)	-
University degree/ Graduate Certificate or Diploma	5 (19.2%)	5 (20.0%)	10 (19.6%)	-
Postgraduate university degree (e.g., PGCE, MSc, MA, PhD)	2 (7.7%)	0 (0.0%)	2 (3.9%)	-
Missing	1 (3.9%)	3 (12.0%)	4 (7.8%)	-
Time since epilepsy diagnosis (years)				
Mean	19.9	22.6	21.2	-
Standard deviation	14.85	18.38	16.57	-
Median (mix., max.)	16.8 (1.8, 53.9)	19.3 (1.7, 64.9)	17.3 (1.7, 64.9)	-
Time since last epileptic seizure (days)				
Mean	53.6	40.1	47.0	-
Standard deviation	101.10	61.21	83.34	-
Median (min., max.)	14.0 (1, 340)	10.5 (0, 235)	14.0 (0, 340)	-
Missing	3 (11.5%)	3 (12.0%)	6 (11.8%)	-
Number of epileptic seizures in the last 12 months				
0-3	4 (15.3%)	3 (12.0%)	7 (13.8%)	-
4-6	1 (3.8%)	5 (20.0%)	6 (11.7%)	-
7-9	3 (11.5%)	3 (12.0%)	6 (11.7%)	-
10 or more	18 (69.2%)	14 (56.0%)	32 (62.8%)	-

Notes:

[a] The Index of Multiple Deprivation (IMD) ranks every small area in England from 1 (most deprived area) to 32,844 (least deprived area).IMD rank and decile missing for one recruited participant as they resided in Wales.; [b] Includes seven patients who could not be contacted due to an incorrect postal address and two patients who were not sent an invite letter in error.min.= minimum, max.= maximum.

For peer review only - http://bmjopen.bmj.com/site/about/guidelines.xhtml

Table 5 Number of ED visits patient participants made according to routine data

**Number of ED visits**		**SAFE+TAU** (n=26)	**TAU** (n=25)	**Total** (n=51)
In the 12 months to baseline	Mean	2.1	3	2.5
	Standard deviation	2.22	2.76	2.51
	Median (min., max.)	1 (0, 10)	2 (1, 12)	2 (0, 12)
	Missing	1 (3.8%)	2 (8.0%)	3 (5.9%)
In the 12 months following randomisation	Mean	1.8	3.4	2.6
	Standard deviation	3.14	4.78	4.05
	Median (min., max.)	1 (0, 12)	2 (0, 20)	1 (0, 20)
	Missing	1 (3.8%)	2 (8.0%)	3 (5.9%)
Change from baseline over 12 months following randomisation	Mean	-0.3	0.4	0.1
	Standard deviation	1.99	3.81	2.99
	Median (min., max.)	0 (-4, 5)	0 (-5, 16)	0 (-5, 16)
	Missing	1 (3.8%)	2 (8.0%)	3 (5.9%)

For peer review only

Table 6 Between intervention group differences in number of ED visits

**Model and parameter**	**Parameter**	**Confidence Interval (CI)**			**P-value**
		95%	90%	80%	
12 months following randomisation according to routine data[a]					
Negative binomial: SAFE + TAU (Rate Ratio)	0.54	0.24 to 1.18	0.28 to 1.04	0.32 to 0.90	0.12
Negative binomial: Dispersion parameter	1.53	0.67 to 2.39	0.80 to 2.25	0.96 to 2.09	NA
Vuong's Test[b]	-0.17	NA	NA	NA	0.87
12 months following randomisation according to routine data with adjustment for baseline ED visits					
Negative binomial: SAFE + TAU (Rate Ratio)	0.62	0.33 to 1.17	0.36 to 1.06	0.41 to 0.94	0.14
Negative binomial: Baseline ED visits (Rate Ratio)	1.33	1.18 to 1.52	1.20 to 1.49	1.23 to 1.45	<0.001
Negative binomial: Dispersion parameter	0.69	0.17 to 1.21	0.26 to 1.13	0.35 to 1.03	NA
Vuong's Test[b]	-0.13	NA	NA	NA	0.90

Notes:

ED=Emergency Department; SAFE=Seizure First Aid Training; TAU=Treatment-As-Usual,

[a] Analysis based on 48 patient participants

[b] Vuong's test p-value interpretation, a significantly negative parameter value favours the Negative Binomial model and significantly positive favours the Zero-inflated Negative Binomial model. A non-significant value indicates no significant difference between the models therefore the simpler Negative Binomial model is preferred.[76]

For peer review only

Table 7 Required sample size for a definitive trial to detect estimated effect of SAFE+TAU on ED use

Dispersion parameter (*k*)	~47% reduction (from 3.4 to 1.8 visits) in 12 months compared to TAU	
	n per group (α= 0.05; 80% power, β=0.2)	n per group (α= 0.05; 90% power, β=0.1)
0.17	123	164
0.5	191	255
0.69	230	308
1	293	393
1.21	337	451

Notes:
Dispersion parameter taken from the adjusted NBR model in the pilot RCT (i.e., k=0.69; 95% CI: 0.17 to 1.21); *see* Table 6

For peer review only

Figure Legend

Figure 1 CONSORT diagram of flow through the pilot trial

Figure 2 a) Bland Altman plot of agreement between self-reported ED use and routine data on ED use in 12 months prior to randomisation; b) Bland Altman plot of agreement between self-reported ED visits and routine data on ED use over 12 months following randomisation.

For peer review only

For peer review only - http://bmjopen.bmj.com/site/about/guidelines.xhtml

References

1. Dixon PA, Kirkham JJ, Marson AG, et al. National Audit of Seizure management in Hospitals (NASH): results of the national audit of adult epilepsy in the UK. *BMJ Open* 2015;5:e007325.
2. Rizos T, Jüttler E, Sykora M, et al. Common disorders in the neurological emergency room – experience at a tertiary care hospital. *European Journal of Neurology* 2011;18:430–35.
3. Royl G, Ploner CJ, Möckel M, et al. Neurological chief complaints in an emergency roo. *Der Nervenarzt* 2010;81(10):1226-30.
4. Marson T, Taylor C, Tudur-Smith C, et al. Seizure management in hospitals in Europe. ILAE 13th European Congress on Epileptology. Vienna, 2018.
5. Hart YM, Shorvon SD. The nature of epilepsy in the general population. II. Medical care. *Epilepsy research* 1995;21(1):51-58.
6. Jacoby A, Buck D, Baker G, et al. Uptake and costs of care for epilepsy: findings from a UK regional study. *Epilepsia* 1998;39(7):776-86.
7. Kitson A, Shorvon S. Services for patients with epilepsy: Report of a CSAG committee: Department of Health, 2000.
8. Dickson JM, Jacques R, Reuber M, et al. Emergency hospital care for adults with suspected seizures in the NHS in England 2007–2013: a cross-sectional study. *BMJ open* 2018;8(10):e023352.
9. Pearson M, Marson A, Dixon P, et al. National Audit of Seizure Management in Hospitals: St. Elsewhere's Hospital Clinical Report 2014 [Available from: http://www.nashstudy.org.uk/Newsletters/St%20Elsewhere's%20Clinical%20Report%20NASH%202.pdf accessed 9th April 2019.
10. Whiston S, Coyle B, Chappel D. Health needs assessment for long term neurological conditions in North East England. Stockton on Tees: North East Public Health Observatory 2009.
11. Noble AJ, Goldstein LH, Seed P, et al. Characteristics of people with epilepsy who attend emergency departments: Prospective study of metropolitan hospital attendees. *Epilepsia* 2012;53(10):1820–28.
12. NHS England. NHS Outcomes Framework indicators - Feb 2017 release. 2017 [Available from: https://digital.nhs.uk/data-and-information/publications/clinical-indicators/nhs-outcomes-framework/archive/nhs-outcomes-framework-indicators---february-2017-release accessed 8th August 2017.
13. House of Commons Committee of Public Accounts. Services to people with neurological conditions: progress review London House of Commons; 2015 [Available from: https://publications.parliament.uk/pa/cm201516/cmselect/cmpubacc/502/502.pdf accessed 4th April 2019.
14. Ridsdale L, McCrone P, Morgan M, et al. Can an epilepsy nurse specialist-led self-management intervention reduce attendance at emergency departments and promote well-being for people with severe epilepsy? A non-randomised trial with a nested qualitative phase. *Health Services and Delivery Research* 2013;1(9)
15. Dickson JM, Taylor LH, Shewan J, et al. Cross-sectional study of the prehospital management of adult patients with a suspected seizure (EPIC1). *BMJ Open* 2016;6:e010573.

For peer review only - http://bmjopen.bmj.com/site/about/guidelines.xhtml

16. Peter A Dixon, Jamie J Kirkham, Anthony G Marson, et al. National Audit of Seizure management in Hospitals (NASH): results of the national audit of adult epilepsy in the UK *BMJ Open* 2015;5(3):e007325.
17. Reuber M, Hattingh L, Goulding PJ. Epileptological emergencies in Accident and Emergency: a survey at St James's University Hospital, Leeds. *Seizure* 2000;9(3):216-20.
18. NHS Choices. What to do if someone has a seizure (fit) 2017 [Available from: https://www.nhs.uk/conditions/what-to-do-if-someone-has-a-seizure-fit/ accessed 4th April 2019.
19. Epilepsy Action. What to do when someone has a seizure 2016 [Available from: https://www.epilepsy.org.uk/info/firstaid/what-to-do accessed 4th April 2019.
20. Ashworth M, Seed P, Armstrong D, et al. The relationship between social deprivation and the quality of primary care: a national survey using indicators from the UK Quality and Outcomes Framework. *Br J Gen Pract* 2007;57(539):441-48.
21. Bautista RED, Glen ET, Wludyka PS, et al. Factors associated with utilization of healthcare resources among epilepsy patients. *Epilepsy research* 2008;79(2-3):120-29.
22. QIPP/Right Care. The NHS atlas of variation in healthcare 2010 [Available from: fingertips.phe.org.uk/documents/Atlas_2010%20Compendium.pdf.
23. Allard J, Shankar R, Henley W, et al. Frequency and factors associated with emergency department attendance for people with epilepsy in a rural UK population. *Epilepsy & Behavior* 2017;68:192-95.
24. Faught E, Duh MS, Weiner JR, et al. Nonadherence to antiepileptic drugs and increased mortality: findings from the RANSOM Study. *Neurology* 2008;71(20):1572-78.
25. Ridsdale L, Charlton J, Ashworth M, et al. Epilepsy mortality and risk factors for death in epilepsy: a population-based study. *Br J Gen Pract* 2011;61(586):e271-e78.
26. Health and Social Care Information Centre. Hospital Accident and Emergency Activity Supporting Information, 2017-18 2018 [cited Available from: https://files.digital.nhs.uk/8B/94BB9E/AE1718_supporting_information.pdf accessed 27th August 2019.
27. International League Against Epilepsy - British Branch. Epilepsy health services for epilepsy - the proceedings of an expert workshop London2016 [Available from: https://ilaebritish.org.uk/epilepsy-emergency-care/ accessed 22nd October 2019.
28. National Clinical Guideline Centre. Pharmacological Update of Clinical Guideline 20. The Epilepsies: The Diagnosis and Management of the Epilepsies in Adults and Children in Primary and Secondary Care London: National Clinical Guideline Centre (UK); 2012 [Available from: ncbi.nlm.nih.gov/pubmedhealth/PMH0068980.
29. Noble AJ, Morgan M, Virdi C, et al. A nurse-led self-management intervention for people who attend emergency departments with epilepsy: the patients' view. *Journal of Neurology* 2013;260(4):1022–30.
30. Ridsdale L, Virdi C, Noble AJ, et al. Explanations given by people with epilepsy for using emergency medical services: a qualitative study. *Epilepsy Behaviour* 2012;25:529–33.
31. Noble AJ, Marson AG, Tudur-Smith C, et al. 'Seizure First Aid Training' for people with epilepsy who attend emergency departments, and their family and

For peer review only - http://bmjopen.bmj.com/site/about/guidelines.xhtml

friends: study protocol for intervention development and a pilot randomised controlled trial. *BMJ Open* 2015;5:e009040.
32. Snape DA, Morgan M, Ridsdale L, et al. Developing and assessing the acceptability of an epilepsy first aid training intervention for patients who visit UK emergency departments: A multi-method study of patients and professionals. *Epilepsy and Behavior* 2017;68:177-85.
33. Craig P, Dieppe P, Macintyre S, et al. Developing and evaluating complex interventions: the new Medical Research Council guidance. *Bmj* 2008;337:a1655.
34. Lancaster GA, Dodd S, Williamson PR. Design and analysis of pilot studies: recommendations for good practice. *Journal of Evaluation in Clinical Practice* 2004 10(2):307-12.
35. Department of Health. Health Profile 2012. Liverpool. 2012 [Available from: http://fingertipsreports.phe.org.uk/health-profiles/2012/e08000012.pdf accessed 11th April 2018.
36. Department of Health. Health Profile 2012. Wirral 2012 [Available from: http://fingertipsreports.phe.org.uk/health-profiles/2012/e08000015.pdf accessed 11th April 2018.
37. Noble AJ, Snape D, Ridsdale L, et al. Assessing treatment fidelity within an epilepsy randomized controlled trial: Seizure first aid training for people with epilepsy who visit emergency departments. *Behavioural Neurology* 2019;19:1-11.
38. Sim J, Lewis M. 2012. *Journal of Clinical Epidemiology* The size of a pilot study for a clinical trial should be calculated in relation to considerations of precision and efficiency;65:301-08.
39. Digital N. Accident and Emergency Attendances in England - 2012-13 2014 [Available from: https://digital.nhs.uk/data-and-information/publications/statistical/hospital-accident--emergency-activity/accident-and-emergency-attendances-in-england-2012-13 accessed 22nd October 2019.
40. Bhatt H, Matharu MS, Henderson K, et al. An audit of first seizures presenting to an Accident and Emergency Department. *Seizure* 2005;14:58-61.
41. Julious SA. Sample size of 12 per group rule of thumb for a pilot study. *Pharmaceutical Statistics* 2005;4:287–91.
42. Del Re AC, Maisel NC, Blodgett JC, et al. Intention-to-treat analyses and missing data approaches in pharmacotherapy trials for alcohol use disorders. *BMJ Open* 2013;3(11):e003464.
43. Lee EC, Whitehead AL, Jacques RM, et al. The statistical interpretation of pilot trials: should significance thresholds be reconsidered? *BMC Medical Research Methodology* 2014;14:41.
44. Bland JM, Altman D. Statistical methods for assessing agreement between two methods of clinical measurement. *The Lancet* 1986;327:307-10.
45. Noble AJ, Marson AG. Which outcomes should we measure in adult epilepsy trials? The views of people with epilepsy and informal carers. *Epilepsy and Behavior* 2016;59:105-10.
46. Keene ON, Jones MR, Lane PW, et al. Analysis of exacerbation rates in asthma and chronic obstructive pulmonary disease: example from the TRISTAN study. *Pharmaceutical Statistics* 2007;6:89-97.
47. Sach TH, Dsborough J, Houghton J, et al. Applying microcosting methods to estimate the costs of pharmacy interventions: an illustration using

For peer review only - http://bmjopen.bmj.com/site/about/guidelines.xhtml

multiprofessional clinical medication reviews in care homes for older people. *International Journal of Pharmacy Practice* 2015;23:237-47.
48. Lopetegui M, Yen PY, Lai A, et al. Time motion studies in healthcare: what are we talking about? *Journal of Biomedical Informatics* 2014;49: 292-99.
49. Piccenna L, Walker W, Shears G, et al. Priorities for epilepsy research: A briefing report 2017 [Available from: https://pdfs.semanticscholar.org/5861/867345dc168e35918e386e7beaa89c7c6586.pdf accessed 23rd October 2019.
50. Zarit SH, Reever KE, Bach-Peterson J. Relatives of the impaired elderly: correlates of feelings of burden. *Gerontologist* 1980;20:649-55.
51. Crawford JR, Henry JD, Crombie C, et al. Normative data for the HADS from a large non-clinical sample. *British Journal of Clin Psychology* 2001;40(4):429-34.
52. Walters SJ, Bonacho Dos Anjos Henriques-Cadby I, Bortolami O, et al. Recruitment and retention of participants in randomised controlled trials: a review of trials funded and published by the United Kingdom Health Technology Assessment Programme. *BMJ Open* 2017;7:e015276.
53. Monfared AA, Lelorier J. Accuracy and validity of using medical claims data to identify episodes of hospitalizations in patients with COPD. *Pharmacoepidemiology and drug safety* 2006;15:19-29.
54. Roberts RO, Bergstralh EJ, Schmidt L, et al. Comparison of self-reported and medical record health care utilization measures. *Journal of Clinical Epidemiology* 1996;49:989-95.
55. Medical Research Council. Obtaining data from NHS Digital for health research – a guide for researchers 2016 [Available from: https://mrc.ukri.org/documents/pdf/obtaining-data-from-nhs-digital-v101016/ accessed 10th October 2016.
56. Raftery J, Young A, Stanton L, et al. Clinical trial metadata: defining and extracting metadata on the design, conduct, results and costs of 125 randomised clinical trials funded by the National Institute for Health Research Health Technology Assessment programme. *Health Technology Assessment* 2015;19:1-138.
57. National Institute for Health Research. HTA Supporting Information: Reasonable costs and value for money 2019 [Available from: https://www.nihr.ac.uk/documents/hta-supporting-information/11929#Reasonable_costs_and_value_for_money accessed 22nd October 2019.
58. Ridsdale L, Wojewodka G, Robinson EJ, et al. The effectiveness of a group self-management education course for adults with poorly controlled epilepsy, SMILE (UK): A randomized controlled trial. *Epilepsia* 2018 59(5):1048-61.
59. Ridsdale L, Wojewodka G, Robinson E, et al. Characteristics associated with quality of life among people with drug-resistant epilepsy. *Journal of Neurology* 2017;246(6):1174-84.
60. Hewitson P, Ward AM, Heneghan C, et al. Primary care endorsement letter and a patient leaflet to improve participation in colorectal cancer screening: results of a factorial randomised trial. *British Journal of Cancer* 2011;105(4):475-80.
61. Sajatovic M, Colon-Zimmermann K, Kahriman M, et al. A 6-month prospective randomized controlled trial of remotely delivered group format epilepsy self-management versus waitlist control for high-risk people with epilepsy. *Epilepsia* 2018 59(9):1684-95.

For peer review only - http://bmjopen.bmj.com/site/about/guidelines.xhtml

62. Luedke MW, Blalock DV, Goldstein KM, et al. Self-management of epilepsy: A systematic review. *Annals of Internal Medicine* 2019 171(2):117-26.
63. Thabane L, Ma J, Chu R, et al. A tutorial on pilot studies: the what, why and how. *BMC Medical Research Methodology* 2010;10(1)
64. Eldridge SM, Chan CL, Campbell MJ, et al. CONSORT 2010 statement: extension to randomised pilot and feasibility trials. *British Medical Journal* 2016;355:i5239.
65. Thapar A, Kerr M, Harold G. Stress, anxiety, depression, and epilepsy: Investigating the relationship between psychological factors and seizures. *Epilepsy and Behavior* 2009;14:134-40.
66. Cramer J, Perrine K, Devinsky O, et al. Development and cross-cultural translations of a 31-item quality of life in epilepsy inventory. *Epilepsia* 1998;39:81-88.
67. Zigmond AS, Snaith RP. The hospital anxiety and depression scale. *Acta psychiatrica Scandinavica* 1983;67:361-70.
68. Taylor J, Baker GA, Jacoby A. Levels of epilepsy stigma in an incident population and associated factors. *Epilepsy and Behavior* 2011;21(255-260)
69. Mittan R. Fear of seizures. In: Whitman S, Hermann B, eds. Psychopathology in epilepsy: social dimensions New York, NY: Oxford University Press 1986:90–121.
70. Martiniuk ALC, Campbell MK. Development and psychometric properties of the Thinking about Epilepsy questionnaire assessing children’s knowledge and attitudes about epilepsy. *Epilepsy and Behavior* 2007;10:595-603.
71. Wagner AK, Keller SD, Kosinski M, et al. Advances in methods for assessing the impact of epilepsy and antiepileptic drug therapy on patients' health-related quality of life. *Qual of Life Research* 1995;4:115-34.
72. Shore CP, Austin JK, Dunn DW. Maternal adaptation to a child’s epilepsy. *Epilepsy and Behavior* 2004;5:557-68.
73. Beecham J, Knapp M. Costing psychiatric interventions. In: Thornicroft G, Brewin C, Wing J, eds. Measuring Mental Health Needs. London: Gaskell 1992: 163-83.
74. Williams A. The role of the EUROQOL instrument in QALY calculations. York: Centre for Health Economics, University of York, 1995.
75. Smyth RM, Duley L, Jacoby A, et al. Women's experiences of participating in the Magpie Trial: a postal survey in the United Kingdom. *Birth* 2009;36:220-29.
76. Vuong Q. Likelihood ratio tests for model selection and non-nested hypotheses. *Econometrica* 1989;B:307- 33.

For peer review only - http://bmjopen.bmj.com/site/about/guidelines.xhtml

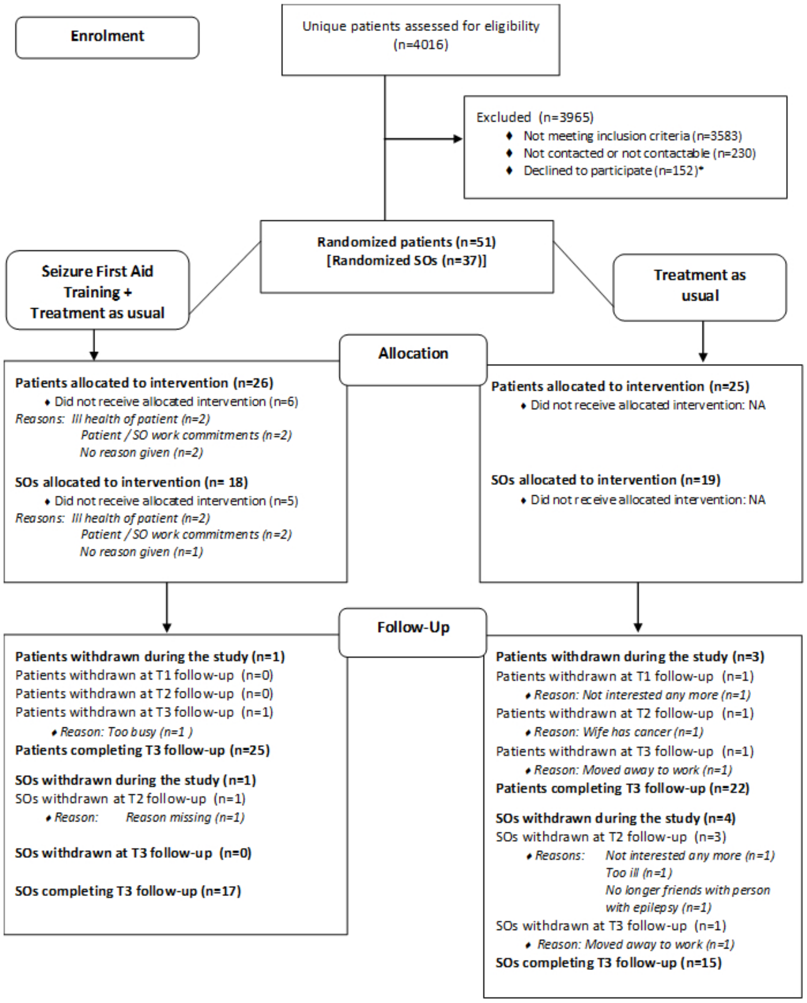

Figure 1 CONSORT diagram of flow through the pilot trial

60x88mm (300 x 300 DPI)

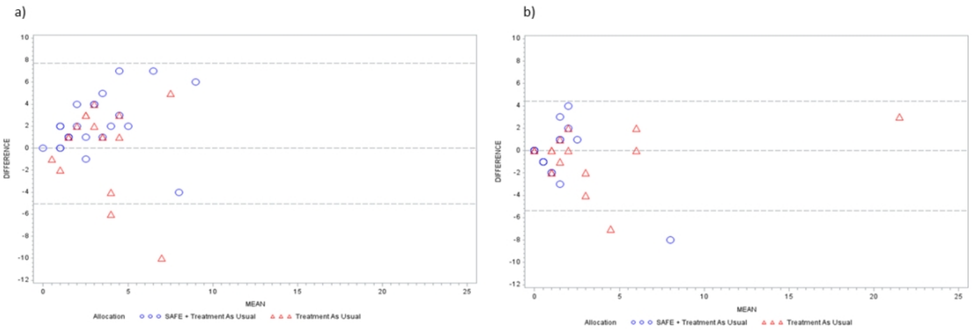

Figure 2 a) Bland Altman plot of agreement between self-reported ED use and routine data on ED use in 12 months prior to randomisation; b) Bland Altman plot of agreement between self-reported ED visits and routine data on ED use over 12 months following randomisation.

108x60mm (300 x 300 DPI)

SUPPLEMENTARY FILES

Supplementary File 1 Search criteria employed at the different recruitment sites to examine their electronic unique attendance record systems to identify potentially suitable persons attending ED for epilepsy

Recruitment site		
**Site**<br>**Aintree University Hospital**	**Site**<br>**Royal Liverpool University Hospital**	**Site**<br>**Arrowe Park Hospital**
Identify those persons who within the free text box 'presenting complaint' are recorded as having visited the ED for a:<br>• "fit"<br>• "epilepsy"<br>• "convulsion"<br>• "seizure"<br><br>And those patients admitted as an emergency from ED to a ward with any of the following primary or secondary ICD-10 diagnoses:<br>• "G40 Epilepsy"<br>• "G41 Status epilepticus"<br>• "G83.8 Other specified paralytic syndromes"<br>• "R56.8 Other and unspecified convulsions"	Identify those persons who attended the ED and were given any of the following presenting complaint-discharge diagnosis profiles:<br>• Presenting complaint "AE20 AEPRC Fit" AND ED discharge diagnosis "02 AEDIG Contusion/ abrasion";<br>• "AE20 AEPRC Fit" AND "04 AEDIG Head injury";<br>• "AE20 AEPRC Fit" AND "05 AEDIG Dislocation";<br>• "AE20 AEPRC Fit" AND "06 AEDIG Sprain/ ligament injury";<br>• "AE20 AEPRC Fit" AND "07 AEDIG Muscle/ tendon injury";<br>• "AE20 AEPRC Fit" AND "10 AEDIG Burns and scalds";<br>• "AE20 AEPRC Fit" AND "15 AEDIG Near Drowning";<br>• "AE20 AEPRC Fit" AND "33 AEDIG Facio-maxillary conditions";<br>• "AE20 AEPRC Fit" AND "38 AEDIG Diagnosis not classifiable".<br>• "241 AEDIG Epilepsy".<br><br>And those patients admitted as an emergency from ED to a ward with any of the following primary or secondary ICD-10 diagnoses:<br>• "G40 Epilepsy"<br>• "G41 Status epilepticus"<br>• "G83.8 Other specified paralytic syndromes"<br>• "R56.8 Other and unspecified convulsions"	Identify those persons who within the free text box 'reason for visit' or 'presenting complaint' are recorded as having visited the ED for:<br>• "fit"<br>• "epilep"<br>• "convuls"<br>• "seiz"<br><br>And those patients admitted as an emergency from ED to a ward with any of the following primary or secondary ICD-10 diagnoses:<br>• "G40 Epilepsy"<br>• "G41 Status epilepticus"<br>• "G83.8 Other specified paralytic syndromes"<br>• "R56.8 Other and unspecified convulsions"

Notes:

As the architectures of the systems and the coding processes at each sites varied the research team worked with local ED teams to identify the search criteria.

For peer review only

Supplementary File 2 CONSORT diagram of eligibility screening for the trial

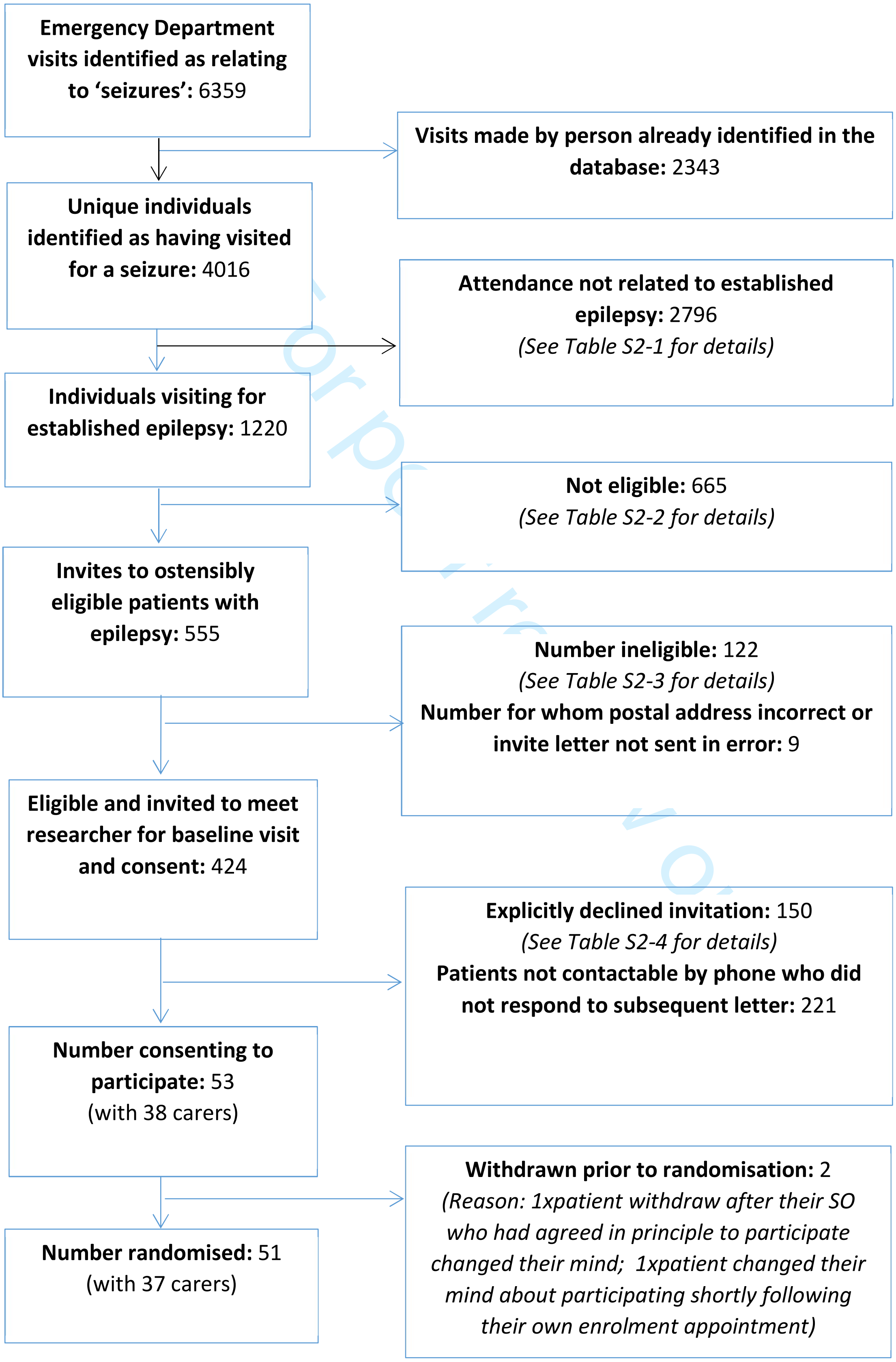

Table S2-1 Attendance at emergency department not related to epilepsy

Reason	Number of participants			
	Site 1	Site 2	Site 3	Total
No confirmed diagnosis of epilepsy/not attending because of epilepsy	737	483	715	1935
Acute symptomatic seizures related to neurological illness or substance abuse	238	240	191	669
Psychogenic non-epileptic seizures	19	55	29	103
Medical record missing	54	5	27	86
Ineligible, but no reason given	2	1	0	3
**Total**	**1050**	**784**	**962**	**2796**

Table S2-2 Other reasons for non-eligibility (individuals visiting for established epilepsy)

Reason	Number of participants			
	Site 1	Site 2	Site 3	Total
Postcode outside catchment area	148	94	60	302
Learning disability likely to impede individuals' capacity to provided signed, informed consent	45	51	52	148
Inability to converse in English and provide signed informed consent	10	24	46	80
Life threatening medical illness	35	26	10	71
Severe psychiatric disorder	11	3	18	32
No fixed abode	10	6	13	29
Participating in another trial	3	0	0	3
**Total**	**262**	**204**	**199**	**665**

Table S2-3 Reasons for non-eligibility (ostensibly eligible individuals)

Reason	Number of participants			
	Site 1	Site 2	Site 3	Total
Has not visited an ED for epilepsy on ≥2 occasions within previous 12 months (as reported by patient)	9	15	24	48
Not able to provide informed consent, participate in SAFE course if randomised or to independently complete questionnaires in English	9	7	7	23
Ineligible, but no reason given[a]	4	7	4	15
Not established diagnosis of epilepsy (<1 year)	3	2	5	10
Moved out of area; postcode no longer within 25 mile catchment area	4	1	3	8
Actual or suspected psychogenic non-epileptic seizures alone or in combination with epilepsy	2	1	4	7
Severe current psychiatric disorders or life-threatening medical illness	1	5	1	7

Not currently being prescribed AED/s	1	0	1	2
Acute symptomatic seizures related to acute neurological illness or substance misuse	0	1	0	1
Participating in another trial	1	0	0	1
**Total**	**34**	**39**	**49**	**122**

Notes:

a. Lack of reason due to patients returning participation slips and no stating actual reason for ineligibility

Table S2-4 Reasons for declining participation (eligible individuals)

**Reason**	**Number of participants**			
	**Site 1**	**Site 2**	**Site 3**	**Total**
Not interested	30	11	23	64
Too busy	11	12	11	34
No reason given	9	10	8	27
Too ill	5	5	9	19
Too well	3	1	2	6
**Total**	**58**	**39**	**53**	**150**

For peer review only

Supplementary File 3 Demographic characteristics of SO participants

Demographic characteristic	Seizure First Aid Training + TAU	Treatment as usual (TAU)	Total
Relationship with patient participant	(n=18)	(n=19)	(n=37)
Parent	4 (22.2%)	4 (21.1%)	8 (21.6%)
Son / daughter	2 (11.1%)	4 (21.1%)	6 (16.2%)
Grandparent	0 (0.0%)	0 (0.0%)	0 (0.0%)
Spouse / partner	9 (50.0%)	7 (36.8%)	16 (43.2%)
Sibling	1 (5.6%)	0 (0.0%)	1 (2.7%)
Cousin, aunt / uncle	0 (0.0%)	0 (0.0%)	0 (0.0%)
Niece / nephew	0 (0.0%)	1 (5.3%)	1 (2.7%)
Friend	2 (11.1%)	3 (15.8%)	5 (13.5%)
Other	0 (0.0%)	0 (0.0%)	0 (0.0%)
Missing	0 (0.0%)	0 (0.0%)	0 (0.0%)
Co-habitation with patient participant	(n=18)	(n=19)	(n=37)
Yes	14 (77.8%)	14 (73.4%)	28 (75.7%)
No	4 (22.2%)	5 (26.3%)	9 (24.3%)
Missing	0 (0.0%)	0 (0.0%)	0 (0.0%)
Contact with patient participant (number of days per week)	(n=18)	(n=19)	(n=37)
None	0 (0.0%)	0 (0.0%)	0 (0.0%)
One	1 (5.6%)	0 (0.0%)	1 (2.7%)
Two	0 (0.0%)	1 (5.3%)	1 (2.7%)
Three	0 (0.0%)	2 (10.5%)	2 (5.4%)
Four	0 (0.0%)	0 (0.0%)	0 (0.0%)
Five	0 (0.0%)	0 (0.0%)	0 (0.0%)
Six	0 (0.0%)	0 (0.0%)	0 (0.0%)
Seven (every day)	17 (94.4%)	16 (84.2%)	33 (89.2%)
Missing	0 (0.0%)	0 (0.0%)	0 (0.0%)
Sex: n (%)	(n=18)	(n=19)	(n=37)
Male	6 (33.3%)	9 (47.4%)	15 (40.5%)
Female	12 (67.7%)	10 (52.6%)	22 (59.5%)
Missing	0 (0.0%)	0 (0.0%)	0 (0.0%)
Age at consent into the trial (years)			
N	17 (94.4%)	19 (100.0%)	36 (94.6%)
Mean	41.3	44.9	43.2
Standard deviation	18.66	15.69	17.01
Minimum	17.8	18.1	17.8
Median	43.5	49.7	48.0
Maximum	79.7	71.5	79.7
Missing	1 (5.6%)	0 (0.0%)	1 (2.7%)
Ethnicity: n (%)	(n=18)	(n=19)	(n=37)
White	18 (100.0%)	18 (94.5%)	36 (97.3%)
Asian / Asian British	0 (0.0%)	0 (0.0%)	0 (0.0%)
Black / African / Carribean / Black British	0 (0.0%)	0 (0.0%)	0 (0.0%)
Mixed / multiple ethnic groups	0 (0.0%)	0 (0.0%)	0 (0.0%)
Other ethnic group	0 (0.0%)	1 (5.3%)	1 (2.7%)
Missing	0 (0.0%)	0 (0.0%)	0 (0.0%)
Significant medical history: n (%)	(n=18)	(n=19)	(n=37)
No, none	13 (72.2%)	12 (63.2%)	25 (67.6%)
Yes, a medical condition	5 (27.8%)	6 (31.6%)	11 (29.7%)
Yes, a psychiatric condition	0 (0.0%)	0 (0.0%)	0 (0.0%)
Yes, both medical and psychiatric conditions	0 (0.0%)	1 (5.3%)	1 (2.7%)
Missing	0 (0.0%)	0 (0.0%)	0 (0.0%)

Education: n (%)	(n=18)	(n=19)	(n=37)
O' levels/ GCSEs/ Level 1 or 2 NVQ	9 (50.0%)	14 (73.7%)	23 (62.2%)
A' Levels/ Level 3 NVQ	4 (22.2%)	2 (10.5%)	6 (16.2%)
University degree/ Graduate Certificate or Diploma	5 (27.8%)	3 (15.8%)	8 (21.6%)
Postgraduate university degree (e.g., PGCE, MSc, MA, PhD)	0 (0.0%)	0 (0.0%)	0 (0.0%)
Missing	0 (0.0%)	0 (0.0%)	0 (0.0%)

For peer review only

Supplementary File 4 Measure completeness by assessment point, measure and participant type

Table S4.1 Number of patient participants completing assessment tools

Study assessment tool and timepoint	SAFE + TAU	TAU	Total
**Patient Participants: Baseline (T0)**	**(n=26)**	**(n=25)**	**(n=51)**
ED visits (self-report)	24 (92.3%)	21 (84.0%)	45 (88.2%)
Seizure control: Thapar scale	26 (100%)	25 (100%)	51 (100%)
Quality of Life: QOLIE-31P	23 (88.5%)	16 (64.0%)	39 (76.5%)
Distress: HADS [a]	23 (88.5%)	24 (96.0%)	47 (92.2%)
Felt stigma: Stigma of Epilepsy Scale	24 (92.3%)	24 (96.0%)	48 (94.1%)
Health economics: Client Service Receipt Inventory [b]	19 (73.1%)	17 (68.0%)	36 (70.6%)
Health economics: EuroQol-5D	21 (80.8%)	25 (100.0%)	46 (90.2%)
Confidence managing seizures/ epilepsy: Epilepsy Mastery Scale	26 (100.0%)	21 (84.0%)	47 (92.2%)
Fear of seizures:, Epilepsy Knowledge and Management Questionnaire Fears subscale	16 (61.5%)	9 (36.0%)	25 (49.2%)
Knowledge of what to do: Thinking about Epilepsy Questionnaire	26 (100%)	25 (100%)	51 (100%)
**Patient Participants: 6 months (T2)**	**(n=26)**	**(n=23)[c]**	**(n=49)[c]**
Quality of Life: QOLIE-31P	16 (61.5%)	12 (52.2%)	28 (57.1%)
Confidence managing seizures/ epilepsy: Epilepsy Mastery Scale	21 (80.8%)	15 (65.2%)	36 (73.5%)
Seizure control: Thapar scale	16 (61.5%)	15 (65.2%)	28 (57.1%)
**Patient Participants: 12 months (T3)**	**(n=25)[d]**	**(n=22)[d]**	**(n=47)[d]**
ED visits (self-report)	17 (68.0%)	17 (77.3%)	34 (72.3%)
Seizure control: Thapar scale	20 (80.0%)	14 (60.9%)	34 (72.3%)
Quality of Life: QOLIE-31P	10 (40.0%)	8 (36.4%)	18 (38.3%)
Distress: HADS [a]	18 (72.0%)	17 (77.3%)	35 (74.4%)
Felt stigma: Stigma of Epilepsy Scale	18 (72.0%)	17 (77.3%)	35 (74.4%)
Health economics: Client Service Receipt Inventory [b]	13 (52.0%)	10 (45.5%)	23 (48.9%)
Health economics: EuroQol-5D	18 (72.0%)	17 (77.3%)	35 (74.4%)
Confidence managing seizures/ epilepsy: Epilepsy Mastery Scale	18 (72.0%)	16 (72.7%)	34 (72.3%)
Fear of seizures: Epilepsy Knowledge and Management Questionnaire Fears subscale	8 (32.0%)	5 (22.7%)	13 (27.7%)
Knowledge of what to do: Thinking about Epilepsy Questionnaire	18 (72.0%)	17 (77.3%)	35 (74.4%)
Feedback on participation	17 (68.0%)	15 (68.2%)	32 (68.1%)

Notes:

[a] Completeness of the whole HADS scale (both anxiety and depression subscales)

[b] Only six mandatory questions counted. Conditional questions (e.g. if yes, then…) not counted towards total completion

[c] Two patient participants from the TAU group had withdrawn by the T2 visit

[d] Four patient participants (three from the TAU group and one from the SAFE + TAU group) had withdrawn by the T3 visit

[e] Note that whilst some participants did not fully complete all items within a questionnaire, this does not automatically mean their data would need to be excluded from analysis of a change in that domain since for some measures test developers permit imputation when the extent of missing items is low and follows a specific pattern.

For peer review only

Table S4.2 Number of SO participants completing study assessment tools

Study assessment tool and time point	SAFE + TAU	TAU	Total
**Significant other Participants: Baseline (T0)**	**(n=18)**	**(n=19)**	**(n=37)**
Burden: Zarit Caregiver Burden	17 (94.4%)	17 (89.5%)	34 (91.9%)
Distress: HADS [a]	17 (94.4%)	18 (94.7%)	35 (94.6%)
Confidence managing seizures/ epilepsy: Parent Response to Child Illness Scale	18 (100.0%)	19 (100.0%)	37 (100.0%)
Fear of seizures: Epilepsy Knowledge and Management Questionnaire Fears subscale	6 (33.3%)	4 (21.1%)	10 (27.0%)
Knowledge of what to do: Thinking about Epilepsy Questionnaire	18 (100.0%)	19 (100.0%)	37 (100.0%)
**Significant other Participants: 6 months (T2)**	**(n=17)[b]**	**(n=16)[b]**	**(n=33)[b]**
Burden: Zarit Caregiver Burden	15 (88.2%)	8 (50.0%)	23 (69.7%)
Confidence managing seizures/ epilepsy: Parent Response to Child Illness Scale	16 (94.1%)	9 (56.3%)	25 (75.8%)
**Significant other Participants: 12 months (T3)**	**(n=17)[c]**	**(n=15)[c]**	**(n=32)[c]**
Burden: Zarit Caregiver Burden	11 (64.7%)	10 (66.7%)	21 (65.6%)
Distress: HADS [a]	11 (64.7%)	10 (66.7%)	21 (65.6%)
Confidence managing seizures/ epilepsy: Parent Response to Child Illness Scale	11 (64.7%)	10 (66.7%)	21 (65.6%)
Fear of seizures: Epilepsy Knowledge and Management Questionnaire Fears subscale	6 (35.3%)	2 (13.3%)	8 (25.0%)
Knowledge of what to do: Thinking about Epilepsy Questionnaire	15 (88.2%)	13(86.7%)	28 (87.5%)
Feedback on participation	11 (64.7%)	9 (60.0%)	20 (62.5%)

Notes:

[a] Completeness of the whole HADS scale (both anxiety and depression subscales),

[b] Four SO participants (one in the SAFE group and three in the TAU group) had withdrawn by the T2 visit

[c] Five SO participants (one in the SAFE group and four in the TAU group) had withdrawn by the T3 visit

[d] Note that whilst some participants did not fully complete all items within a questionnaire, this does not automatically mean their data would need to be excluded from analysis of a change in that domain since for some measures test developers permit imputation when the extent of missing items is low and follows a specific pattern.

For peer review only

Supplementary File 5 Histogram of number of emergency department attendances in 12 prior to randomisation by n=48 patient participants according to routine data

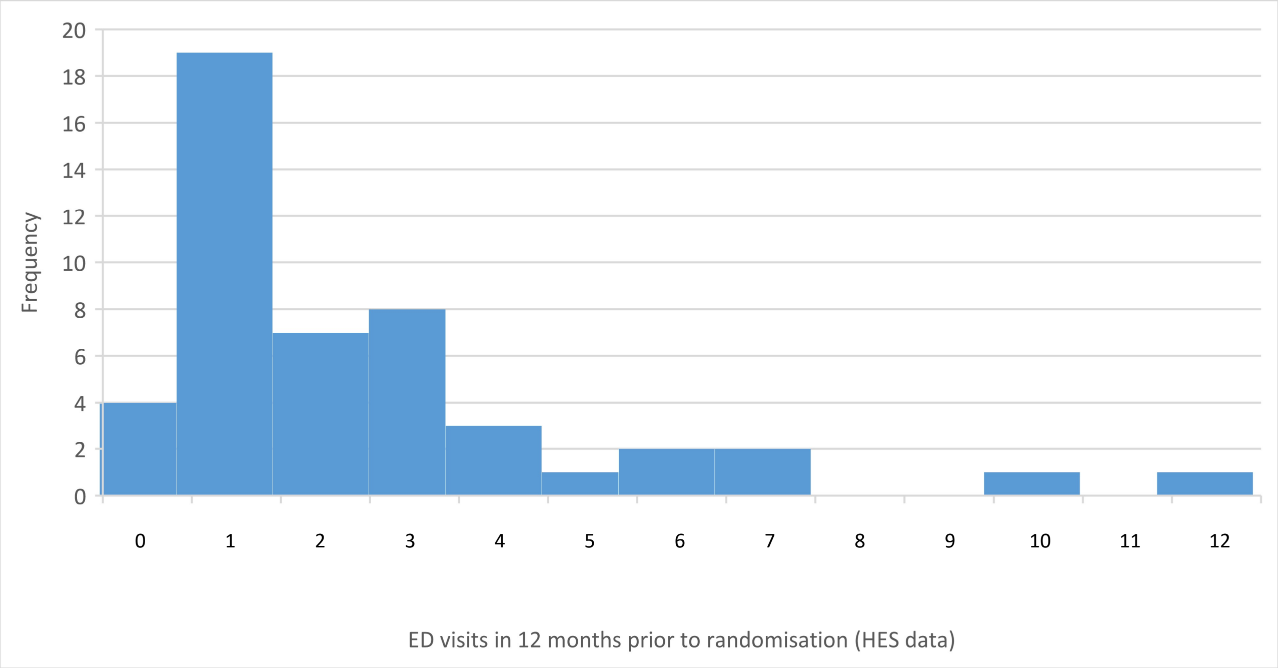

review only

Supplementary File 6 Summary of key milestones in securing ED outcome data from NHS Digital

Period	Project milestone		Milestone in obtaining data from NHS Digital	
	Date	*Note*	*Date*	*Note*
**Period**			**28 Sep 2015**	Received feedback on draft Participant Information Sheets and Consent form for trial to ensure compliance.
Trial period	**19 May 2016**	First patient participant randomised		
			**11 Oct 2017**	Participated in NHS Digital training.
			**18 Oct 2017**	Registered as new user of application system.
			**07 Nov 2017**	Delays experienced. Access to application form granted after chasing.
	**Dec 2017**	Final 12-month follow-up completed		
Post -trial			**16 Feb 2018**	Application submitted to NHS Digital
			**28 Feb 2018**	Teleconference with NHS Digital. Provisional opinion given that only minor changes required and likely timeframe to receipt of data 1.5 months.
			**07 Mar 2018**	Received written feedback from NHS Digital after chasing on minor changes required.
			**22 Mar 2018**	Revised application submitted.
			**03 Apr 2018**	NHS Digital submit new query (1) to applicants requesting additional information. (Applicants respond 03 Apr 2018)
			**26 Apr 2018**	NHS Digital submit new query (2) to applicants requesting additional information. (Applicants respond 26 Apr 2018)
			**30 Apr 2018**	NHS Digital submit new query (3) to applicants requesting additional information. (Applicants respond 30 Apr 2018)
			**05 May 2018**	NHS Digital submit new query (4) to applicants requesting additional information. (Applicants respond 05 May 2018).
			**10 May 2018**	NHS Digital submit new query (5) to applicants requesting additional information (in light of new General Data Protection Regulation introduced on May 2018). (Applicants respond 11 May 2018)
			**30 May 2018**	NHS Digital submit new query (6) to applicants requesting additional information (in light of new General Data Protection Regulation introduced on May 2018). (Applicants respond 30 May 2018)
			**30 May 2018**	NHS Digital submit new query (7) to applicants requesting additional information. (Applicants respond 30 May 2018)
			**15 Jun 2018**	NHS Digital confirm Data Approvals Owner will review revised application.
			**27 Jun 2018**	NHS Digital submit new query (8) to applicants requesting additional information and request revisions to trial website in light of new General Data Protection Regulation introduced on May 2018). (Applicants respond 28 Jun 2018)

			**02 Jul 2018**	NHS Digital submit new query (9) to applicants requesting additional information.<br>(Applicants 02 Jul 2018)
			**06 Jul 2018**	NHS Digital notify applicants that Data Approval Owner has rejected application, primarily because: "concerns [over] whether this pilot would yield findings that were statistically valuable to achieve the stated aims given the small numbers".
			**10 Jul 2018**	Secured confirmation of right to appeal and process.
			**20 Jul 2018**	Applicants submit letter of appeal.
			**25 Jul 2018**	Appeal accepted by NHS Digital.
			**15 Aug 2018**	NHS Digital submit new query (10) to applicants following Independent Group Advising on the Release of Data (IGARD) committee's review of application.<br>(Applicants respond 16 Aug 2018)
			**16 Aug 2018**	Teleconference with NHS Digital data production team.
			**04 Sep 2018**	NHS Digital send Data Sharing Agreement to applicants.
			**10 Sep 2018**	NHS Digital confirm receipt of completed Data Sharing Agreement.
			**25 Sep 2018**	Applicants securely transfer patient participants' details to NHS Digital.
			**31 Sep 2018**	NHS Digital release data to applicants.

For peer review only

Supplementary File 7 Adverse events occurring during pilot trial in descending order according to frequency overall [a]

Adverse event		SAFE + TAU		TAU		Total	
Category of events (e.g. body system)	Event	Events (n)	Patients [n(%)]	Events (n)	Patients [n(%)]	Events (n)	Patients [n(%)]
Eyes, ear, nose, throat	Problem with eyes and sinuses	0	0 (0.0%)	1	1 (9.1%)	1	1 (5.6%)
Genito-urinary	Overnight hospital admission required due to urinary tract infection (pre existing)	0	0 (0.0%)	1	1 (9.1%)	1	1 (5.6%)
Haematological	Dislocated shoulder	0	0 (0.0%)	1	1 (9.1%)	1	1 (5.6%)
Neoplasia	Change in seizure pattern [b]	1	1 (14.3%)	0	0 (0.0%)	1	1 (5.6%)
	Increase in seizure frequency [b]	1	1 (14.3%)	0	0 (0.0%)	1	1 (5.6%)
Neurological	Increased seizures, medications changed	1	1 (14.3%)	3	3 (27.3%)	4	4 (22.2%)
	Increased seizures (shift patterns at work)	1	1 (14.3%)	0	0 (0.0%)	1	1 (5.6%)
	New seizure type; frequent absence seizures as well as usual seizure types	1	1 (14.3%)	0	0 (0.0%)	1	1 (5.6%)
	Seizure frequency sodium levels requiring in patient monitoring	0	0 (0.0%)	1	1 (9.1%)	1	1 (5.6%)
	Seizures more severe (tonic clonic)	0	0 (0.0%)	1	1 (9.1%)	1	1 (5.6%)
	Vagus Nerve Stimulation not working properly, going to hospital for observation	0	0 (0.0%)	1	1 (9.1%)	1	1 (5.6%)
Respiratory	Increases number of seizures due to chest infection and antibiotics	0	0 (0.0%)	1	1 (9.1%)	1	1 (5.6%)
Other:	Gynae-pregnancy. Increase in the number of fits due to being pregnant / just given birth	0	0 (0.0%)	2	1 (9.1%)	2	1 (5.6%)
	Diagnosis of status epilepticus	1	1 (14.3%)	0	0 (0.0%)	1	1 (5.6%)
	Vertigo	0	0 (0.0%)	1	1 (9.1%)	1	1 (5.6%)
**Total**		6	6 (100.0%)	13	11 (100.0%)	19	18 (100.0%)

Notes:

[a] Eighteen (35.3%) patient participants reported adverse events (AE) over the course of follow-up. In the SAFE+TAU group, 6 AEs were reported by 6 participants, while 13 AEs were reported by 12 participants from the TAU group. Only one SAE occurred during the study – namely, a diagnosis of previous status epilepticus. This was reported by one SAFE+TAU group participant. A medical review asserted it unlikely to be related to participation as it arose from investigations the participant had been having before participation.

[b] Two participants were under investigation to determine whether a brain tumour could be the cause of their seizure changes; hence 'Body System' is classified as 'Neoplasia' rather than 'Neurological.'

For peer review only

Supplementary File 8 Total observed costs and cost per delegate to deliver the seizure first aid intervention (SAFE) within a research trial with two academic researchers, one Epilepsy Specialist Nurse delivering the training, and administrative support.

**Description**	**Design & set-up**	**Practice Session 1**	**Practice Session 2**	**SAFE+TAU Group 1**	**SAFE+TAU Group 2**	**SAFE+TAU Group 3**	**SAFE+TAU Group 4**	**SAFE+TAU Group 5**	**SAFE+TAU Group 6**	**SAFE+TAU Group 7**	**TAU Group 1**	**TAU Group 2**
Fixed costs (£)												
Equipment	928.85	0.00	0.00	0.00	0.00	0.00	0.00	0.00	0.00	0.00	0.00	0.00
Website	77.12	0.00	0.00	0.00	0.00	0.00	0.00	0.00	0.00	0.00	0.00	0.00
Freepost licence	116.40	0.00	0.00	0.00	0.00	0.00	0.00	0.00	0.00	0.00	0.00	0.00
Venue	0.00	80.00	80.00	80.00	80.00	80.00	80.00	80.00	80.00	80.00	80.00	80.00
Facilitator (staff cost)		192.79	192.79	177.96	177.96	177.96	177.96	177.96	177.96	177.96	177.96	177.96
Facilitation resources		4.96	4.96	4.96	4.96	4.96	4.96	4.96	4.96	4.96	4.96	4.96
Total (fixed)	1122.37	277.75	277.75	262.92	262.92	262.92	262.92	262.92	262.92	262.92	262.92	262.92
Variable costs (£)												
Staff costs	4243.41	612.88	1158.56	591.19	521.84	429.37	429.37	318.41	318.41	318.41	299.96	416.03
Staff travel expenses	44.10	279.87	279.87	274.67	274.67	265.43	265.43	265.43	265.43	265.43	233.63	265.41
Participant expenses	0.00	180.00	200.00	40.00	40.00	40.00	40.00	40.00	40.00	40.00	92.80	0.00
Office costs (inc. telephone)	589.33	33.00	105.50	81.63	85.23	92.60	88.70	89.00	86.60	89.00	57.17	70.13
Stationery, print, post	0.00	156.35	196.96	57.44	93.68	68.93	68.93	34.46	22.98	45.95	182.73	298.44
Refreshments	0.00	72.25	72.25	41.80	41.80	32.81	32.00	41.00	26.60	18.50	21.00	34.50
Advertising	45.00	0.00	0.00	0.00	0.00	0.00	0.00	0.00	0.00	0.00	0.00	0.00
Total (variable)	4921.84	1334.34	2013.13	1086.73	1057.22	929.13	924.42	788.30	760.01	777.29	887.28	1084.51
TOTAL COST (£)	6044.21	1612.09	2290.88	1349.65	1320.14	1192.05	1187.34	1051.22	1022.93	1040.21	1150.20	1347.43
Delegate (n) [patient:SO]		12	15	~~2:~~3:2	4:4	3:3	4:2	3:0	1:1	2:2	4:4	8:5

For peer review only

Mean cost/delegate (£)		134.34	152.73	269.93	165.02	198.68	197.89	350.41	511.47	260.05	143.7 8	103.65
Mean cost per group (sd)<br>95% central range *				£1,166 (134.22)<br>£1,023-£1,350							£1,249 (139.46)<br>£1,150-£1,347	
Mean cost per delegate<br>95% central range*				£240<br>£211-£278							£119<br>£110-£128	
Mean cost per patient (with or without SO)<br>95% central range*				£408<br>£358-£472							£208<br>£192-£225	

Notes:

*Central ranges generated from 10,000 bootstrap replications; The quantity of use of the resources, was measured. To capture the resources used the 'time and motion method' was used with AN, DS, JB and GM completing electronic data collection forms on which they self-reported the time they dedicated to different activities. The research team (AN, DS) also recorded details of the number of participants invited and attending each SAFE sessions and non-staff resources.

For peer review only

Supplemental File 9 Serious Adverse Event Protocol

Adapted from the Medicines for Human Use (Clinical Trials) Regulations 2004 (SI 2004/1031), http://www.legislation.gov.uk/uksi/2004/1031/pdfs/uksi_20041031_en.pdf [accessed 29th August, 2019] a serious adverse event (SAE) was defined as an adverse event which resulted in any of the following:

- Death
- Was life-threatening (subject at immediate risk of death, e.g., status epilepticus)
- Seizure resulting in hospital admission for ≥24 hours
- Emergency attendance or hospital admission for reason other than seizure
- Results in persistent or significant disability or incapacity, or
- Was otherwise considered medically significant.

'Life-threatening' in the definition of 'serious' referred to an event in which the patient was at risk of death at the time of the event; it did not refer to an event which hypothetically might have caused death if it were more severe. Hospitalisations for a pre-existing condition, including elective procedures that had not worsened, did not constitute an SAE. Prolongation of hospital stay due to social factors, for example, geographical location of the participant's home which prevented discharge was also not considered a SAE.

Given the characteristics of the subject population being studied, the following events were expected in this study population and were not recorded as part of the SAE monitoring process:

- Epileptic seizures with or without injury;
- Emergency or urgent medical attention. This includes visiting a hospital emergency department with the duration of the stay lasting <24 hours, attending an NHS out of hours primary care service (NHS Urgent Care 24), telephoning for an ambulance, telephoning NHS 111, seeking/ having an urgent/ fast-tracked appointment with a usual care provider (GP or specialist) or other registered health professional (e.g., a pharmacist);
- Side-effects of AED medication;
- Diagnosis of a comorbid psychiatric condition.

A delegated medically qualified person within the team (AM; LR) assessed each unexpected SAE. This person considered information on the temporal and physical relationship between the event and possible causes and assessed whether the event was related or unrelated to the patient's participation in the study. Further details on the process followed detailed below.

Monitoring

As part of this trial patient participants did not receive additional medical reviews. There is also no 'live' system which can be used to track SAEs such as emergency admissions and usual care providers are not systematically informed of them. Therefore, to monitor SAEs the research team liaised with patient participants themselves. A standardised form was to be completed at 3- (T1, by telephone), 6- (T2, by telephone) and 12-months (T3, during a face-to-face appointment) post-randomisation to collect information on patient participants' experience of unexpected, SAEs.

In each instance, a maximum of 3 attempts was made to contact the patient participant by telephone (including trying to contact them via their informal carer if they are taking part with one). If the patient was

For peer review only

not be contactable, a letter was be sent the patient's GP asking them to inform the research team if the patient was no longer alive and the circumstances of their death.

Causality

One of the delegated medically qualified persons within the team assessed each unexpected SAE. They considered information on the temporal and physical relationship between the event and possible causes and assessed whether the event was related or unrelated to the patient's participation in the study. In doing this, they used the definitions in the Table below.

Table Definitions of causality for serious adverse event

	**Description**
**Unrelated**	There is no evidence of any causal relationship. There is an alternative cause for the SAE.
**Unlikely**	There is little evidence to suggest there is a causal relationship (e.g. the event did not occur within a reasonable time after receipt of the intervention). There is another reasonable explanation for the event (e.g. the participant's clinical condition, other concomitant treatment).
**Possibly**	There is some evidence to suggest a causal relationship (e.g. because the event occurs within a reasonable time after receipt of the intervention). However, the influence of other factors may have contributed to the event (e.g. the participants clinical condition, other concomitant treatment).
**Probably**	There is evidence to suggest a causal relationship and the influence of other factors is unlikely.
**Almost certainly**	There is clear evidence to suggest a causal relationship and other possible contributing factors can be ruled out.

Reporting

As per National Research Ethics Service (2015) guidelines for non-CTIMPs, the main ethics committee approving the study and its Sponsor were informed within 15 days of the team becoming aware of any SAE that in the opinion of the medical reviewer was both unexpected (that is, the type of event is not listed in the protocol as an expected occurrence) and judged to be "possibly", "probably" or "almost certainly" related to participation in the study (that is, it resulted from administration of any of the research procedures, including the intervention).

For peer review only - http://bmjopen.bmj.com/site/about/guidelines.xhtml

CONSORT 2010 checklist of information to include when reporting a pilot or feasibility trial*

Section/Topic	Item No	Checklist item	Reported on page No
**Title and abstract**			
	1a	Identification as a pilot or feasibility randomised trial in the title	1
	1b	Structured summary of pilot trial design, methods, results, and conclusions (for specific guidance see CONSORT abstract extension for pilot trials)	2
**Introduction**			
Background and objectives	2a	Scientific background and explanation of rationale for future definitive trial, and reasons for randomised pilot trial	4-5
	2b	Specific objectives or research questions for pilot trial	5
**Methods**			
Trial design	3a	Description of pilot trial design (such as parallel, factorial) including allocation ratio	6 (sec. 2.2), 7 (sec.2.6)
	3b	Important changes to methods after pilot trial commencement (such as eligibility criteria), with reasons	6 (sec. 2.2)
Participants	4a	Eligibility criteria for participants	6-7 (sec. 2.2)
	4b	Settings and locations where the data were collected	6 (sec. 2.2)
	4c	How participants were identified and consented	6 (sec. 2.4) & Supplementary File 1
Interventions	5	The interventions for each group with sufficient details to allow replication, including how and when they were actually administered	7 (sec. 2.8), Table 1, and references 32 & 37.
Outcomes	6a	Completely defined prespecified assessments or measurements to address each pilot trial objective specified in 2b, including how and when they were assessed	7-8 (sec. 2.9)
	6b	Any changes to pilot trial assessments or measurements after the pilot trial commenced, with reasons	N/A.
	6c	If applicable, prespecified criteria used to judge whether, or how, to proceed with future definitive trial	7 (sec. 2.9)
Sample size	7a	Rationale for numbers in the pilot trial	9 (sec. 2.10)
	7b	When applicable, explanation of any interim analyses and stopping guidelines	N/A.
Randomisation:			

For peer review only

Sequence generation	8a	Method used to generate the random allocation sequence	7 (sec.2.6)
	8b	Type of randomisation(s); details of any restriction (such as blocking and block size)	7 (sec.2.6)
Allocation concealment mechanism	9	Mechanism used to implement the random allocation sequence (such as sequentially numbered containers), describing any steps taken to conceal the sequence until interventions were assigned	7 (sec.2.6)
Implementation	10	Who generated the random allocation sequence, who enrolled participants, and who assigned participants to interventions	7 (sec.2.6)
Blinding	11a	If done, who was blinded after assignment to interventions (for example, participants, care providers, those assessing outcomes) and how	7 (sec.2.6)
	11b	If relevant, description of the similarity of interventions	N/A.
Statistical methods	12	Methods used to address each pilot trial objective whether qualitative or quantitative	8-9 (sec. 2.10)
**Results**			
Participant flow (a diagram is strongly recommended)	13a	For each group, the numbers of participants who were approached and/or assessed for eligibility, randomly assigned, received intended treatment, and were assessed for each objective	9-10 (sec. 3.1), 10-11 (sec. 3.3), Figure 1
	13b	For each group, losses and exclusions after randomisation, together with reasons	10 (3.1), Figure 1
Recruitment	14a	Dates defining the periods of recruitment and follow-up	6 (sec. 2.1, 2.2)
	14b	Why the pilot trial ended or was stopped	N/A.
Baseline data	15	A table showing baseline demographic and clinical characteristics for each group	Table 4
Numbers analysed	16	For each objective, number of participants (denominator) included in each analysis. If relevant, these numbers should be by randomised group	Tables 4, 5, 6 and Supplementary File
Outcomes and estimation	17	For each objective, results including expressions of uncertainty (such as 95% confidence interval) for any estimates. If relevant, these results should be by randomised group	9-13 (sec. 3)
Ancillary analyses	18	Results of any other analyses performed that could be used to inform the future definitive trial	Supplementary Files
Harms	19	All important harms or unintended effects in each group (for specific guidance see CONSORT for harms)	12 (3.7), Supplementary File 9
	19a	If relevant, other important unintended consequences	N/A
**Discussion**			

For peer review only

Limitations	20	Pilot trial limitations, addressing sources of potential bias and remaining uncertainty about feasibility	16 (sec. 4.4)
Generalisability	21	Generalisability (applicability) of pilot trial methods and findings to future definitive trial and other studies	16 (sec. 4.4)
Interpretation	22	Interpretation consistent with pilot trial objectives and findings, balancing potential benefits and harms, and considering other relevant evidence	13-15 (sec. 4)
	22a	Implications for progression from pilot to future definitive trial, including any proposed amendments	15 (sec. 4.3)
**Other information**			
Registration	23	Registration number for pilot trial and name of trial registry	1
Protocol	24	Where the pilot trial protocol can be accessed, if available	6 (reference 34)
Funding	25	Sources of funding and other support (such as supply of drugs), role of funders	17
	26	Ethical approval or approval by research review committee, confirmed with reference number	6 (sec. 2.3)

Citation: Eldridge SM, Chan CL, Campbell MJ, Bond CM, Hopewell S, Thabane L, et al. CONSORT 2010 statement: extension to randomised pilot and feasibility trials. BMJ. 2016;355.
*We strongly recommend reading this statement in conjunction with the CONSORT 2010, extension to randomised pilot and feasibility trials, Explanation and Elaboration for important clarifications on all the items. If relevant, we also recommend reading CONSORT extensions for cluster randomised trials, non-inferiority and equivalence trials, non-pharmacological treatments, herbal interventions, and pragmatic trials. Additional extensions are forthcoming: for those and for up to date references relevant to this checklist, see www.consort-statement.org.

For peer review only
